# Supplementary material for: Electrically stimulated eccentric contraction during non-weight bearing knee bending exercise in the supine position increases oxygen uptake: A randomized, controlled, exploratory crossover trial
Source: PLoS One. 2021 Nov 18;16(11):e0259856. doi: 10.1371/journal.pone.0259856 (PMC8601547; doi:10.1371/journal.pone.0259856)
Supplement: S2 File — (DOCX) [file pone.0259856.s004.docx]

Title

The exploratory study of analysis of expired gas during knee bending exercise combined with transcutaneous electrical stimulation in healthy subjects

UMIN000039336

Title

The exploratory study of analysis of expired gas during knee bending exercise combined with transcutaneous electrical stimulation in healthy subjects

Background & Rationale

It is well known that atrophy of the musculoskeletal system due to disuse occurs among astronauts. For instance, atrophy of the muscles and bones of astronauts in weightlessness is evident. One the other hand, atrophy of of the musculoskeletal system occurs due to aging or inpatients because of weightlessness.

　Neuromuscular electrical stimulation (NMES) is widely used as a method to increase muscle strength and improve physical function even at a low-moderate exercise intensity. We have developed a compact training device named “hybrid training system” (HTS) that is designed to maintain the musculoskeletal system of astronauts by using an electrically stimulated antagonist to resist the volitional contraction (VC) of agonist muscles (Figure1). HTS has the following characteristics.

1) Both muscles (agonist and antagonist) are exercised.

2) A longitudinal compressive load is placed on the bone.

3) Voluntary contractions activate deep muscles.

4) Small device, portable.

Therefore, we think that it becomes the useful device by a future month and the long-term stay with the small spaceship.

**URL: https://www.jstage.jst.go.jp/article/tjem/221/1/221_1_77/_pdf/-char/en**

Figure１：Note that both the volitionally activated agonist and the electrically stimulated antagonist contract during joint motion. The result is that both muscles are exercised and that a longitudinal compressive load is placed on the bone.

Atrophy of the musculoskeletal system with aging is causes such as fall or activity restrictions. Furthermore, as for the lying-in bed for the treatment of such as the internal cancer, cardiovascular disease, or fragility fracture occurs the disuse syndrome. Those prevention is indispensable for the health promotion of elderly people. One of the prevention is exercise therapy. In general, strength training intensity is used at light to moderate, around 60% of 1 RM or 50%-100% of 10 RM. However, as for the elderly people or the inpatients, such exercise is difficult. Therefore, the knee bending exercise method that we could perform with the lying on the bed was devised. The exercise load is 2 Mets, but the exercise load does not reach exercise load of the activity of daily living (ADL). Therefore, the exercise method of the exercise load of ADL level that we can perform on the bed is necessary.

　HTS is a method that eliminates the disadvantages of both volitional exercise and NMES (Matsuse, et al. 2006). It has been reported that HTS is a new training technique that can increase both muscular strength and muscle mass (Iwasaki, et al. 2006) (Takano, et al. 2010) (Yanagi, et al. 2003) (Matsuse, et al. 2006). In addition, we can utilize HTS during many different types of exercise (e.g. knee extension exercise, squat and hip flexion, and cycling exercise) (Iwasaki, et al. 2006) (Takano, et al. 2010) (Takano, et al. 2016). These indicated the possibility that HTS could enhance exercise effect more effectively than VC alone. In our past study, we showed that agonist contractions to resist electrically stimulated antagonist contractions increased oxygen uptake by about 5% in cycling activity (38). Moreover, cycling exercise at moderate intensity combined with HTS increased oxygen uptake at an average of about 20 % (39). In this study, combining HTS with walking exercise at moderate intensity increased oxygen uptake by 8-9 %. Therefore, the exercise load added by HTS in walking exercise would increase metabolic cost in the same way as for cycling exercise. This is a potential advantage of HTS, which combines VC and NMES simultaneously, as not only mechanical stress but also metabolic cost is added by not only electrically stimulated muscle contractions but also VC. In other words, there is thought to be not only the muscular strength enhancement effect but also the aerobic exercise effect by combining HTS for aerobic exercise simultaneously^7^. Moreover, we showed that HTS could enhance exercise induced growth hormone when combined with knee bending exercise or cycling exercise as whole body effect (17) (18). In late years, it was reported that the improvement of the maximum oxygen intake and improvement of the exercise tolerability by combination of NMES and aerobic exercise^11-14^. Moreover, it was reported that the combination of NMES and aerobic exercise improve the oxygen intake and glycometabolism ^15-17^.

We evaluate the influence of the knee bending exercise with HTS with the lying position to make HTS adapted to the rehabilitation on the bed as well as space medicine.

The purpose of the present study is to compare the metabolic cost between knee bending exercise with and without HTS with the lying position by analyzing expired gas. We hypothesized that oxygen uptake would be greater when walking in combination with HTS than for walking without HTS.

Aim:

In this study, we will analyze expired gas during knee bending exercise with the lying position to compare the effect of knee bending exercise with the lying position augmented by HTS with knee bending exercise with the lying position without HTS. We will evaluate the relative advantages of exercise that combines HTS with knee bending exercise with the lying position on the augmented metabolic cost.

The primary outcome will be change in oxygen intake.

The secondary outcomes will be change in heart rate and carbon dioxide output.

Study Hypotheses: Compared with knee bending exercise with HTS will:

#1 increase oxygen intake.

#2 increase heart rate and carbon dioxide output.

Exploratory Hypotheses:

#3 increase metabolic cost.

#4 increase energy consumed.

#5 increase exercise load.

Study period:

2020/4/1－ 2021/8/1

Study location: Study procedures will take place in the training room at Kurume University Hospital (the third floor).

Study population:

Inclusion Criteria

There will be a total of 20 participants recruited to participate in this study.

1. 20 years old or older and younger than 50 years old
2. Nonsmokers

Exclusion Criteria

1. Severe heart failure, renal failure, respiratory failure, cerebrovascular disease, and malignant disorder
2. Inflammatory disorder
3. Impossible of a knee bending exercise

Sample Size Estimate

We set 20 as the sample size for an exploratory study.

Interventions:

Intervention will occur for 10 minutes per session, 1 time a week for 2 weeks (total of 2 sessions). Each session will be separated by an interval of at least 7 days. The subjects perform the knee bending exercise with the lying position with or without HTS. The subjects will be instructed to avoid excessive exercise outside of the study participation to avoid over-fatigue from 48 hours before of session 1 until session 2. During session, a physical therapist will be continuously present to provide guidance and ministering in order to ensure that exercise is performed safety and property. Each session will begin with 5 minutes rest and 5 minutes warm-up.

Hybrid Training System

We used an electrical stimulation unit (HIZA TRAINER, EU-JLM50S, Panasonic Corporation, 1006 Ohaza-Kadoma, Kadoma City, Osaka, Japan) including electrodes (Sekisui Plastics Co., Tokyo, Japan) coated with an oxidation-resistant silver-carbon compound placed with low impedance gell over the quadriceps on the anterior thigh, and over the hamstrings on the posterior thigh as shown in our past study[29]. Size of the electrodes was 12 cm × 5cm for the quadriceps and 10 cm × 5 cm for the hamstrings. Electrical stimulation parameters were based on a standard Russian waveform in which a 5,000 Hz carrier frequency is modulated at 40 Hz (2.4 ms on, 22.6 ms off) to deliver a rectangular voltage biphasic pulse. Electrical stimulation intensity was set to approximately 80% of the subject’s maximum tolerance. This intensity has been reported to successfully improve muscle strength and mass without causing pain or numbness [30] [26, 29].

Safety

The electrical stimulation device has a stimulus pattern with interlock and limiter for safety. Therefore, the effective current is interlocked at 11-12 mA (1kΩ), and the peak voltage and current is limited to under 80 V and 90 mA. These specifications meet a safety level of the Japanese Industrial Standards.

Knee bending exercise with the lying position with HTS or without HTS

The knee bending exercise will be conducted with the lying position on the bed using the knee bending device for 15minute including 5 minutes warm-up. The subjects will bend at 60 rpm. The joint range of motion was restricted to a 90º arc that extended from 10º to 100º. During bending exercise, both lower thighs will be stimulated using HTS device in response to the knee bending. A joint motion sensor that triggered stimulation of the antagonist once it sensed the initiation of an agonist’s volitional contraction.

Statistical Methods:

Subject demographics will be described using means ± SD for continuous variables and percentages for categorical variables.

We will use a one-sample t-test to assess changes from the baseline in each group (with/without HTS). We will use repeated measures ANOVA (within factor: time and between groups).

How Future Studies Will Differ:

Future study will be randomized controlled trials, with stricter inclusion and exclusion criteria. Future studies will also have a sufficient sample size to compare quantitative outcome measures between intervention and control participants. Future studies will also have a measurement method that can evaluate a workload more. Future studies will evaluate the effect of long-term intervention.

Description of the enrollment and consent prosecco for participants:

Information about the study will be posted in local events (such as health fairs) and in our associated facilities (such as hospital, clinic, university). If interested in the study, the potential participant will content study staff either in person or by phone to discuss their participation and eligibility for the study. If eligible and interested in the study, the potential participant will be scheduled for an assessment visit. If time permits, directions to the appointment and what to expect at the appointment. At the beginning of the assessment visit, the potential participant will participate in an informed consent process, culminating in signing the informed consent document. A copy of the signed informed consent document will be given to participant.

Questionnaires, surveys, written assessments that will be used to obtain data directly from participants in this study:

・Baseline visit data collection form

・Phone screen

Procedures following the consent process:

Baseline Assessment: (60min)

Participants who have met initial eligibility criteria by the phone screen will attend a 60-minutes baseline visit with the study coordinator to undergo the following:

1. Informed Consent Process: At the beginning of this baseline assessment visit, potential participants will undergo an informed consent process. (30min)
2. Confirm eligibility: 1) Vital Signs: The participant will have blood pressure checked to ensure it is less than 180/100. If not within this range, the participant will not be eligible to take part in the study. 2) Height and body weight: BMI will be calculated from them. (2min)
3. Body composition will be assessed with Inbody (S10): Total leg muscle mass and Skeletal muscle index (SMI) will be calculated. (3min)

The exercise session assessment Visit1 and 2 (30min):

1) Oxygen intake and carbon dioxide output will be measured using an expiration gas analyzer (AE-100i, Minato Medical Science Co. Ltd., Osaka, Japan). Oxygen intake and carbon dioxide output will be measured three patterns (rest with the sitting position (5min), rest with the lying position (5min), and knee bending exercise with HTS or without HTS (10min)).

2) Heart rate will be measured using a by electrocardiogram during the tests.

Compensation for participation in Study

Participants will be provided parking at no cost for appointment.

Participants who are eligible and participate in the study will receive 1,000 yen per time restriction one hour. Payments will be transferred to each account.

What are the risks to participants?

Risks and discomforts include muscle soreness associated with exercise session and psychological stress by on-duty hours. This type of soreness is expected to be transient. Also, there is possible discomfort associated with electrical stimulation. As with any physical activity although exercise load is assumed to be less than 4 Mets, there is a risk of a cardiovascular event, such as abnormal blood pressure, fainting, irregular heartbeat, or cardiac ischemia, and heart look. There is the possibility of unconverging an existing problem during the study. There is also a possibility of contact dermatitis to the surface electrode gel.

What will be done to minimize the risks?

Information will be kept as confidential as possible through the use of code numbers on all forms and in all data analysis programs. No individual analyses will be performed using the data. Paper forms will be stored at the office site in filing cabinets, which are locked, and office is locked outside of work hours.

Risk of cardiovascular events is minimized through the use of screening questions. All exercise training staff are trained in CPR. Risks of muscular discomfort and strains are minimized through the use of warm-ups and cool-downs. Qualified personal, trained in the principles of exercise physiology, will supervise all training sessions, will supervise all exercise sessions, to ensure that proper form and technique is maintained.

All participant information is kept confidential, primarily through restricted access of participant’s data and records. Only the research staff members have access to the study participant’s data and records. Records are kept in a locked office. When participants are randomized, they are assigned a number to identify them in the database. Our research does not involve sensitive matters such as sexual or criminal behavior, and therefore, we have not deemed it necessary to obtain a Certificate of Confidentiality. Participant information will not be disclosed outside of the Kurume University, except when medically necessary, i.e. if a medical condition is discovered during the course of the study, we will disclose that information to the study participant’s primary care physician with his/her permission.

The examinations of muscular performance essentially mimic activities of daily living but are performed on machines. Participants are asked if they may be unable to perform the exercise test, that is recorded, and the examination is not done. Participants will be verbally and visually monitored continuously during the intervention visits and will be provided with rest breaks and water whenever needed. The surface electrodes are hypoallergenic.

References

1. Yanagi T, Shiba N, Maeda T, Iwasa K, Umezu Y, Tagawa Y et al. Agonist contractions against electrically stimulated antagonists. Arch Phys Med Rehabil 2003;84(6):843-8.

2. Iwasaki T, Shiba N, Matsuse H, Nago T, Umezu Y, Tagawa Y et al. Improvement in knee extension strength through training by means of combined electrical stimulation and voluntary muscle contraction. Tohoku J Exp Med 2006;209(1):33-40.

3. Matsuse H, Nago T, Takano Y, Shiba N. Plasma growth hormone is elevated immediately after resistance exercise with electrical stimulation and voluntary muscle contraction. Tohoku J Exp Med 2010;222(1):69-75.

4. Takano Y, Haneda Y, Maeda T, Sakai Y, Matsuse H, Kawaguchi T et al. Increasing muscle strength and mass of thigh in elderly people with the hybrid-training method of electrical stimulation and volitional contraction. Tohoku J Exp Med 2010;221(1):77-85.

5. Ohmoto M MH, Takano Y, Yamada S, Ohshima H, Tagawa Y, Shiba N. Oxygen Uptake during Aerobic Cycling Exercise Simultaneously Combined with Neuromuscular Electrical Stimulation of Antagonists. J Nov Physiother 2013:3-6.

6. Matsuse H, Shiba N, Takano Y, Yamada S, Ohshima H, Tagawa Y. Cycling exercise to resist electrically stimulated antagonist increases oxygen uptake in males: pilot study. J Rehabil Res Dev 2013;50(4):545-54.

7. Hashida R, Takano Y, Matsuse H, Kudo M, Bekki M, Omoto M et al. Electrical Stimulation of the Antagonist Muscle during Cycling Exercise Interval Training Improves Oxygen Uptake and Muscle Strength. J Strength Cond Res 2017.

8. Kawaguchi T, Shiba N, Takano Y, Maeda T, Sata M. Hybrid training of voluntary and electrical muscle contractions decreased fasting blood glucose and serum interleukin-6 levels in elderly people: a pilot study. Appl Physiol Nutr Metab 2011;36(2):276-83.

9. Kawaguchi T, Shiba N, Maeda T, Matsugaki T, Takano Y, Itou M et al. Hybrid training of voluntary and electrical muscle contractions reduces steatosis, insulin resistance, and IL-6 levels in patients with NAFLD: a pilot study. J Gastroenterol 2011;46(6):746-57.

10. Omoto M, Matsuse H, Hashida R, Takano Y, Yamada S, Ohshima H et al. Cycling Exercise with Electrical Stimulation of Antagonist Muscles Increases Plasma Growth Hormone and IL-6. Tohoku J Exp Med 2015;237(3):209-17.

11. 　　木村　浩彰、三上　幸夫、牛尾　会：運動障害以外の疾患に対するエビデンスに基づいた電気刺激療法：Jpn J　Rehabili　Med　2017;54:590-595

12. 　　　見供　翔、武井　仁、石井彩也香、市川和奈：健常者に対する有酸素運動と低周波電気刺激を併用した運動が運動耐容能及び能力に及ぼす影響:仁保学誌　2013;Vol.16 No.2.

13. 　　　Mathes S, Lehnen N, Link T et al. Chronic effects of superimposed electromyostimulation during cycling on aerobic and anaerobic capacity. Eur J Appl Physiol 2017; 117, 881-892.

14. 　　　Carty A, McCormack K, Coughlan GF et al. Increased aerobic fitness after neuromuscular electrical stimulation training in adults with spinal cord injury. Arch Phys Med Rehabil 2012;93, 790-795.

15. 　　　Hasnan N, Mohamad Saadon NS, Hamzaid NA et al. Muscle oxygenation during hybrid arm and functional electrical stimulation-evoked leg cycling after spinal cord injury. Medicine (Baltimore) 2018;97, e12922.

16. 　　　Aaron SE, Vanderwerker CJ, Embry AE et al. FES-assisted Cycling Improves Aerobic Capacity and Locomotor Function Postcerebrovascular Accident. Med Sci Sports Exerc 2018;50, 400-406.

17. 　　　van Buuren F, Horstkotte D, Mellwig KP et al. Electrical Myostimulation (EMS) Improves Glucose Metabolism and Oxygen Uptake in Type 2 Diabetes Mellitus Patients--Results from the EMS Study. Diabetes Technol Ther 2015;17, 413-419.

18. 　　　橋本　健志：運動によるミトコンドリア活性：乳酸の役割：日本抗加齢医学会雑誌2015;Vol.11 No.3.
