# Supplementary material for: Electrically stimulated eccentric contraction during non-weight bearing knee bending exercise in the supine position increases oxygen uptake: A randomized, controlled, exploratory crossover trial
Source: PLoS One. 2021 Nov 18;16(11):e0259856. doi: 10.1371/journal.pone.0259856 (PMC8601547; doi:10.1371/journal.pone.0259856)
Supplement: S1 Data — (PDF) [file pone.0259856.s005.pdf]

― 課 題 名 ―

健常者に対して臥位膝屈伸運動と経皮的電気刺激を併用した時の呼気ガスの変化に関する探索的研究～不動性筋骨格萎縮予防法の開発～

久留米大学病院リハビリテーション部
研究責任者：松瀬　博夫
作成年月日：令和元年８月１日（Ver.1.0）

1. 標題

健常者に対して臥位膝屈伸運動と経皮的電気刺激を併用した時の呼気ガスの変化に関する探索的研究～不動性筋骨格萎縮予防法の開発～

2. 研究の実施体制（研究組織）

研究責任者：久留米大学リハビリテーションセンター　准教授　松瀬　博夫
研究分担者：
久留米大学リハビリテーションセンター　助教　橋田　竜騎
久留米大学リハビリテーションセンター　助教　戸次　将史
久留米大学リハビリテーションセンター　助教　岩永　壮平
久留米大学リハビリテーションセンター　助教　大本　将之
久留米大学リハビリテーションセンター　助教　田島　裕之
久留米大学リハビリテーションセンター　講師　名護　健
久留米大学リハビリテーションセンター　助教　東　恵理子
久留米大学リハビリテーションセンター　教授　志波　直人

安全性評価者：
久留米大学医学部内科学講座消化器内科部門　講師　川口　巧
久留米大学医学部内科学講座心臓・血管内科部門　講師　佐々木　健一郎

3. 研究の背景及び意義

微小重力によるメカニカルストレス(身体に対する機械的な刺激)の減少によって、筋骨格系は廃用性変化をきたし、筋力や骨量が著しく減少する。そのため、その対抗策は宇宙医学の重要課題の一つである。一方で、加齢や治療に伴うベッド臥床などによる活動量と荷重ストレスの減少によって微小重力下の変化と同じように筋力と骨量が減少する。その対策は、臨床医学の重要課題の一つである。そのため、宇宙医学と臨床医学の相互フィードバックの意義はとても大きいものである。

電気刺激療法は、鎮痛を目的とした経皮的電気刺激(TENS; transcutaneous electrical nerve stimulation) と骨格筋の収縮を目的とした機能的電気刺激(FES; functional electrical stimulation)や治療的電気刺激(TES; therapeutic electrical stimulation) がある。リハビリテーション医学では、古くから中枢性麻痺の機能再建に FES が利用され、埋め込み式電極によって高機能的な再建が可能である。FES は、重力に逆らった運動を電気刺激筋収縮によってアシストするのに対し、我々は電気刺激筋収縮が重力に代わって運動に抵抗することでいわゆる抵抗運動が可能となるコンパクトで大がかりな身体固定を必要としない新しい電気刺激療法、Hybrid Training System (HTS) を開発した。(図1) (Matsuse et al., 2006)。

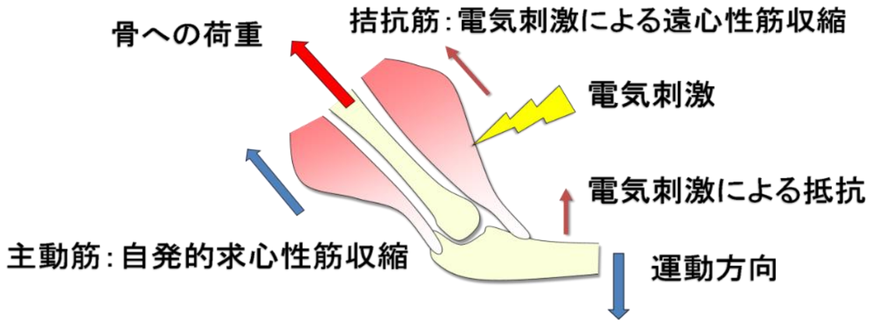

図１：HTS の原理。例：肘関節。屈曲運動時の拮抗筋である上腕三頭筋、伸筋に電気刺激を加えて肘屈曲の運動抵抗とする。伸展運動ではこの逆。電気刺激遠心性収縮は、同一電気刺激による求心性収縮に比べ 30～50%大きな力を発生するため、従来の電気刺激によるトレーニング法に比べると、低い電気刺激の強さを使用可能。さらに、使用者の電気刺激による不快感や疼痛（galvanic pain）を低く抑えることができる。

電気刺激装置は基本的に病院などリハビリテーション施設で使用される低周波治療器と同様であるが、運動時に自動的に反対側の筋肉が刺激されて負荷となる点が従来装置とは異なる。運動時骨には負荷が加わる。このため、長期宇宙滞在における宇宙飛行士に対して、効率よく筋力・筋量、さらに骨密度維持が期待される。小型軽量であるため、将来の月や火星など、小型宇宙船での長期滞在で有用な装置となると考えている(Matsuse et al., 2006)(Shiba et al., 2015)。

一方で、高齢化が進む中, 加齢によって筋力が低下し、日常生活が制限され、高齢者では転倒や引きこもりなどの原因となる。さらに、がんや心血管障害などの内部障害の治療のために安静臥床を余儀なくされ不動症候群を生じ筋骨格系の機能低下はより一層低下する。そのため、それらの予防と治療が高齢者の健康増進や健康寿命の延伸のために欠かせない。その方法で最も安全で効果的なものは運動療法である。有効な運動強度は、中等度以上が推奨されているがベッド上や歩くことが難しくなった高齢者では困難である。そのため、高齢者やベッド上で容易に実施できる両膝屈伸運動が提唱されたが安静座位の2倍程度の運動強度であるため少なくとも日常生活相当の運動負荷が得られる効果的な運動法の開発が望まれ、HTSを用いた臥位での膝屈伸運動はその有用な技術であると考えられる(Matsuse et al., 2006)。

HTS に関しては、これまでに膝を含む様々な関節の屈伸運動に使用し、筋力増強、筋肥大効果を示した(Iwasaki et al., 2006; Matsuse, Nago, Takano, & Shiba, 2010; Takano et al., 2010; Yanagi et al., 2003)。さらに、有酸素運動と組み合わせ運動負荷を容易に増加させる手法としても利用でき(Iwasaki et al., 2006) (Takano et al., 2010) (Takano et al., 2016)、無負荷のエルゴメータに HTS を同時に行うと、酸素摂取量が約 4.4%増加する(Ohmoto M, 2013)。さらに、20 ワットから 100 ワットのエルゴメータに HTS を同時に行うと平均約 21.1%増加する(Matsuse et al., 2013)。このように、有酸素運動に HTS を同時に組み合わせることによって筋力増強効果だけではなく有酸素運動効果もあると考えられる(Hashida et al., 2017)。

運動は、メカニカルストレスによる筋や骨への直接的な効果だけではなく、運動によって血中に分泌されるホルモンやサイトカイン(細胞から分泌されるタンパク質)を介したいわゆる組織連関によって脂質代謝や糖代謝など全身への効果が期待される(図 11)(Kawaguchi, Shiba, Maeda, et al., 2011; Kawaguchi, Shiba, Takano, Maeda, & Sata, 2011)。加えて、電気的筋収縮は嫌気性代謝優位であるという特性から嫌気性代謝産物である乳酸を介し

た全身への効果(Matsuse et al., 2010; Omoto et al., 2015)も期待される。
近年、有酸素運動に加え神経筋電気刺激(NMES; neuromuscular electrical stimulation)を組み合わせて行うことで、有酸素運動のみと比較して最大酸素摂取量の改善や運動耐容能の向上が報告されている(Mathes et al., 2017) (Hashida et al., 2017)。更に、NMESの併用により2型糖尿病患者において、酸素摂取量の向上、糖代謝の改善を認めたと報告され、代謝疾患においても効果的である(van Buuren et al., 2015)。
しかしながら、臥位膝屈伸運動にNMESを同時に実施した場合(臥位HTS膝屈伸運動)の身体に与える影響についての報告は十分ではなく、特にエネルギー代謝に与える影響については宇宙医学だけでなくベッド上でのリハビリテーションに適応させるうえで検討する必要がある。
そこで、本研究で臥位膝屈伸運動におけるHTSの影響を評価する。

4.研究の目的
本研究では健常者に臥位膝屈伸運動と経皮的電気刺激を併用し呼吸・代謝機能の変化量を呼気ガス分析を基に評価することである。

5.研究デザイン
無作為割付2x2クロスオーバーデザイン

6.研究対象者の選定方針
ⅰ) 対象者および募集方法
介入対象:健常成人男女　目標症例数20例
久留米大学病院、久留米大学医療センター、国際医療福祉大学大川校キャンパス内、ブリジストンジム、久留米市内コミュニティセンターなどにてポスターを掲示する。倫理的配慮を十分に考慮し対象者として選択する。
また、本研究は探索的研究であることから必要症例数の算出は行わない。

ⅱ) 選択基準
以下の基準を全て満たす
・同意取得時の満年齢が20歳以上から50歳未満の非喫煙者の男女
・本人から文書による同意が得られた方
ⅲ) 除外基準
以下のような筋骨格や心肺機能に影響を与えるような既往歴
除外基準のいずれかに該当する方は、対象から除外する。
除外規準
1) 重度の心不全、腎不全、呼吸不全、脳血管疾患、その他悪性疾患を有する方
2) 炎症性疾患を有する方
3) 上記以外に身体に異常を感じ運動が困難な方
4) 医師から運動を禁じられている方
5) その他、研究責任医師又は分担医師が対象として不適格と判断した方
6) 原則として久留米大学リハビリテーションスタッフ及び学生
また、研究期間中に定期的なスポーツ活動に参加しないこと

7. 研究対象者の登録および割り付け方法

研究責任者及び研究分担者は、文書にて同意を取得した研究対象者に対して、適格性の確認を行い、適格とされた方を研究対象者として本研究への登録を行う。また、本研究は単群で行う介入研究のため、研究対象者の割り付けは行わない。

8. 研究期間

研究期間：倫理委員会承認後（2020 年 4 月 1 日から 2021 年 8 月 1 日まで

9. 使用する機器

ｉ）研究機器の概要

① 心肺運動負荷試験（CPX）：ミナト社医科学株式会社：mobile aeromonitor AE-100i

呼気ガスを分析する装置。吸気と呼気に含まれるガスを分析することで、生体に取り込まれた酸素量と生体から排出された二酸化炭素量を定量する

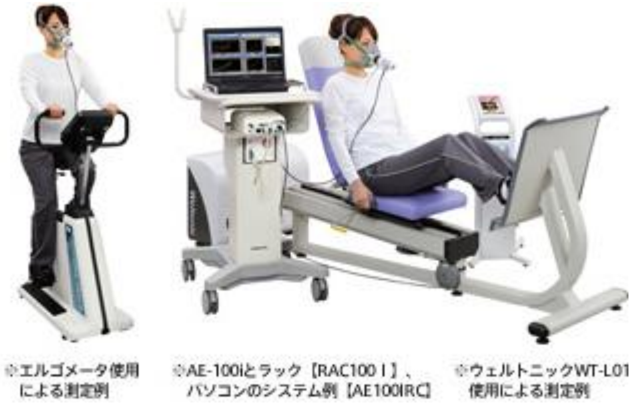

② リスタボード：シグマックス社：571600

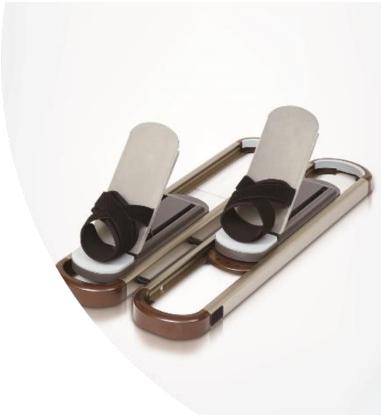

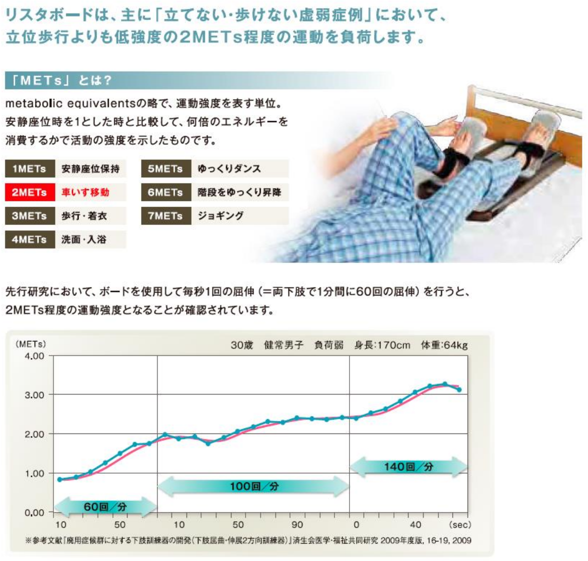

③　電気刺激機能付き膝サポーター：Panasonic社製、EU-JLM52S

（家庭用低周波治療器（クラスⅡ））

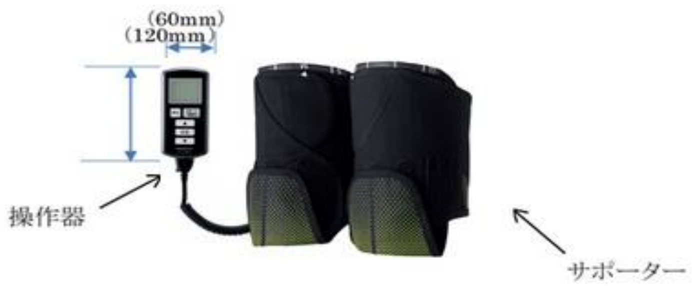

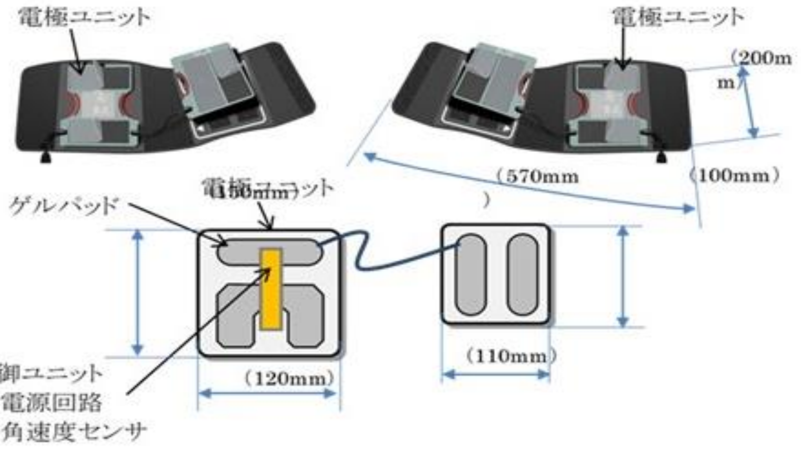

運動風景

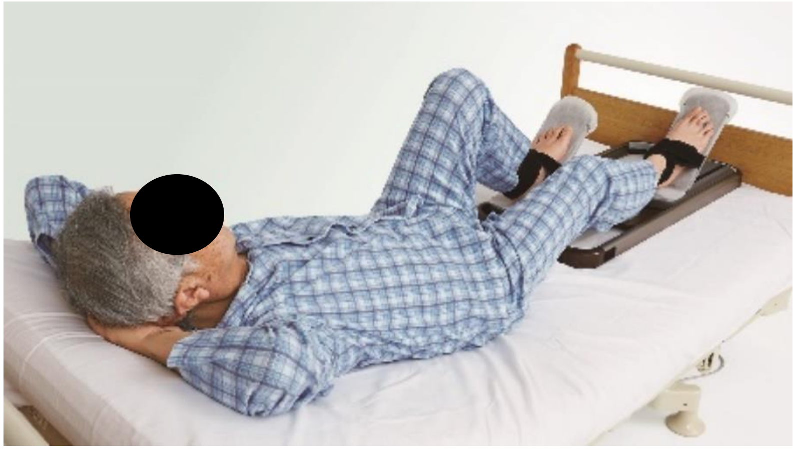

ⅱ）研究機器の使用方法

①　心肺運動負荷試験（CPX)：呼気ガス分析を併用することで、運動耐容能や運動能力

などを客観的に評価でき、また運動負荷を測定できる。
②　リスタボード：臥位リスタボードを使用することで、臥位で重力の影響が少ない状態での運動を模擬した安定した臥位膝屈伸運動が実施でき、運動負荷と運動速度を適切に設定できる。
③　低周波電気刺激（NMES）：骨格筋に電気刺激を加えることで筋収縮を誘発するものであり、筋力増強、神経筋促通を目的とした物理療法手技を行う。

iii）危険性
使用する訓練機器は、障害者や高齢者にけるリハビリテーションに広く使用されている一般的な訓練装置であり負荷も安静座位から日常生活動作レベルと非常に軽いことから特別な危険性はなく、当院でも今まで問題が生じたことはない。

10.研究方法
ⅰ）調査項目・スケジュール
・身体計測（身長、体重、体組成）
・心肺運動負荷試験：最高酸素摂取量、呼吸商、嫌気性代謝閾値など

Visit 0
・研究の目的の説明と同意の取得
・問診

Visit 1　（介入1）
・臥位膝屈伸運動＋HTS＋（A）OR　HTS－（B）
・呼気ガス分析

Visit 2（介入2）
・臥位膝屈伸運動＋HTS＋（A）OR　HTS－（B）
・呼気ガス分析
評価はVisit1と全く同じとする。

Visit1とVisit2のどちらか一方で膝屈伸運動中に電気刺激を同時に行う。その順番は、無作為化する。（下記ABコースまたはBAコース）

短期介入試験スタディーカレンダー

	Visit0	Visit1	Visit2
		Day0	Day7
対象の選定	○		
同意の取得	○		
問診	○		
機器説明	○		
身体計測（身長・体重・体組成）	○		
運動中の呼気ガス分析		○	○
臥位膝屈伸運動（ABコース）		電気刺激+	電気刺激－

臥位膝屈伸運動<br>（BA コース）		電気刺激-	電気刺激+

※AB 又は BA コースをランダムに選択する。

ⅱ）具体的手順

本研究は電気刺激が運動前後での呼吸・代謝機能の変化を調査するために行う。そのために、クロスオーバーデザインで電気刺激の有無の違いを単回介入にて評価を行う。なお、専門である、整形外科医師、リハビリテーション科医師、理学療法士のいずれかが身体機能評価を行う。

介入：臥位膝屈伸運動に電気刺激を加え代謝の変化を心肺運動負荷試験（CPX）を用いて評価する。

介入時間、頻度、期間：10分間、週1回、合計2週間（介入間隔は少なくとも1週間）

運動負荷：電気刺激無しを基準に２Mets相当の運動負荷に設定する。

運動速度：先行研究に合わせ1分間に60回（１Hz）の膝屈伸とする。

訓練場所：久留米大学病院　リハビリテーション室

ⅲ）使用する測定情報等の項目

①　測定情報等	☒年齢　☒性別　☒生年月日　☐住所　☐顔写真
	☒身長・体重
	☒病歴（要配慮個人情報）
	☒既往歴
	☐治療歴（治療薬、投与量、治療開始日等）
	☐血液検査データ【】
	☐画像データ【　　　】
	☐アンケート（通常診療内で実施されたもの）【】
	☒有害事象（副作用・合併症の発生等）
	☒その他【身体機能、体組成などの計測データ】

ⅳ）評価項目

主要評価項目

・運動中酸素摂取量（呼気ガス分析）

副次評価項目

・運動中二酸化炭素排出量（呼気ガス分析）

・運動中心拍数

・呼吸商

ⅴ）解析方法

呼吸・代謝機能に関する測定値は経時的に測定されており、プライマリエンドポイントとして曲線下面積(AUC: area under the curve)を算出し、群間比較を 2x2 クロスオーバーデザインに基づいた線形混合モデル（固定効果：介入、順番、期間、ランダム効果：被検者）を

用い検討する[ref]。

ref Jones, B., M. Kenward. (2015) Design and Analysis of Cross-Over Trials. 3rd Ed. CRC.

解析担当：橋田竜騎

11. 中止基準

ｉ）研究対象者の中止

本研究開始後、研究結果の公表前に研究対象者および代諾者等から本研究への同意撤回があった場合には、直ちに本研究の参加を取りやめる。また、測定中や運動中に過度な疲れや痛みなどによって測定や運動を続けることができなくなるような有害事象の発現により、担当医師が試験の継続を不適当と判断した場合は、直ちに本研究の参加を取りやめる。その際、該当する情報等は本研究には使用しない。

ⅱ）研究全体の中止

以下に該当する場合は、本研究を中止する。

・目標症例数を下回り、研究の完遂が困難と判断した場合

・当該研究以外から得られた関連情報により、研究継続の意義がないと判断された場合

・倫理審査委員会又は研究機関長から本研究を中止するよう通知された場合

12. 研究実施後における医療の提供に関する対応について

研究終了後において、当該研究の結果により得られた最善の医療（予防、診断及び治療）を受けることができるよう対応する。

13. 研究対象者に予測される利益と不利益（危険性）

ｉ）予測される利益（効果）

身体機能に関する計測、評価を受けるため、自身の身体機能や代謝機能についての情報を得ることができる。

ⅱ）予測される不利益（副作用・危険性を含む）

検査前説明に 30 分間、介入及び前後の測定時間は合計 30 分程度と時間的負担が生じ、情報過多に伴う精神的ストレス、運動や計測に伴う疲労感や筋肉痛などが考えられるが、十分に休憩をとる。さらに、介入と介入の間には十分な休息も含め 1 週間以上の間隔をあける。万が一、発症時は医師の診察に基づき、適切な医学的処置にて対処する。

14. 研究対象者の保護

ｉ）倫理原則の遵守

本研究は、ヘルシンキ宣言に基づく倫理的原則を遵守し、「人を対象とする医学系研究に関する倫理指針」に従って実施する。なお本研究を実施するにあたり、久留米大学の倫理委員会にて審査後、研究機関長の承認を得る。

ⅱ）個人情報等の安全管理

研究の実施に関わる者は研究対象者のプライバシー及び個人情報保護に十分配慮する。研究機関の長は研究の実施に際して、保有する個人情報等の保護に必要な体制及び安全管理措置を整備するとともに、研究者等に対して保有する個人情報等の安全管理が図られるよう必要かつ適切な監督を行う。研究で得られた個人データ等を本研究の目的以外で使用する場合は、必要に応じて別途対象者から同意を得る。研究の結果を公表する場合も、個人を特定できる情報は使用しない。

ⅲ）匿名化の方法及び対応表について

研究責任者は個人情報等の保護のために、研究対象者の個人情報とは無関係の研究用 ID に置き換え、どの研究対象者の情報であるかが直ちに判別できないようデータ入力時に匿名化を行う。また、研究責任者は、必要な場合に研究対象者を識別することができるよう対応表を作成する。対応表の管理は、紙媒体又はパスワード付きの USB にて、本研究に一切関与しない者（久留米大学リハビリテーション部秘書塚本貴子）が保管する。なお、対応表の保管期間は研究に係る情報等の保管と同様とする。

15.研究対象者の同意（インフォームド・コンセント）

本研究の実施に先立ち、研究責任者または研究分担者は、研究対象者に別添説明文書を渡し、研究参加の自由、意義、目的、方法、予測される利益・不利益、個人情報の保護等についての十分な説明を行い、研究対象者本人の自由意思にて同意を取得する。さらに、研究参加の有無、一旦同意した後に取り消しについても今後不利益が生じることがないことを説明する。

16.研究対象者の費用負担及び損失補償

ｉ）費用負担

本研究は、整形外科学講座研究費で実施するため、研究対象者の費用負担は一切発生しない。なお、研究対象者に研究のための訪問一回あたり 1000 円のクオカードを渡す。

ⅱ）健康被害に関する損失補償

運動強度は、日常生活で歩く相当を超えるものではないことから健康上問題のないと思われる。万が一、本研究の実施に起因する健康被害が生じた場合は、その治療には原則とし

て研究対象者の健康保険を使用することとし、適切な治療その他必要な措置を受けることができるように対応する。

17. 重篤な有害事象と報告手順

ⅰ）重篤な有害事象の定義

有害事象とは、研究で用いた機材との因果関係がはっきりしないものを含め、研究対象者に生じた全ての好ましくない、あるいは意図しない徴候、症状、または病気のことをいう。その内、以下のもの重篤な有害事象とする。

①死に至るもの

②生命を脅かすもの

③治療のための入院又は入院期間の延長が必要となるもの

④永続的又は顕著な障害・機能不全に陥るもの

⑤子孫に先天異常を来すもの

ⅱ）報告手順

研究責任者または研究分担者は有害事象を認めた時は、直ちに適切な処置を行い、「久留米大学における臨床研究に関連する重篤な有害事象対応手順書」に基づき、速やかに研究機関長に報告を行う。報告の対象となる有害事象は、試験期間中および試験終了後に本研究実施に伴い、関連性が疑われた「重篤な有害事象」について報告を行う。また、その他の有害事象については適切に診療録および症例報告書に記載する。

ⅲ）予測できない重篤な有害事象の定義

重篤な有害事象のうち、添付文書に記載のあるもの以外の事象で予測できない重篤な有害事象とする。

18. 試料・情報（データ）の保管及び廃棄の方法

ⅰ）情報の保管と廃棄の方法

本研究に用いられる情報を久留米大学病院リハビリテーション部医師室（オートロック施錠）内のインターネットに接続されていないPC内に保管する。また、当該情報に係る資料はリハビリテーション部内の施錠可能なキャビネットに保管する。情報の保管期間は本研究の終了報告から5年を超える期間（論文発表後は10年間）とし、測定情報以外の研究対象者から得られた情報は全てシュレッダー処理及びデータの削除を行う。

19. 本研究の資金源、利益相反について

本研究は、整形外科学講座研究費で実施するため、特定企業からの資金援助はないため利益相反は発生しない。

研究責任者及び研究分担者は、久留米大学利益相反マネジメント委員会に関連する企業との利益相反関係について自己申告を行う。
なお、医療機器の有効性・安全性の研究ではない。

20.研究成果（知的財産）の帰属先

本研究で新たな知的財産が生じた場合の権利は久留米大学に帰属する。

21.研究結果の開示、情報公開について

本研究の実施については、大学病院医療情報ネットワーク研究センター（UMIN）の臨床試験登録システムに登録する予定である。本研究での研究成果は、学会での発表及び論文により学術誌への発表を行う予定である。

22.研究機関長への報告内容及び方法

研究責任者は研究の進捗状況を年に 1 回、研究経過報告書を用いて研究機関長へ報告する。また、研究が終了・中止した際には研究終了報告書を提出する。さらに研究計画との重大な逸脱が生じた場合は速やかに報告を行う。

23.データの二次利用について（附随研究について）

本研究で得られた試料・データ（情報）を別の研究に利用する可能性がある。その場合の試料及び情報等は本研究と同様に匿名化し、個人情報の保護を図る。附随研究を行う場合は、改めてその研究計画を倫理委員会において審査し、承認を受けた上で利用する。

24.研究対象者等からの相談の対応

研究責任者及び研究分担者は本研究に係る相談窓口を公開し、研究対象者、代諾者、及びその関係者から受けた質問に対し、研究対象者等の人権や権利保護のため非公開とする内容以外については、速やかに回答し詳細を説明する。また、研究対象者の求めに応じて、他の研究対象者の個人情報などの保護及び当該研究の独創性の確保に支障がない範囲内で、研究計画書及び研究の方法に関する資料の提供を行う。

25.問い合わせ先

久留米大学病院リハビリテーション部　准教授　松瀬　博夫
〒830-0011　福岡県久留米市旭町 67
e-mail:matsuse_hiroh@kurume-u.ac.jp
TEL　0942-35-7568

26.引用文献

Hashida, R., Takano, Y., Matsuse, H., Kudo, M., Bekki, M., Omoto, M., . . . Shiba, N. (2017). Electrical Stimulation of the Antagonist Muscle during Cycling Exercise Interval Training Improves Oxygen Uptake and Muscle Strength. *J Strength Cond Res*. doi:10.1519/JSC.0000000000002393

Iwasaki, T., Shiba, N., Matsuse, H., Nago, T., Umezu, Y., Tagawa, Y., . . . Basford, J. R. (2006). Improvement in knee extension strength through training by means of combined electrical stimulation and voluntary muscle contraction. *Tohoku J Exp Med, 209*(1), 33-40.

Kawaguchi, T., Shiba, N., Maeda, T., Matsugaki, T., Takano, Y., Itou, M., . . . Sata, M. (2011). Hybrid training of voluntary and electrical muscle contractions reduces steatosis, insulin resistance, and IL-6 levels in patients with NAFLD: a pilot study. *J Gastroenterol, 46*(6), 746-757. doi:10.1007/s00535-011-0378-x

Kawaguchi, T., Shiba, N., Takano, Y., Maeda, T., & Sata, M. (2011). Hybrid training of voluntary and electrical muscle contractions decreased fasting blood glucose and serum interleukin-6 levels in elderly people: a pilot study. *Appl Physiol Nutr Metab, 36*(2), 276-283. doi:10.1139/h10-108

Mathes, S., Lehnen, N., Link, T., Bloch, W., Mester, J., & Wahl, P. (2017). Chronic effects of superimposed electromyostimulation during cycling on aerobic and anaerobic capacity. *Eur J Appl Physiol, 117*(5), 881-892. doi:10.1007/s00421-017-3572-6

Matsuse, H., Nago, T., Takano, Y., & Shiba, N. (2010). Plasma growth hormone is elevated immediately after resistance exercise with electrical stimulation and voluntary muscle contraction. *Tohoku J Exp Med, 222*(1), 69-75.

Matsuse, H., Shiba, N., Takano, Y., Yamada, S., Ohshima, H., & Tagawa, Y. (2013). Cycling exercise to resist electrically stimulated antagonist increases oxygen uptake in males: pilot study. *J Rehabil Res Dev, 50*(4), 545-554.

Matsuse, H., Shiba, N., Umezu, Y., Nago, T., Tagawa, Y., Kakuma, T., . . . Basford, J. R. (2006). Muscle training by means of combined electrical stimulation and volitional contraction. *Aviat Space Environ Med, 77*(6), 581-585.

Ohmoto M, M. H., Takano Y, Yamada S, Ohshima H, Tagawa Y, Shiba N. (2013). Oxygen Uptake during Aerobic Cycling Exercise Simultaneously Combined with Neuromuscular Electrical Stimulation of Antagonists. *J Nov Physiother.*, 3-6.

Omoto, M., Matsuse, H., Hashida, R., Takano, Y., Yamada, S., Ohshima, H., . . . Shiba, N. (2015). Cycling Exercise with Electrical Stimulation of Antagonist Muscles Increases Plasma Growth Hormone and IL-6. *Tohoku J Exp Med, 237*(3), 209-217.

doi:10.1620/tjem.237.209

Shiba, N., Matsuse, H., Takano, Y., Yoshimitsu, K., Omoto, M., Hashida, R., . . . Ohshima, H. (2015). Correction: Electrically Stimulated Antagonist Muscle Contraction Increased Muscle Mass and Bone Mineral Density of One Astronaut--Initial Verification on the International Space Station. *PLoS One, 10*(9), e0138519. doi:10.1371/journal.pone.0138519

Takano, Y., Haneda, Y., Maeda, T., Sakai, Y., Matsuse, H., Kawaguchi, T., . . . Shiba, N. (2010). Increasing muscle strength and mass of thigh in elderly people with the hybrid-training method of electrical stimulation and volitional contraction. *Tohoku J Exp Med, 221*(1), 77-85.

Takano, Y., Matsuse, H., Tsukada, Y., Omoto, M., Hashida, R., & Shiba, N. (2016). A New Training for Older Adults Using Combined Neuromuscular Electrical Stimulation and Volitional Contraction: A Pilot Study. *Kurume Med J, 62*(3-4), 73-81. doi:10.2739/kurumemedj.MS66001

van Buuren, F., Horstkotte, D., Mellwig, K. P., Frund, A., Vlachojannis, M., Bogunovic, N., . . . Niebauer, J. (2015). Electrical Myostimulation (EMS) Improves Glucose Metabolism and Oxygen Uptake in Type 2 Diabetes Mellitus Patients--Results from the EMS Study. *Diabetes Technol Ther, 17*(6), 413-419. doi:10.1089/dia.2014.0315

Yanagi, T., Shiba, N., Maeda, T., Iwasa, K., Umezu, Y., Tagawa, Y., . . . Basford, J. R. (2003). Agonist contractions against electrically stimulated antagonists. *Arch Phys Med Rehabil, 84*(6), 843-848.
