# Supplementary material for: Electrically stimulated eccentric contraction during non-weight bearing knee bending exercise in the supine position increases oxygen uptake: A randomized, controlled, exploratory crossover trial
Source: PLoS One. 2021 Nov 18;16(11):e0259856. doi: 10.1371/journal.pone.0259856 (PMC8601547; doi:10.1371/journal.pone.0259856)
Supplement: Attachment — Submitted filename: PONE-D-21-17794_R1 (1).pdf [file pone.0259856.s008.pdf]

PLOS ONE

Electrically stimulated eccentric contraction during non-weight bearing knee bending exercise in the supine position on a bed increases oxygen uptake: a randomized, controlled, exploratory crossover trial

Still the title is not in PICO format --Manuscript Draft-- is it important to write on a bed

**Manuscript Number:**	PONE-D-21-17794R1
**Article Type:**	Clinical Trial
**Full Title:**	Electrically stimulated eccentric contraction during non-weight bearing knee bending exercise in the supine position on a bed increases oxygen uptake: a randomized, controlled, exploratory crossover trial
**Short Title:**	Oxygen uptake during knee bending on a bed short title doesn't match the main title
**Corresponding Author:**	Hiroo Matsuse<br>Kurume University<br>Kurume, JAPAN
**Keywords:**	Analysis of expired gas; Bed rest; Disuse; Metabolic equivalents; Neuromuscular electrical stimulation
**Abstract:**	It is well known that prolonged bed rest induces muscle weakness, muscle atrophy, cardiovascular deconditioning, bone loss, a loss of functional capacity, and the development of insulin resistance. Neuromuscular electrical stimulation is anticipated to be an interventional strategy for disuse due to bed rest. A hybrid training system (HTS), synchronized neuromuscular electrical stimulation for voluntary exercise using an articular motion sensor, may increase the exercise load though bed rest. We assessed oxygen uptake or heart rate during knee bending exercise in the supine position on a bed both simultaneously combined with HTS and without HTS to evaluate exercise intensity on different days in ten healthy subjects (8 men and 2 women) by a randomized controlled crossover trial. The values of relative oxygen uptake during knee bending exercise with HTS were significantly greater than those during knee bending exercise without HTS (7.29 ± 0.91 ml/kg/min vs. 8.29 ± 1.06 ml/kg/min; p = 0.0115). That increment with HTS was a mean of 14.42 ± 13.99%. Metabolic equivalents during knee bending exercise with HTS and without HTS were 2.08 ± 0.26 and 2.39 ± 0.30, respectively. The values of heart rate during knee bending exercise with HTS were significantly greater than those during knee bending exercise without HTS (80.82 ± 9.19 bpm vs. 86.36 ± 5.50 bpm; p = 0.0153). HTS could increase exercise load during knee bending exercise which is easy to implement on a bed. HTS might be a useful technique as a countermeasure against the disuse due to bed rest, for example during acute care or the quarantine for infection prophylaxis.
**Order of Authors:**	Hiroo Matsuse
	Hiroshi Tajima
	Ryuki Hashida
	Takeshi Nago
	Masafumi Bekki
	Sohei Iwanaga
	Eriko Higashi
	Naoto Shiba
**Response to Reviewers:**	PONE-D-21-17794<br>Electrically stimulated eccentric contraction during knee bending exercise in the dorsal position on a bed increases oxygen uptake<br><br>Dear Dr. Matsuse,<br><br>Dear Editorial staff,

Powered by Editorial Manager® and ProduXion Manager® from Aries Systems Corporation

Thank you very much for your exceedingly kind comments for our study. We appreciate it very much. We answered all of your comments and revised the manuscript as follows. We submit the revised manuscript. Please contact me freely if you have any further questions.

Best regards,
Hiroo Matsuse

Journal Requirements:
1. Please ensure that your manuscript meets PLOS ONE's stylerequirements, including those for file naming. The PLOS ONE style templates can be found at https://journals.plos.org/plosone/s/file?id=wjVg/PLOSOne_formatting_sample_main_body.pdf
and
https://journals.plos.org/plosone/s/file?id=ba62/PLOSOne_formatting_sample_title_authors_affiliations.pdf

2. Please amend your Methods section to state what type of informed consent was given (written, verbal, etc.).
A: We added the type of informed consent in the Methods section.
(Line 116)

3. Please amend either the title on the online submission form (via Edit Submission) or the title in the manuscript so that they are identical.
A: We amended both of them according to the reviewer's comments.

4. Your ethics statement should only appear in the Methods section of your manuscript. If your ethics statement is written in any section besides the Methods, please delete it from any other section.
A: We deleted that from the acknowledge section and revised it.

5. Please include captions for your Supporting Information files at the end of your manuscript, and update any in-textcitations to match accordingly. Please see our Supporting Information guidelines for more information:
http://journals.plos.org/plosone/s/supporting-information.
A: We added captions for figures and revised title and legend.

Reviewer #1: Manuscript Number: PONE-D-21-17794
Overall, the idea of research is very interesting. However, there are some comments and suggestions.

Revision Suggestions
Title
I suggest some changes in the title as follow:
- Electrically stimulated eccentric contraction during non-weight bearing knee bending exercise increases oxygen uptake: a randomized controlled crossover trial
A: We changed the title according to your comment. Moreover, we added the word "exploratory" in the title in response to your comment about the setting of sample size as follows: "Electrically stimulated eccentric contraction during non-weight bearing knee bending exercise in the supine position on a bed increases oxygen uptake: a randomized, controlled, exploratory crossover trial"

Abstract
The abstract is better designed as background (a brief one), purpose, methods (participants, study groups, outcome measures), results and conclusion.
A: Thank you for your comment. We added the words "in the supine position" in the abstract to response to reviewer #2's comment.
(Line 46-47)

	Introduction<br>Line 82: please cite the references as (5-7)<br>Line 84: please cite the references as (8,9)<br>A: Thank you for careful confirmation. We rewrote them as directed.<br><br>Materials and methods<br>- Ethical approval and trail registration are addressed.<br>A: We showed them on line 115-116.<br><br>- You need to clarify the study groups and the difference between them<br>A: We added the sentences as follows: “When participants were enrolled, the staff who was not involved in intervention and evaluation randomized the intervention sequence using a random number generator and sealed opaque envelopes with a block size of 5 to allocate to group A (the knee bending exercise test with HTS was performed firstly) or group B (the knee bending exercise test without HTS was performed firstly) (Fig 1). (Fig. 1).”. (Line 120-125)<br><br>- Line 105: correct (SD = 8.5)) to(SD = 8.5)<br>A: We are sorry. We deleted the redundant sign “)”. (Line 118)<br><br>- Sample size was not calculated. Don’t you think the sample of 10 participants is relatively small and the results can't be generalized?<br>A: The sample of 10 participants may not be enough as you pointed out. Because there was not a preliminary study that evaluated the effect that HTS gave to oxygen uptake during knee bending exercise on a bed, we did not set a sample size. We wrote the sentence about this point on Line 226-227. Moreover, we added the expression of the exploratory study to the title to make it clear.<br><br>Also, we added the sentences about the setting of the sample size to the session of the limitation in consultation with a statistician. (Line 357-361)<br>We calculated the more common effect size (r) as a quantity of effect for an absolute number without changing by the sample size for a future study. (Line239-240) We calculated the effect size for t-test which we used to compare groups in this study again in the Result session. (Line262) However, we do not calculate the observed power for the results for an exploratory study. Furthermore, we added results of mean within subject difference (95% confidence interval) to reinforce the results of the primary outcome in this crossover trial. (Line260-262)<br><br>- The validity and reliability of assessment methods not stated<br>A: The validity and reliability analysis of VE and VO2 were performed as a comparison of intraclass correlation coefficients (ICCs) using Case 2 (2,1)<br><br>- For all figures, write the figure label under the corresponding figure.<br>The label should not include statistical results. For example Fig.4. Comparing of changes of oxygen uptake.<br>A: We revised and labeled them all as follows:<br>Fig.3. Comparison of changes in oxygen uptake.<br>Fig.4. Comparison of changes in carbon dioxide production.<br>Fig.5. Comparison of changes in heart rate.<br><br>- No need for fig 2 as figure 3 is sufficient<br>A: We deleted Fig.2 and regulated the number of the figures.<br>Statistical analysis: Satisfying<br>Results: Satisfying<br>Discussion: Satisfying<br>A: Thank you for the confirmation.<br><br>References<br>You need to update some references<br>References<br>Update some references is required<br>A: We confirmed and updated them using the EndNote.<br><br>Reviewer #2:

	1. Title need be revised and make it clear.<br>A: We changed the title according to reviewer 1's advice. Moreover, we added the word "exploratory" in the title to solve reviewer 1's comment about the setting of sample size and changed the words "dorsal" to "supine" according to your next comment as follows: "Electrically stimulated eccentric contraction during non-weight bearing knee bending exercise in the supine position on a bed increases oxygen uptake: a randomized, controlled, exploratory crossover trial"<br><br>2. Can you write supine/prone? It will make easier to reader to read and matches with your manuscript.<br>A: Thank you for your advice. We rewrote it using a word "supine" to make a spine position clear through the whole. (Line 2, 47, 150, 171, 182, 195, 283, 314, 346, 385, 415)<br><br>3. Such as…Can you provide some examples? (line 72)<br>A: We revised that sentence with examples as follows. "Bed rest causes similar disuse(3) although bed rest has been prescribed in the past for several other clinical conditions such as the rest for the intensive care or the quarantine for prevention of infection." （Line71-74）<br><br>4. Would you write the full form of METS in 1st time(Metabolic…) (Line 77)<br>A: Thanks for the input and we added a postscript to full spelling. Also, we deleted the full form from second METs.<br><br>5. Can you provide operational definition? (line 84)<br>A: We revised the sentence to include the operational definition as follows "the combined application of electrical stimulation (ES) and volitional contractions (VC) (performing ES during VC either non-simultaneously or simultaneously) is said to be more effective than ES or VC alone." (Line 86-89)<br>Moreover, we revised the next sentence from the ties with the above sentence as follows. "The hybrid training system (HTS) is one of the techniques that simultaneously combines VC and ES". (Line89-90)<br><br>6. You need strong rational for study. (Line 91)<br>A: Many cases are considered as the clinical situation. However, we revised the sentence to include the explanation of the clinical situation as follows. "Knee bending exercise, one of the common exercises on a bed, is recommended for patients with disuse syndrome in a clinical situation such as the case that cannot walk for the reason of the motor disfunction or the case to be forced to lay for a remedial reason." (Line99-102)<br><br>7. Why did you select healthy participants rather than clinical participants? (line 105)<br>A: This is an exploratory study. Moreover, the subjects of this study were healthy participants as preliminary research before intending for the clinical patients. As you know, we think that a pilot study like this becomes important before intending for the clinical patients. In future, we need the study in clinical patients in discussion. (Line369-370)<br>We think that we have applied kinematical theory in the healthy subject to the clinical patients. We think that we can apply the possibility shown in healthy subjects in future in a larger field.<br><br>8. Inclusion criteria are not clearly specified. Please write them in clear way. Study is in normal population but you have recommended for COVID, Space travelers ????? give strong points (109)<br>A: We revised the inclusion criteria more definitely and rewrote it in detail as follows. "Inclusion criteria were applied: age between 20-50 years, nonsmokers, body mass index less than 30, doing normal physical activity, and normal physical function. A specialist in expert orthopedics and rehabilitation judged the normal physical function from the physical findings such as muscle strength, sensation, and range of motion according to the criteria of the Japanese Orthopedic Association." (Line125-131)<br>Moreover, we revised that expression as follows. "These findings showed the possibility that we might strengthen the metabolic stress from increased physical

Powered by Editorial Manager® and ProduXion Manager® from Aries Systems Corporation

activity by combining HTS with a knee bending exercise that is easily performed by bed ridden patients" (Line352-355)

9. Can you explain why you choose this design of study? line 117
A: The first reason is advice from a statistician of our institution. Generally, we know that this crossover design is suitable for the disease that a case is hard to recruit. However, we think that it is useful to apply this design to the intervention study using the electrical stimulation like this study because we are very difficult to perform this intervention with the complete blindness (both researcher and subject) unlike a study using the placebo medicine. Subjects are on their own controls. The within-patient variation is less than between-patient variation. Therefore, we think that we can evaluate the intervention effect with minimizing the influence of the individual difference by comparing the presence or absence of electrically stimulated muscle contraction during volitional exercise in the same individuals.
We added a postscript to that reason as follows. (Line141-144) "It is very difficult for both subject and researcher to completely blind presence or absence of electrical stimulation. Moreover, we need to minimize the influence of the individual difference. Therefore, we chose a crossover trial in this study."

10. What is RISTA board and it's importance in one sentence? line 120
A: RISTA board is a device to facilitate knee bending exercises in the supine position on a bed. We revised the sentence more clearly and added a postscript to the explanation of the device as follows. "We measured gas exchange while subjects did knee bending exercises with a RISTA board (NIPPON SIGMAX Co, Tokyo, Japan) at the lowest exercise resistance load using the following protocol after a rest for 30 minutes. The RISTA board is a Japanese medical device to facilitate knee bending exercises in the supine position on a bed." (Line145-150)

11. Which techniques, need to be specified? line 126
A: We added a postscript to the technique of the system in detail as follows. "The AE-100i consists of a microcomputer, a hot wire flowmeter, and a gas analyzer, which contains a sampling tube, filter, suction pump, oxygen (O2) analyzer made by a paramagnetic O2 transducer, and an infrared carbon dioxide (CO2) analyzer. Ventilatory parameters were measured using a hot-wire flow meter, and the flow meter was calibrated with a syringe of known volume (2.0l). A zirconium sensor and an infrared absorption analyzer, respectively, measured O2 and CO2 concentrations. The gas analyzer was calibrated to known standard gas levels (O2 15.16%, CO2 5.023%) before each test." (Line154-162)

12. Procedures are not clear.
A: We already described the method of the exhalation gas analysis. (Line151-166)
Moreover, we added the next sentences and revised the first paragraph of the Intervention to make the procedures clearer as follows. "Participants performed the knee bending exercise tests with or without HTS according to a sequence assigned before the exercise test. Next, participants transitioned to the alternative exercise test on different days separated by an interval of one week as a wash out period. Therefore, the participant who first performed the exercise test with HTS performed the exercise test without HTS next." (Line174-179).
Also, we revised the next sentence to make the exercise tests clear as follows. "In the interventional exercise, subjects performed the knee bending exercise with reciprocal 2-second (45degree/sec) knee flexion and extension contractions at the speed of 1Hz using a metronome for 5 minutes with HTS or without HTS in the supine position on a flat surface after warming up at a slow speed for 3 minutes." (Line179-183)
At the same time, we revised the first paragraph of Subjetcs in Method session.

13. Is it Rested or tested? line 133
A: That is "rested". (Line170) We revised the sentence associated with other opinions.

14. Why in sitting position? Why not in supine? line. 134
A: We revised the sentence as follows to make the purpose clear. "At first subjects rested for five minutes in a sitting position to evaluate of the basic inactivity quiet metabolic cost while sitting quietly"(Line172-173)

Powered by Editorial Manager® and ProduXion Manager® from Aries Systems Corporation

It is not our purpose to make the participant rest quietly in bed. We set it as an evaluation of the basic inactivite metabolic cost as 1 MET (sitting quietly). Lying quietly is 1 MET as an inactivite cost also as you mentioned. The Ministry of Health, Labour and Welfare in Japan defines sitting quietly as 1MET; the unit of physical activity or motion. sitting quietly is the first step of a physical activity to achieve an increase from decubitus in a rehabilitation clinic. Therefore, we can estimate the clinical benefit of an intervention by knowing the metabolic cost during the intervention as well as for sitting quietly. Also, we think that we can confirm that intervention in this study is definitely the physical activity from the exercise by comparing it with the conditions measured during the resting state. Because of this, we showed the results of the resting state in figures. Moreover, we added the sentence to distinguish the “rest” before the test definitely as follows. “Participants rested for 30 min in the supine position to minimize the influence of physical activity before the exercise test..” (Line170-172)

15. If the testing was in sitting position than in which position did you performed test? Write it clearly. Intervention is not clear. line137
A: Like the figure 2, we performed the exercise testing (intervention) in the supine position on a bed. In response to your former comment, we added intervention contents to the other places of the paragraph clearly and made modifications as follows. “In the interventional exercise, subjects performed the knee bending exercise with reciprocal 2-second (45degree/sec) knee flexion and extension contractions at the speed of 1Hz using a metronome for 5 minutes with HTS or without HTS in the supine position on a flat surface after warming up at a slow speed for 3 minutes (Fig.2).” (Line179-183)

16. Is this enough period for wash out? 139
A: We think that that was enough. For example, a study which evaluated muscle fatigue from exercise adopts one week as a wash out. (PMID: 33232629、PMID: 25494054) Also, there were only two days as a wash out in a study that evaluated metabolism after aerobic exercise (2km running). (PMID: 28622349) Moreover, the influence of muscle fatigue seems to be restored largely within 3 days. (PMID: 24435468)
Besides, the exercise intensity that we performed in this study is considerably low intensity, equivalent to light activities of daily living. However, there may be muscle damage due to the electrical eccentric contraction although there adverse events including muscle soreness in this study. Also, in another study, we have showed that there was little to no muscle damage after HTS in the arm. (PMID: 17317931) Moreover, the statistical method we used provides a method to test carryover effects. Carryover effects will cause the difference between the two treatments (intervention) to be different in the two time periods. When the interaction is significant, it indicates the presence of carryover. If the carryover effect is not significantly between two sequences. There were no significant differences between sequences in this study. Therefore, we judged that the influence of the exercise intervention in this study did not have any carry over. However, we added a postscript about this matter in the limitation section in Discussion because we might not be able to completely deny it as follows. “Furthermore, the group effect (sequence) was absent, but it is unclear whether we could wash out the influence of HTS in one week. Therefore, a study not implementing a crossover trial may be necessary.” (Line 370-373)

17. You performed the test only one time? Or more than that? line 142
A: We tested once for each intervention (with HTS or without HTS) on different days, respectively, as we wrote in the first half of Intervention section and figure 1. We rewrote it for every test (with HTS or without HTS) to be plain as follows. “Successively, subjects performed 2 minutes cool down. Therefore, they had the total exercise time of 10 minutes in each of two tests in consideration of muscle fatigue” (Line184-186)

18. How did you measured the expired gas? line 149
A: We showed how to measure the expired gas at Line 145-166.
Because it is the measurement that is commonly used widely for clinical medicine as well as clinical studies , we think that it is not so special s hat it is necessary to describe the detailed technique in the main text.
Because we used the automated breath by breath system, we can obtain data (VO2, VCO2, and VE) of the gas for each breath. For analysis, we averaged the data of each subject, respectively, (respiratory frequency is different for every individual) obtained in

Powered by Editorial Manager® and ProduXion Manager® from Aries Systems Corporation

the last 2 minutes of the exercise time of 5 minutes. We revised the method that we averaged a little in detail as follows. “Therefore, we averaged the data of HR and expired gas during the last 2 minutes of the interventional exercise time of 5 minutes of each test and used them for data analysis.” (Line190-192)

19. Can you write about benefits of eccentric stimulation or contraction at introduction part? line 170
A: We added that benefit in Introduction part with new references as follows.
“HTS utilizes electrically stimulated eccentric contractions. Eccentric contractions are important for muscle strengthening(1). Furthermore, electrically stimulated eccentric contractions are recognized as a muscle strengthening technique, because we can obtain bigger muscle torque (exercise load) than with concentric contractions alone(2, 3). It has been reported that HTS can increase both muscle strength and mass even when exercise intensity is relatively low(4-7).” (Line91-98)

1.Dudley GA, Tesch PA, Miller BJ, Buchanan P. Importance of eccentric actions in performance adaptations to resistance training. Aviat Space Environ Med. 1991;62(6):543-50.
2.Seger JY, Thorstensson A. Electrically evoked eccentric and concentric torque-velocity relationships in human knee extensor muscles. Acta Physiol Scand. 2000;169(1):63-9.
3.Pain MT, Young F, Kim J, Forrester SE. The torque-velocity relationship in large human muscles: maximum voluntary versus electrically stimulated behaviour. J Biomech. 2013;46(4):645-50.
4.Matsuse H, Shiba N, Umezu Y, Nago T, Tagawa Y, Kakuma T, et al. Muscle training by means of combined electrical stimulation and volitional contraction. Aviat Space Environ Med. 2006;77(6):581-5.
5.Iwasaki T, Shiba N, Matsuse H, Nago T, Umezu Y, Tagawa Y, et al. Improvement in knee extension strength through training by means of combined electrical stimulation and voluntary muscle contraction. Tohoku J Exp Med. 2006;209(1):33-40.
6.Takano Y, Haneda Y, Maeda T, Sakai Y, Matsuse H, Kawaguchi T, et al. Increasing muscle strength and mass of thigh in elderly people with the hybrid-training method of electrical stimulation and volitional contraction. Tohoku J Exp Med. 2010;221(1):77-85.
7.Rabe KG, Matsuse H, Jackson A, Segal NA. Evaluation of the Combined Application of Neuromuscular Electrical Stimulation and Volitional Contractions on Thigh Muscle Strength, Knee Pain, and Physical Performance in Women at Risk for Knee Osteoarthritis: A Randomized Controlled Trial. PM R. 2018;10(12):1301-10.

20. According to provided figure the test position is in supine. It will be clear for reader to write supine rather than dorsal lying. (figure 2,line 179)
A: We revised the expression in "supine position" at Title to make the test position clear. Also, according to the opinion of the other reviewer, we deleted figure 2 and revised the explanation.

21. Write supine. Use same word throughout the manuscript. line 187
A: Associated with the comment mentioned above, we revised all of it.

22. Use same word through out the study rather then different. Don’t confuse readers. line 256
A: Associated with the comment mentioned above, we rewrote it to “in the supine position”. (Line 2, 47, 150, 171, 182, 195, 283, 314, 346, 385, 415)

23. Correct the space. line 323
A: We corrected it.

24. Is this the limitation of study ? line 328
A: We deleted that sentence.

Reviewer #3: A randomized-controlled crossover clinical trial was conducted which aimed to assess oxygen uptake during deep knee bends but with and without a hybrid training system (HTS). Relative oxygen uptake was significantly higher during the HTS than without.

Powered by Editorial Manager® and ProduXion Manager® from Aries Systems Corporation

	Minor revisions:<br>1-Line 201: Clarify if relative values of VO2, VCO2, and HR are differences between HTS and without. In this case a paired t-test was used for comparison as a post hoc test. Correct?<br>A: Because we had paired quantitative measurements from two groups. We corrected it to paired Student's t-test. (Line237)<br><br>2- Line 198: Indicate the underlying covariance structure used in the linear mixed model and the criteria for selecting it.<br>A: We set subjectID(sequence) as random effects covariance parameters for random effects model like a typical model for crossover trials. We rewrote it in detail. (Line234-236)<br><br>3- The standard statistical terminology for "average" is "mean."<br>A: We changed that word to "mean" at Line263.<br><br>4- Table 1: In addition to the frequencies, provide the percentage male.<br>A: We added the percentage male.<br><br>----<br><br>6. PLOS authors have the option to publish the peer review history of their article (what does this mean?). If published, this will include your full peer review and any attached files.<br><br>If you choose "no", your identity will remain anonymous but your review may still be made public.<br><br>Do you want your identity to be public for this peer review? For information about this choice, including consent withdrawal, please see our Privacy Policy.<br><br>Reviewer<br>#1: No<br><br>Reviewer #2: Yes: 1. Bishnu Dutta Acharya<br>2. Arpita Karki<br><br>Reviewer #3: No<br><br>[NOTE: If reviewer comments were submitted as an attachment file, they will be attached to this email and accessible via the submission ite.<br>Please log into your account, locate the manuscript record, and ch ck<br>for the action link "View Attachments". If this link does not appear, there are no attachment files.]<br><br>While revising your submission, please upload your figure files to the Preflight Analysis and Conversion Engine (PACE) digital diagnostic tool,<br>https://pacev2.apexcovantage.com/. PACE helpsensure that figures meet<br>PLOS requirements. To use PACE, you must first register as a user.<br>Registration is free. Then, login and navigate to the UPLOAD tab, where<br>you will find detailed instructions on how to use the tool. If you encounter any issues or have any questions when using PACE, please email PLOS at figures@plos.org.<br>Please note that Supporting Information files<br>do not need this step.<br><br>In compliance with data protection regulations, you may request that we<br>remove your personal registration details at any time. (Remove my<br>information/details). Please contact the publication office if you have<br>any questions.
**Additional Information:**	

Powered by Editorial Manager® and ProduXion Manager® from Aries Systems Corporation

Question	Response
**Financial Disclosure**<br><br>Enter a financial disclosure statement that describes the sources of funding for the work included in this submission. Review the submission guidelines for detailed requirements. View published research articles from *PLOS ONE* for specific examples.<br><br>This statement is required for submission and **will appear in the published article** if the submission is accepted. Please make sure it is accurate.<br><br>**Unfunded studies**<br>Enter: *The author(s) received no specific funding for this work.*<br><br>**Funded studies**<br>Enter a statement with the following details:<br>• Initials of the authors who received each award<br>• Grant numbers awarded to each author<br>• The full name of each funder<br>• URL of each funder website<br>• Did the sponsors or funders play any role in the study design, data collection and analysis, decision to publish, or preparation of the manuscript?<br>• **NO** - Include this sentence at the end of your statement: *The funders had no role in study design, data collection and analysis, decision to publish, or preparation of the manuscript.*<br>• **YES** - Specify the role(s) played.<br><br>* typeset	The authors received no specific funding for this work.
**Competing Interests**<br><br>Use the instructions below to enter a competing interest statement for this submission. On behalf of all authors, disclose any competing interests that could be perceived to bias this work—acknowledging all financial support and any other relevant financial or non-financial competing interests.	The authors have declared that no competing interests exist.

Powered by Editorial Manager® and ProduXion Manager® from Aries Systems Corporation

This statement is required for submission and **will appear in the published article** if the submission is accepted. Please make sure it is accurate and that any funding sources listed in your Funding Information later in the submission form are also declared in your Financial Disclosure statement.<br><br>View published research articles from *PLOS ONE* for specific examples.<br><br>**NO authors have competing interests**<br><br>Enter: *The authors have declared that no competing interests exist.*<br><br>**Authors with competing interests**<br><br>Enter competing interest details beginning with this statement:<br><br>*I have read the journal's policy and the authors of this manuscript have the following competing interests: [insert competing interests here]*<br><br>* typeset	
**Ethics Statement**<br><br>Enter an ethics statement for this submission. This statement is required if the study involved:<br><br>• Human participants<br>• Human specimens or tissue<br>• Vertebrate animals or cephalopods<br>• Vertebrate embryos or tissues<br>• Field research<br><br>Write "N/A" if the submission does not require an ethics statement.<br><br>General guidance is provided below. Consult the submission guidelines for detailed instructions. **Make sure that all information entered here is included in the Methods section of the manuscript.**	This study protocol was approved by the Ethics Committee of Kurume University (No.19209).

Powered by Editorial Manager® and ProduXion Manager® from Aries Systems Corporation

**Format for specific study types**<br><br>**Human Subject Research (involving human participants and/or tissue)**<br>• Give the name of the institutional review board or ethics committee that approved the study<br>• Include the approval number and/or a statement indicating approval of this research<br>• Indicate the form of consent obtained (written/oral) or the reason that consent was not obtained (e.g. the data were analyzed anonymously)<br><br>**Animal Research (involving vertebrate animals, embryos or tissues)**<br>• Provide the name of the Institutional Animal Care and Use Committee (IACUC) or other relevant ethics board that reviewed the study protocol, and indicate whether they approved this research or granted a formal waiver of ethical approval<br>• Include an approval number if one was obtained<br>• If the study involved *non-human primates*, add *additional details* about animal welfare and steps taken to ameliorate suffering<br>• If anesthesia, euthanasia, or any kind of animal sacrifice is part of the study, include briefly which substances and/or methods were applied<br><br>**Field Research**<br><br>Include the following details if this study involves the collection of plant, animal, or other materials from a natural setting:<br>• Field permit number<br>• Name of the institution or relevant body that granted permission	
**Data Availability**<br><br>Authors are required to make all data underlying the findings described fully available, without restriction, and from the time of publication. PLOS allows rare exceptions to address legal and ethical concerns. See the PLOS Data Policy and FAQ for detailed information.	Yes - all data are fully available without restriction

Powered by Editorial Manager® and ProduXion Manager® from Aries Systems Corporation

A Data Availability Statement describing where the data can be found is required at submission. Your answers to this question constitute the Data Availability Statement and **will be published in the article**, if accepted.<br><br>**Important:** Stating 'data available on request from the author' is not sufficient. If your data are only available upon request, select 'No' for the first question and explain your exceptional situation in the text box.<br><br>Do the authors confirm that all data underlying the findings described in their manuscript are fully available without restriction?	
**Describe where the data may be found in full sentences. If you are copying our sample text, replace any instances of XXX with the appropriate details.**<br><br>• If the data are **held or will be held in a public repository**, include URLs, accession numbers or DOIs. If this information will only be available after acceptance, indicate this by ticking the box below. For example: *All XXX files are available from the XXX database (accession number(s) XXX, XXX.*).<br>• If the data are all contained **within the manuscript and/or Supporting Information files**, enter the following: *All relevant data are within the manuscript and its Supporting Information files.*<br>• If neither of these applies but you are able to provide **details of access elsewhere**, with or without limitations, please do so. For example:<br><br>*Data cannot be shared publicly because of [XXX]. Data are available from the XXX Institutional Data Access / Ethics Committee (contact via XXX) for researchers who meet the criteria for access to confidential data.*<br><br>*The data underlying the results presented in the study are available*	https://upload.umin.ac.jp/cgi-bin/ctr/ctr_view_reg.cgi?recptno=R000044862

from (include the name of the third party and contact information or URL).<br>• This text is appropriate if the data are owned by a third party and authors do not have permission to share the data.<br><br>* typeset	
Additional data availability information:	Tick here if the URLs/accession numbers/DOIs will be available only after acceptance of the manuscript for publication so that we can ensure their inclusion before publication.

Powered by Editorial Manager® and ProduXion Manager® from Aries Systems Corporation

Manuscript Click here to access/download;Manuscript;Manuscript.docx

Electrically stimulated eccentric contraction during non-weight bearing knee bending exercise in the supine position on a bed increases oxygen uptake: a randomized, controlled, exploratory crossover trial

[1]Hiroshi Tajima, M.D., [1]Hiroo Matsuse, M.D., Ph.D., [1]Ryuki Hashida, M.D., Ph.D., [1]Takeshi Nago, M.D., Ph.D., [1]Masafumi Bekki, M.D., Ph.D.,[1]Sohei Iwanaga, M.D., Ph.D., [1]Eriko Higashi, M.D., [1]Naoto Shiba, M.D., Ph.D.

1 Rehabilitation center, Kurume University, 67 Asahi-machi, Kurume, Fukuoka 830-0011, JAPAN

Address for corresponding with the author: Hiroo Matsuse, M.D., Ph.D. Rehabilitation center, Kurume University, 67 Asahi-machi, Kurume, Fukuoka 830-0011, Japan. Tel: +81-(0)942-31-7568, Fax: +81-(0)942-46-6002

E-mail: matsuse_hiroh@kurume-u.ac.jp

Running title: Oxygen uptake during knee bending on a bed

Short title doesn't match the main title. It will be good if you include the word electrical stimulation

Author contributions:

1. Hiroyuki Tajima wrote the manuscript and mainly carried out all the experiments.
2. Naoto Shiba and Hiroo Matsuse managed the exercise method combined with hybrid training system.
3. Masafumi Bekki, Sohei Iwanaga, and Eriko Higashi measured and calculated the data.
4. Takeshi Nago and Ryuki Hashida managed this experiment.

Disclosures:

This research did not receive any specific grant from funding agencies in the public, commercial, or not-for-profit sectors. All authors declare that they have no competing interests in this study.

Abbreviation's list:

ES, electrical stimulation; HTS, hybrid training system; HR; heart rate; NMES, neuromuscular electrical stimulation; METs, Metabolic equivalents; RER, respiratory exchange ratio; VC, volitional contraction; VCO2, carbon

dioxide output; VO2, oxygen uptake

Abstract

It is well known that prolonged bed rest induces muscle weakness, muscle atrophy, cardiovascular deconditioning, bone loss, a loss of functional capacity, and the development of insulin resistance. Neuromuscular electrical stimulation is anticipated to be an interventional strategy for disuse due to bed rest. A hybrid training system (HTS), synchronized neuromuscular electrical stimulation for voluntary exercise using an articular motion sensor, may increase the exercise load though bed rest. We assessed oxygen uptake or heart rate during knee bending exercise in the supine position on a bed both simultaneously combined with HTS and without HTS to evaluate exercise intensity on different days in ten healthy subjects (8 men and 2 women) by a randomized controlled crossover trial. The values of relative oxygen uptake during knee bending exercise with HTS were significantly greater than those during knee bending exercise without HTS (7.29 ± 0.91 ml/kg/min vs. 8.29 ± 1.06 ml/kg/min; $p = 0.0115$). That increment with HTS was a mean of 14.42 ± 13.99%. Metabolic equivalents during knee bending exercise with HTS and without HTS were 2.08 ± 0.26 and 2.39 ± 0.30, respectively. The values of heart rate during

knee bending exercise with HTS were significantly greater than those during knee bending exercise without HTS (80.82 ± 9.19 bpm vs. 86.36 ± 5.50 bpm; $p = 0.0153$). HTS could increase exercise load during knee bending exercise which is easy to implement on a bed. HTS might be a useful technique as a countermeasure against the disuse due to bed rest, for example during acute care or the quarantine for infection prophylaxis.

Key words: Analysis of expired gas, Bed rest, Disuse, Metabolic equivalents, Neuromuscular electrical stimulation

Introduction

It is well known that muscle atrophy, cardiovascular deconditioning, bone loss, a loss of functional capacity, and the development of insulin resistance due to disuse occurs in astronauts [1, 2]. Bed rest causes similar disuse[3] although bed rest has been prescribed in the past for several other clinical conditions such as the rest for the intensive care or the quarantine for prevention of infection. Strength training or aerobic exercise programs are expected to prevent disuse atrophy and deconditioning [4]. However, the evidence regarding interventions involving either strength training alone or aerobic exercise alone remains uncertain [4]. Moreover, some patients in acute care hospitals have difficulty even standing. Some patients aren't able to do exercise at intensity 3 Metabolic equivalents (METs) a such as walking. We need relatively efficient and simple exercise that can be performed on a bed to prevent disuse during bed rest.

Neuromuscular electrical stimulation (NMES) is widely used to lessen the complications of disuse during spaceflight or bed rest by increasing muscle strength and mass[5-7]. Moreover, it is well known that

NMES upregulates glucose metabolism and peak oxygen uptake[8, 9]. In addition, the combined application of electrical stimulation (ES) and volitional contractions (VC) (performing ES during VC either non-simultaneously or simultaneously) is said to be more effective than ES or VC alone [10]. The hybrid training system (HTS) is one of the techniques that simultaneously combines VC and ES, and is able to be used during exercises such as ergometer walking. HTS utilizes electrically stimulated eccentric contractions. Eccentric contractions are important for muscle strengthening[11]. Furthermore, electrically stimulated eccentric contractions are recognized as a muscle strengthening technique, because we can obtain bigger muscle torque (exercise load) than with concentric contractions alone[12, 13]. It has been reported that HTS can increase both muscle strength and mass even when exercise intensity is relatively low[14-17]. Moreover, we have reported that ergometer exercise using HTS leads to a greater increase in metabolic cost [18, 19]. Knee bending exercise, one of the common exercises on a bed, is recommended for patients with disuse syndrome in a clinical situation such as the case that cannot walk for the reason of the motor disfunction or the case to be forced to lay for a remedial

reference to support this line 100-102 ?

reason. Therefore, we hypothesize that by combining HTS with knee bending exercise may provide exercise intensity that can easily be performed on a bed. It may be a useful exercise technique for the prevention of disuse due to bed rest.

The purpose of the present study is to compare the metabolic cost between knee bending exercise on a bed with and without HTS by analyzing expired gas.

Methods

Subjects

This study protocol was approved by the Ethics Committee of Kurume University (No.19209) and registered at UMIN Clinical Trials Registry (UMIN000039336). Following approval, written informed consent was obtained from 10 healthy (8 men and 2 women, age 28.9 years (SD = 8.5) who had reviewed the goals of the study and agreed to participate. The subjects of this study were healthy participants as preliminary research before intending for the clinical patients. When participants were enrolled,

the staff who was not involved in intervention and evaluation randomized the intervention sequence using a random number generator and sealed opaque envelopes with a block size of 5 to allocate to group A (the knee bending exercise test with HTS was performed firstly) or group B (the knee bending exercise test without HTS was performed firstly) (Fig 1). Inclusion criteria were applied: age between 20-50 years, nonsmokers, body mass index less than 30, doing normal physical activity, and normal physical function. A specialist in expert orthopedics and rehabilitation judged the normal physical function from the physical findings such as muscle strength, sensation, and range of motion according to the criteria of the Japanese Orthopedic Association. Exclusion criteria were that the subjects had: a musculoskeletal problem, adverse medical history affecting cardiorespiratory function, or some kind of medical treatment. We set these criteria in order to minimize possible confounding effects of subject characteristics over the exercise tests in this study. They were also not to participate in any regular sports activities during the study period.

Design

We performed two cardiopulmonary exercise tests in the clinical laboratory of the rehabilitation center by a randomized, controlled, crossover trial (Fig 1). It is very difficult for both subject and researcher to completely blind presence or absence of electrical stimulation. Moreover, we need to minimize the influence of the individual difference. Therefore, we chose a crossover trial in this study. Environmental conditions were similar for all exercise tests (21 to 24 degrees centigrade, 45 to 55 % relative humidity). We measured gas exchange while subjects did knee bending exercises with a RISTA board (NIPPON SIGMAX Co, Tokyo, Japan) at the lowest exercise resistance load using the following protocol after a rest for 30 minutes. The RISTA board is a Japanese medical device to facilitate knee bending exercises in the supine position on a bed. We used the device to achieve a stable knee bending exercise on a flat surface. During the tests, gas exchange data was collected continuously with an automated breath by breath system (AE-100i, Minato Medical Science Co. Ltd., Osaka, Japan) using the standard technique. The AE-100i consists of a microcomputer, a hot wire flowmeter, and a gas analyzer, which contains a sampling tube, filter, suction pump, oxygen (O2) analyzer made by a paramagnetic O_2

transducer, and an infrared carbon dioxide (CO_2) analyzer. Ventilatory parameters were measured using a hot-wire flow meter, and the flow meter was calibrated with a syringe of known volume (2.0l). A zirconium sensor and an infrared absorption analyzer, respectively, measured O_2 and CO_2 concentrations. The gas analyzer was calibrated to known standard gas levels (O_2 15.16%, CO_2 5.023%) before each test. The intraclass correlation coefficients using Case 2 (2,1) for VO_2 of the primary outcome was 0.876 in our clinical laboratory. Also, heart rates (HR, beats/min) were continuously monitored by electrocardiogram during the tests. O_2 and CO_2 were calculated and recorded during the following exercise tests.

Intervention

The knee bending exercise test was performed as two tests (with HTS or without HTS) for analysis of expired gas. Participants rested for 30 min in the supine position to minimize the influence of physical activity before the exercise test. At first subjects rested for five minutes in a sitting position to evaluate of the basic inactivity quiet metabolic cost while sitting quietly. Then, participants performed the knee bending exercise tests with or

without HTS according to a sequence assigned before the exercise test. Next, participants transitioned to the alternative exercise test on different days separated by an interval of one week as a wash out period. Therefore, the participant who first performed the exercise test with HTS performed the exercise test without HTS next. In the interventional exercise, subjects performed the knee bending exercise with reciprocal 2-second (45degree/sec) knee flexion and extension contractions at the speed of 1Hz using a metronome for 5 minutes with HTS or without HTS in the supine position on a flat surface after warming up at a slow speed for 3 minutes (Fig 2). Successively, subjects performed 2 minutes cool down. Therefore, they had the total exercise time of 10 minutes in each of two tests in consideration of muscle fatigue. The knee joint range of motion was set at a nearly 90° arc that extended from 20° to 110° (0° indicating full knee extension). At the onset of constant-load exercise, O_2 increases in healthy individual's mono-exponentially with a time constant to achieve a steady state below the lactate threshold within about 3 minutes [20]. Therefore, we averaged the data of HR and expired gas during the last 2 minutes of the interventional exercise time of 5 minutes of each test and used them for data analysis.

HTS protocol

HTS was combined with knee bending exercise in the supine position on a flat surface with an electrical stimulator (HIZA TRAINER, EU-JLM50S, Panasonic Corporation, 1006 Ohaza-Kadoma, Kadoma City, Osaka, Japan). This provided a constant voltage stimulus to the skin electrodes (regulated voltage)". This stimulator is household medical equipment including a wrapping band to fix the device and acceleration sensors or electrodes during exercise. The acceleration sensors act as joint motion sensors (EWTS9PD, Home Appliances Development Center Corporate Engineering Division, Appliances Company Panasonic Corporation 2-3-1-2 Noji-higashi, Kusatsu City, Shiga, Japan) and are placed on the front of each leg 88 mm above the patellar edge. The sensors analyze the algorithm of each gait pattern, and stimulate the flexor or extensor in accordance with the motion of each bilateral knee joint during knee bending exercise (Fig 2). Electrodes (Sekisui Plastics Co., Tokyo, Japan) coated with an oxidation-resistant silver-carbon compound and were placed over the quadriceps on the anterior thigh, and over the hamstrings on the posterior thigh with low impedance

gel. The size of the electrodes was 15cm × 5cm for the quadriceps and 10cm × 6cm for the hamstrings. ES stimulated the quadriceps during knee flexion. Conversely, ES stimulated the hamstrings during knee extension. Therefore, the electrically stimulated muscles were eccentric contractions. ES parameters were based on a standard Russian waveform in which a 5,000 Hz carrier frequency is modulated at 40 Hz (2.4 ms on, 22.6 ms off) to deliver a rectangular voltage biphasic pulse. ES intensity was set to approximately 80% of the subject's maximum tolerance. This intensity has been reported to successfully improve muscle strength and mass without causing pain or numbness [16, 17, 21].

Fig 2. Exercise scene.

Statistical Analysis

All variables are presented as means ± SD. This study was not set to the sample size for an exploratory study. The original study plan recruited 20 participants, but we analyzed this study because we were able to achieve

this study purpose from ten participants. We compared the participants' characteristics and rest values (relative VO2, VCO2, and HR) between groups (two sequences) by Student's t-test, or Fisher's exact test. Values for relative VO2, VCO2, HR, and respiratory exchange ratio (RER) during exercise test were analyzed with linear mixed model that included fixed effects of treatment (with HTS or without HTS), period, and sequence. Then, we set subjects(sequence) as the covariance parameter for the random effect. Next, values for relative VO2, VCO2, and HR were assessed using a paired Student's t-test as a post hoc test in order to compare the differences between the knee bending exercise tests with and without HTS for the main purpose of this study. Moreover, we calculated the effect size (r) to know the strength of association between intervention and change on VO2 of the primary outcome. All the statistical analyses were performed using JMP Version 14.0 statistical software (SAS Institute Inc., Cary, NC, USA) and p values < 0.05 were considered to be statistically significant.

Results

There were not any problems and adverse events in the exercise tests for subjects such as not being able to continue from pain or fatigue. Each five subjects participated in two sequences to be shown in the flow diagram (Fig 1), and all ten subjects finished all tests and were analyzed.

There were no significant differences at participants' characteristics and rest values (relative VO2, VCO2, and HR) between groups (Table1). There were no significant differences at period and sequence in values for relative VO2, VCO2, and HR during exercise test though there was significant difference at treatment (with HTS or without HTS). As a primary outcome, the values of relative VO2 during knee bending exercise with HTS were significantly greater than those during knee bending exercise without HTS (7.29 ± 0.91 ml/kg/min vs 8.29 ± 1.06 ml/kg/min; $p = 0.0115$) (Fig 3). Mean within subject difference (95% confidence interval) in the values of relative VO2 between treatments (with HTS and without HTS) was 0.998 (0.3170-1.6785). The effect size was 0.76. The increment using HTS was a mean of 14.42 ± 13.99%. Metabolic equivalents during knee bending exercise with HTS and without HTS were 2.08 ± 0.26 and 2.39 ± 0.30, respectively.

The values of relative VCO2 during knee bending exercise with HTS were significantly greater than those during knee bending exercise without HTS (7.29 ± 0.91 ml/kg/min vs 8.29 ± 1.06 ml/kg/min; $p = 0.0423$) (Fig 4). There was not a significant difference between the values of RER during knee bending exercise with HTS and without HTS (0.94 ± 0.08 vs 0.94 ± 0.10; $p = 0.86$). The values of HR during knee bending exercise with HTS were significantly greater than without HTS (80.82 ± 9.19 bpm vs 86.36 ± 5.50 bpm; $p = 0.0153$) (Fig 5).

Fig 3. Comparing of changes of oxygen uptake.

Fig4. Comparing of changes of carbon dioxide production.

Fig 5. Comparing of changes of heart rate.

Discussion

This is the first study of the metabolic cost of knee bending exercise

combined with NMES that is easily done in the supine position. This study showed that HTS, utilizing electrically stimulated eccentric muscle contractions as a resistance to voluntary exercise, increased exercise intensity during knee bending exercise on a bed. HTS may be a useful exercise technique to prevent disuse during bed rest by providing augmentation of local mechanical stress and systemic metabolic stress.

The decrement in mechanical stress caused by bed rest or spaceflight causes muscle weakness, muscular atrophy, bone atrophy, and the loss of cardiovascular capacity. Resistance exercise is an effective countermeasure for the prevention of muscle atrophy [2] even if it is relatively low volume of resistance exercise [22, 23]. However, Bryan. R. Oates et al. [22] used an exercise intensity of 80% of maximal with resistance exercise. It is difficult for clinical patients to achieve adequate exercise intensity on a bed. The flywheel ergometer has been used for resistance training in space flight. The resistance was provided independent of gravity by using the inertial focus of a flywheel [24]. B. A. Alkner and P. A. Tesch [23] reported the utility of the flywheel ergometer on the bed. However, it is difficult to use a flywheel ergometer for clinical applications

because it is a special device. NMES is widely used in clinic settings and can easily generate sufficient muscle contractions without relying on gravity. Therefore, NMES could be useful as a countermeasure against disuse due to bed rest. It could be especially useful when effective voluntary exercise is not possible due to disturbance of consciousness or strict medical management [25]. However, VC is undoubtedly the most basic effective countermeasure for disuse. In this study, the knee bending exercise which was easily performed in the supine position on a flat surface was approximately 2 METs in exercise intensity. Furthermore, we can make up for the detriments and use them to advantage by combining VC with ES [18, 19, 26, 27]. Moreover, ES should ideally be applied synchronously with voluntary exercise to obtain synergistic benefits [2]. In this study, we could increase exercise intensity by approximately 14.4% of the by combining ES with the knee bending exercise in the supine position on a flat surface. Therefore, we could strengthen the exercise intensity even though in the supine position on a flat surface by using HTS more effectively.

In addition, aerobic exercise is an effective countermeasure against the loss of cardiovascular capacity associated with disuse [2]. Generally, the

metabolic rate during complete bed rest is less than 1 MET. Physical activity is undoubtedly the most effective countermeasure against disuse. The knee bending exercise in this study resulted in approximately 2 METs of metabolic rate. NMES stimulates metabolic response [28] and energy consumption, carbohydrate oxidation, and whole-body glucose uptake [9]. Therefore, we may use NMES as aerobic exercise to improve physical capacity [29]. However, NMES did not change maximum capacity although aerobic exercise did in rats [30]. Additionally, NMES did not improve

Is it safe to use fNMES for cardiac pateints?

functional capacity in cardiac valve surgery patients in the immediate postoperative period [31]. Therefore, the aerobic exercise effect of NMES as a countermeasure against disuse due to bed rest may be limited. ES activation is considered nonselective with regard to the type of motor unit and synchrony, and it preferentially activates Type II fibers as compared with VC [32]. Jean Theurel et al. showed that the O2 and RER were significantly higher during ES compared with VC to compare respiratory gas exchange variables between equal-intensity (i.e., same force output) ES and VC of the quadriceps [33]. They considered that these findings probably reflect differences in the patterns of motor unit recruitment between ES and

VC. On the other hand, in this study, there was not a significant difference between the values of RER between HTS and VC although O2 during HTS was significantly greater than for VC (without HTS). Combining VC with ES simultaneously seems to have been responsible for those results. Similarly, in a past study, we showed that HTS causes an increase of approximately 4.4% of O2 during unloaded cycling without a significant difference in RER [18]. Moreover, HTS caused an increase of approximately 21.1% of O2 during aerobic cycle ergometer exercise at moderate-intensity [19]. In this study, HTS caused an increase of approximately 14.4% of O2 during knee bending exercise in the supine position on a flat surface. In addition, the METs for these studies were approximately 2.39, which is equivalent to light effort such as housework or light exercise. Moreover, a utility of NMES was shown in disuse prevention or the complications prevention in the acute care [34]. Furthermore, NMES is expected as one of the effective exercise therapies during the quarantine for COVID-19 patients [35]. These findings showed the possibility that we might strengthen the metabolic stress from increased physical activity by combining HTS with a knee bending exercise that is easily performed by bed

ridden patients (e.g., rest during the acute care or the quarantine for prevention of infection spread).

There are a few potential limitations of this study. Because this study is an exploratory study, the sample size was not set. At a significance level of 0.05, an effect size in the VO2 variable of 0.76, and a power of 0.80, to detect an inter-group difference in means of 0.998. Nm would require a minimum of 58 subjects for a two-treatment parallel-design study. The purpose of this study was to evaluate the basic gas exchange response to HTS combined with knee bending exercise as a technique to provide physical activity as a countermeasure against disuse due to bed rest. For estimating exercise intensity, oxygen uptake reserve or heart rate reserve is preferable [36]. However, we did not evaluate peak oxygen consumption or peak heart rate in each subject. In addition, we did not evaluate lactic acid or glucose to evaluate the metabolic response after having done strict nutritional management. For the investigation of clinical response, it is necessary to include clinical patients. Furthermore, the group effect (sequence) was absent, but it is unclear whether we could wash out the influence of HTS in one week. Therefore, a study not implementing a crossover trial may be

necessary. Additionally, muscle contraction quantity and time influence kinetic oxygen uptake. Therefore, various protocols of electrical stimulation (such as modification of frequency, pulse width, or duty cycle) will be necessary to evaluate the effect of HTS in a future study. Of course, we should perform a randomized, controlled trial design, long-term investigation study to investigate the efficacy of strength and cardiovascular capacity of HTS for patients with disuse.

Conclusion

These findings indicate that the combined application of VC and EC could lead to a greater increase in metabolic cost during knee bending exercise in the supine position on a flat surface. Knee bending exercise simultaneously combined with HTS might be useful as a countermeasure against disuse due to bed rest by enforcing metabolic stress as well as mechanical stress.

Table 1. Participants' characteristics.

Characteristics	Group A	Group B	p-value
**Age (years)**	27.6 ± 7.3	30.2 ± 10.3	0.6580
Sex (male/female)	4/1	4/1	1.00
Body mass (kg)	66.1 ± 8.6	59.8 ± 10.2	0.3244
Height (cm)	167.8 ± 5.9	168.4 ± 13.3	0.9335
BMI (kg/m^2)	23.4 ± 1.6	21.1 ± 3.1	0.1861
Rest VO_2 (kg/cm^2)	3.85 ± 0.24	3.91 ± 0.66	0.8606
Rest VCO_2 (kg/cm^2)	3.52 ± 0.70	3.33 ± 0.80	0.6947
Rest HR (bpm)	68.6 ± 4.7	68.5 ± 9.4	0.9906

Mean ± SD. P-*values* we*re for* comparing between groups *by* Student's t-test, or Fisher's exact test.

Abbreviations: BMI, body mass index; Rest HR, heart rate in a sitting position; Rest VCO2, carbon dioxide output in a sitting position; Rest VO2, oxygen uptake in a sitting position

Acknowledgments

We would like to thank Kurueme rehabilitation center for supporting the expired gas measurements.

Supporting information S1 Fig1Flow Diagram

Subjects were randomly allocated to group A (the knee bending exercise test with Hybrid training system was performed firstly) or group B (the knee bending exercise test without Hybrid training system was performed firstly) and then transitioned to the alternative exercise test on different days separated by an interval of one week as a wash out period.

S2 Fig 2. Exercise scene.

Subjects lay in a supine position on a flat surface with their quadriceps electrically stimulated as they attempted to bend their knee and their

hamstrings electrically stimulated as they attempted to extend their knee. The timing of the electrical stimulation was controlled by a joint motion sensor attached to the knee. They bent and stretched their left and right lower limbs alternately.

S3 Fig 3. Comparing of changes of oxygen uptake.

HTS, hybrid training system; #, $P < 0.05$; ##, $P < 0.01$.

S4 Fig4. Comparing of changes of carbon dioxide production.

HTS, hybrid training system; #, $P < 0.05$.

S5 Fig5. Comparing of changes of heart rate.

HTS, hybrid training system.

References

1. Nagaraja MP, Jo H. The Role of Mechanical Stimulation in Recovery of Bone Loss-High versus Low Magnitude and Frequency of Force. Life (Basel). 2014;4(2):117-30. Epub 2014/11/06. doi: 10.3390/life4020117. PubMed PMID: 25370188; PubMed Central PMCID: PMCPMC4187165.
2. Valenzuela PL, Morales JS, Pareja-Galeano H, Izquierdo M, Emanuele E, de la Villa P, et al. Physical strategies to prevent disuse-induced functional decline in the elderly. Ageing Res Rev. 2018;47:80-8. Epub 2018/07/22. doi: 10.1016/j.arr.2018.07.003. PubMed PMID: 30031068.
3. Bloomfield SA. Changes in musculoskeletal structure and function with prolonged bed rest. Med Sci Sports Exerc. 1997;29(2):197-206. Epub 1997/02/01. doi: 10.1097/00005768-199702000-00006. PubMed PMID: 9044223.
4. Voet NB, van der Kooi EL, van Engelen BG, Geurts AC. Strength training and aerobic exercise training for muscle disease. Cochrane Database Syst Rev. 2019;12:CD003907. Epub 2019/12/07. doi: 10.1002/14651858.CD003907.pub5. PubMed PMID: 31808555; PubMed Central PMCID: PMCPMC6953420.
5. Maffiuletti NA, Green DA, Vaz MA, Dirks ML. Neuromuscular Electrical Stimulation as a Potential Countermeasure for Skeletal Muscle Atrophy and Weakness During Human Spaceflight. Front Physiol. 2019;10:1031. Epub 2019/08/29. doi: 10.3389/fphys.2019.01031. PubMed PMID: 31456697; PubMed Central PMCID: PMCPMC6700209.
6. Brower RG. Consequences of bed rest. Crit Care Med. 2009;37(10 Suppl):S422-8. Epub 2010/02/06. doi: 10.1097/CCM.0b013e3181b6e30a. PubMed PMID: 20046130.
7. Dirks ML, Wall BT, van Loon LJC. Interventional strategies to combat muscle disuse atrophy in humans: focus on neuromuscular electrical stimulation and dietary protein. J Appl Physiol (1985). 2018;125(3):850-61. Epub 2017/10/04. doi: 10.1152/japplphysiol.00985.2016. PubMed PMID: 28970205.
8. Gomes Neto M, Oliveira FA, Reis HF, de Sousa Rodrigues E, Jr., Bittencourt HS, Oliveira Carvalho V. Effects of Neuromuscular Electrical Stimulation on Physiologic and Functional Measurements in Patients With Heart Failure: A SYSTEMATIC REVIEW WITH META-ANALYSIS. J Cardiopulm Rehabil Prev. 2016;36(3):157-66. Epub 2016/01/20. doi: 10.1097/HCR.0000000000000151. PubMed PMID: 26784735.
9. Hamada T, Hayashi T, Kimura T, Nakao K, Moritani T. Electrical stimulation of

human lower extremities enhances energy consumption, carbohydrate oxidation, and whole body glucose uptake. J Appl Physiol (1985). 2004;96(3):911-6. Epub 2003/11/05. doi: 10.1152/japplphysiol.00664.2003. PubMed PMID: 14594864.

10. Paillard T. Combined application of neuromuscular electrical stimulation and voluntary muscular contractions. Sports Med. 2008;38(2):161-77. PubMed PMID: 18201117.

11. Dudley GA, Tesch PA, Miller BJ, Buchanan P. Importance of eccentric actions in performance adaptations to resistance training. Aviat Space Environ Med. 1991;62(6):543-50. PubMed PMID: 1859341.

12. Seger JY, Thorstensson A. Electrically evoked eccentric and concentric torque-velocity relationships in human knee extensor muscles. Acta Physiol Scand. 2000;169(1):63-9. Epub 2000/04/12. doi: 10.1046/j.1365-201x.2000.00694.x. PubMed PMID: 10759612.

13. Pain MT, Young F, Kim J, Forrester SE. The torque-velocity relationship in large human muscles: maximum voluntary versus electrically stimulated behaviour. J Biomech. 2013;46(4):645-50. Epub 2013/01/15. doi: 10.1016/j.jbiomech.2012.11.052. PubMed PMID: 23313275.

14. Matsuse H, Shiba N, Umezu Y, Nago T, Tagawa Y, Kakuma T, et al. Muscle training by means of combined electrical stimulation and volitional contraction. Aviat Space Environ Med. 2006;77(6):581-5. PubMed PMID: 16780234.

15. Iwasaki T, Shiba N, Matsuse H, Nago T, Umezu Y, Tagawa Y, et al. Improvement in knee extension strength through training by means of combined electrical stimulation and voluntary muscle contraction. Tohoku J Exp Med. 2006;209(1):33-40. PubMed PMID: 16636520.

16. Takano Y, Haneda Y, Maeda T, Sakai Y, Matsuse H, Kawaguchi T, et al. Increasing muscle strength and mass of thigh in elderly people with the hybrid-training method of electrical stimulation and volitional contraction. Tohoku J Exp Med. 2010;221(1):77-85. PubMed PMID: 20453461.

17. Rabe KG, Matsuse H, Jackson A, Segal NA. Evaluation of the Combined Application of Neuromuscular Electrical Stimulation and Volitional Contractions on Thigh Muscle Strength, Knee Pain, and Physical Performance in Women at Risk for Knee Osteoarthritis: A Randomized Controlled Trial. PM R. 2018;10(12):1301-10. Epub 2018/06/01. doi: 10.1016/j.pmrj.2018.05.014. PubMed PMID: 29852286; PubMed Central PMCID: PMCPMC6719317.

18. Matsuse H, Shiba N, Takano Y, Yamada S, Ohshima H, Tagawa Y. Cycling exercise to resist electrically stimulated antagonist increases oxygen uptake in males: pilot study. J Rehabil Res Dev. 2013;50(4):545-54. PubMed PMID: 23934874.

19. Ohmoto M MH, Takano Y, Yamada S, Ohshima H, Tagawa Y, Shiba N. Oxygen

Uptake during Aerobic Cycling Exercise Simultaneously Combined with Neuromuscular Electrical Stimulation of Antagonists. J Nov Physiother. 2013:3-6.

20. Gaesser GA, Poole DC. The slow component of oxygen uptake kinetics in humans. Exerc Sport Sci Rev. 1996;24:35-71. Epub 1996/01/01. PubMed PMID: 8744246.

21. Matsuse H, Segal NA, Rabe KG, Shiba N. The Effect of Neuromuscular Electrical Stimulation during Walking on Muscle Strength and Knee Pain in Obese Women with Knee Pain: A Randomized Controlled Trial. Am J Phys Med Rehabil. 2019. Epub 2019/10/09. doi: 10.1097/PHM.0000000000001319. PubMed PMID: 31592880.

22. Oates BR, Glover EI, West DW, Fry JL, Tarnopolsky MA, Phillips SM. Low-volume resistance exercise attenuates the decline in strength and muscle mass associated with immobilization. Muscle Nerve. 2010;42(4):539-46. Epub 2010/07/27. doi: 10.1002/mus.21721. PubMed PMID: 20658567.

23. Alkner BA, Tesch PA. Efficacy of a gravity-independent resistance exercise device as a countermeasure to muscle atrophy during 29-day bed rest. Acta Physiol Scand. 2004;181(3):345-57. Epub 2004/06/16. doi: 10.1111/j.1365-201X.2004.01293.x. PubMed PMID: 15196095.

24. Berg HE, Tesch A. A gravity-independent ergometer to be used for resistance training in space. Aviat Space Environ Med. 1994;65(8):752-6. Epub 1994/08/01. PubMed PMID: 7980338.

25. Burke D, Gorman E, Stokes D, Lennon O. An evaluation of neuromuscular electrical stimulation in critical care using the ICF framework: a systematic review and meta-analysis. Clin Respir J. 2016;10(4):407-20. Epub 2014/10/30. doi: 10.1111/crj.12234. PubMed PMID: 25353646.

26. Iwanaga S, Hashida R, Takano Y, Bekki M, Nakano D, Omoto M, et al. Hybrid Training System Improves Insulin Resistance in Patients with Nonalcoholic Fatty Liver Disease: A Randomized Controlled Pilot Study. Tohoku J Exp Med. 2020;252(1):23-32. Epub 2020/08/31. doi: 10.1620/tjem.252.23. PubMed PMID: 32863329.

27. Tsukada Y, Matsuse H, Shinozaki N, Takano Y, Nago T, Shiba N. Combined Application of Electrically Stimulated Antagonist Muscle Contraction and Volitional Muscle Contraction Prevents Muscle Strength Weakness and Promotes Physical Function Recovery After Total Knee Arthroplasty: A Randomized Controlled Trial. Kurume Med J. 2020;65(4):145-54. Epub 2019/11/15. doi: 10.2739/kurumemedj.MS654007. PubMed PMID: 31723080.

28. Numata H, Nakase J, Inaki A, Mochizuki T, Oshima T, Takata Y, et al. Effects of the belt electrode skeletal muscle electrical stimulation system on lower extremity skeletal muscle activity: Evaluation using positron emission tomography. J Orthop Sci.

2016;21(1):53-6. Epub 2016/01/13. doi: 10.1016/j.jos.2015.09.003. PubMed PMID: 26755387.

29. Forestieri P, Bolzan DW, Santos VB, Moreira RSL, de Almeida DR, Trimer R, et al. Neuromuscular electrical stimulation improves exercise tolerance in patients with advanced heart failure on continuous intravenous inotropic support use-randomized controlled trial. Clin Rehabil. 2018;32(1):66-74. Epub 2017/06/22. doi: 10.1177/0269215517715762. PubMed PMID: 28633534.

30. Dalise S, Cavalli L, Ghuman H, Wahlberg B, Gerwig M, Chisari C, et al. Biological effects of dosing aerobic exercise and neuromuscular electrical stimulation in rats. Sci Rep. 2017;7(1):10830. Epub 2017/09/09. doi: 10.1038/s41598-017-11260-7. PubMed PMID: 28883534; PubMed Central PMCID: PMCPMC5589775.

31. Fontes Cerqueira TC, Cerqueira Neto ML, Cacau LAP, Oliveira GU, Silva Junior WMD, Carvalho VO, et al. Ambulation capacity and functional outcome in patients undergoing neuromuscular electrical stimulation after cardiac valve surgery: A randomised clinical trial. Medicine (Baltimore). 2018;97(46):e13012. Epub 2018/11/16. doi: 10.1097/MD.0000000000013012. PubMed PMID: 30431575; PubMed Central PMCID: PMCPMC6257613.

32. Hainaut K, Duchateau J. Neuromuscular electrical stimulation and voluntary exercise. Sports Med. 1992;14(2):100-13. Epub 1992/08/01. doi: 10.2165/00007256-199214020-00003. PubMed PMID: 1509225.

33. Theurel J, Lepers R, Pardon L, Maffiuletti NA. Differences in cardiorespiratory and neuromuscular responses between voluntary and stimulated contractions of the quadriceps femoris muscle. Respir Physiol Neurobiol. 2007;157(2-3):341-7. Epub 2007/01/11. doi: 10.1016/j.resp.2006.12.002. PubMed PMID: 17210271.

34. Liu M, Luo J, Zhou J, Zhu X. Intervention effect of neuromuscular electrical stimulation on ICU acquired weakness: A meta-analysis. Int J Nurs Sci. 2020;7(2):228-37. Epub 2020/07/21. doi: 10.1016/j.ijnss.2020.03.002. PubMed PMID: 32685621; PubMed Central PMCID: PMCPMC7355203.

35. Nakamura K, Nakano H, Naraba H, Mochizuki M, Hashimoto H. Early rehabilitation with dedicated use of belt-type electrical muscle stimulation for severe COVID-19 patients. Crit Care. 2020;24(1):342. Epub 2020/06/17. doi: 10.1186/s13054-020-03080-5. PubMed PMID: 32539827; PubMed Central PMCID: PMCPMC7294763.

36. Swain DP, Leutholtz BC. Heart rate reserve is equivalent to %VO2 reserve, not to %VO2max. Med Sci Sports Exerc. 1997;29(3):410-4. Epub 1997/03/01. doi: 10.1097/00005768-199703000-00018. PubMed PMID: 9139182.

Figure1

Click here to access/download;Figure;S1_Fig1.tif

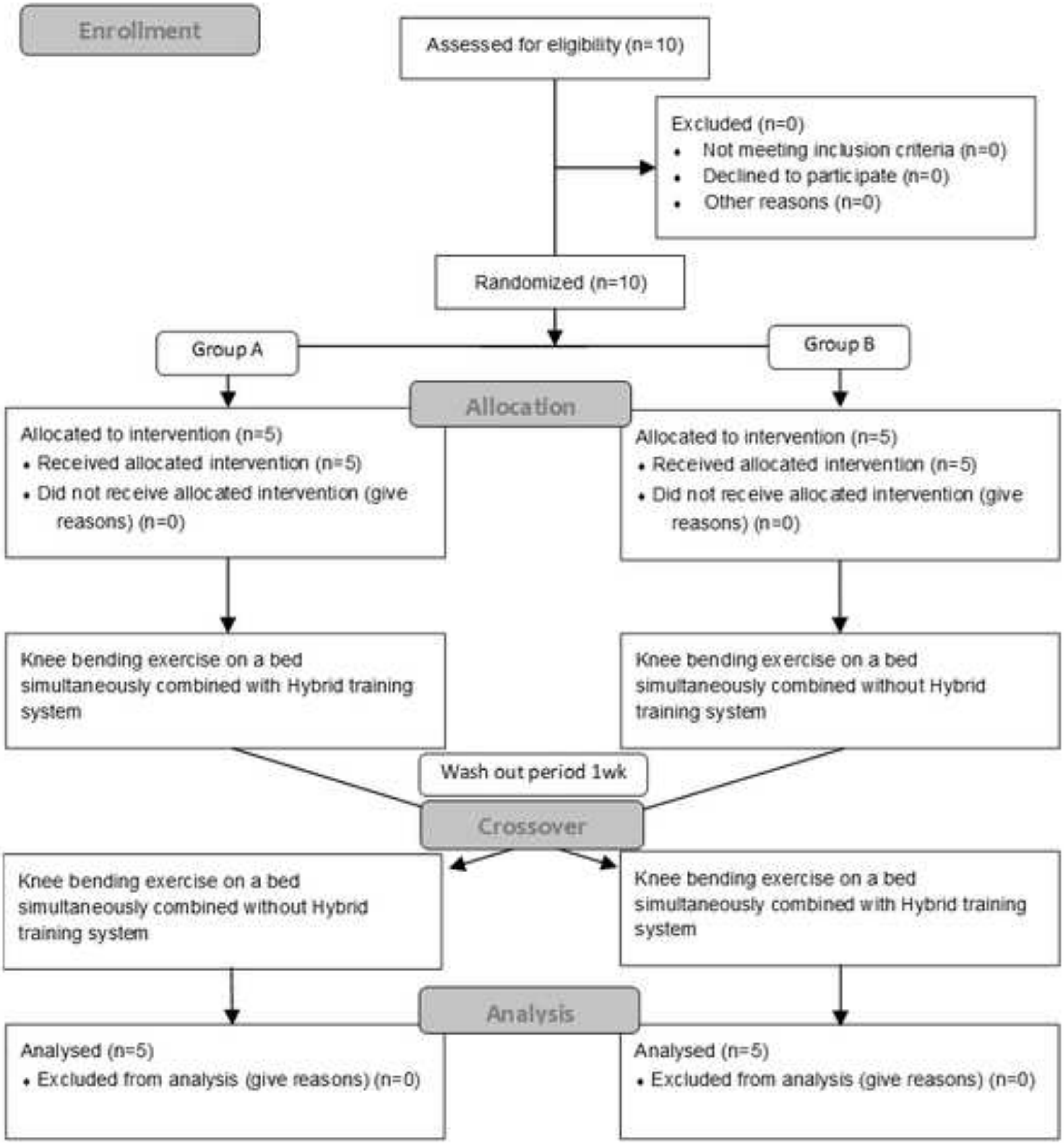

Figure2

Click here to access/download;Figure;S2_Fig2.tif

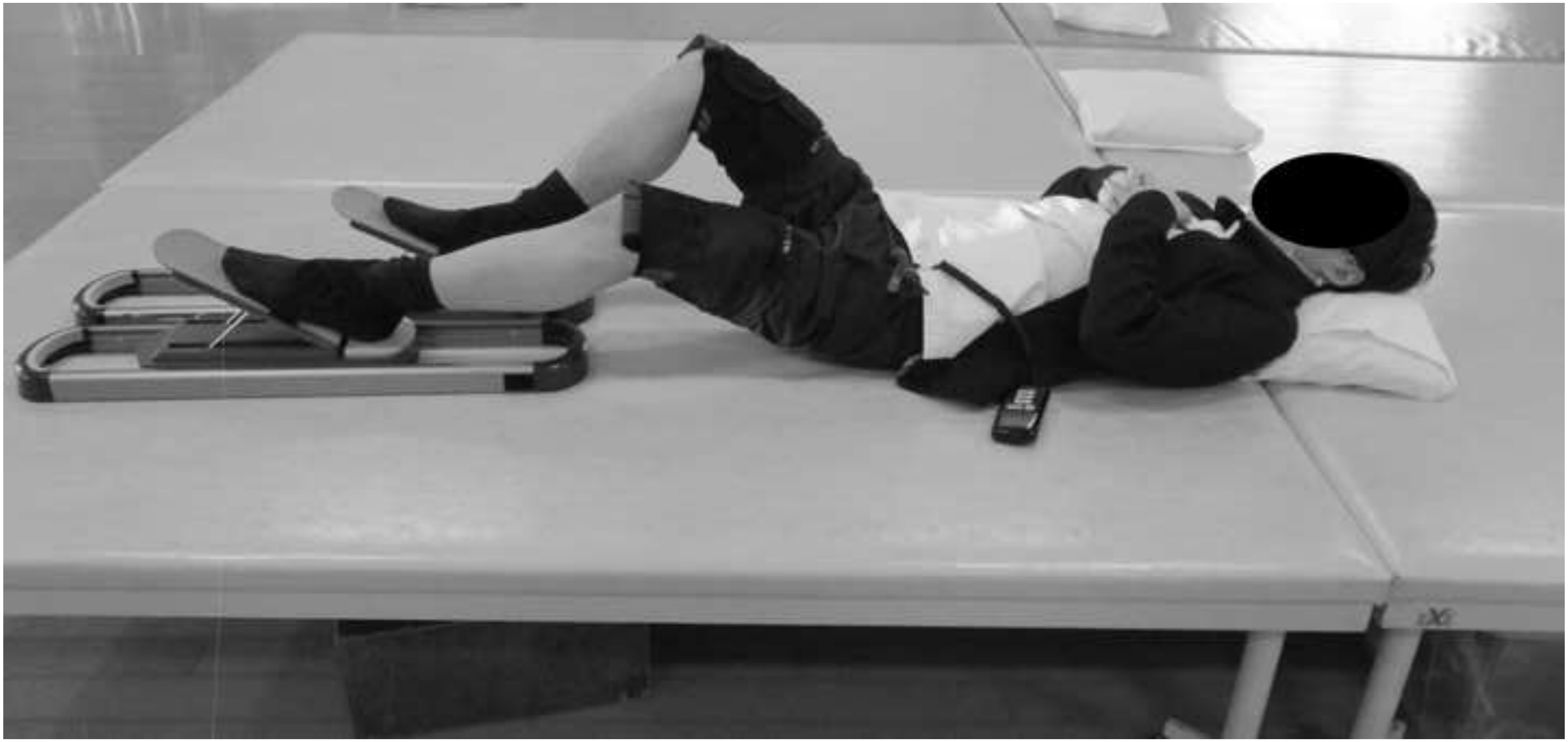

 Click here to access/download;Figure;S3_Fig3.tif

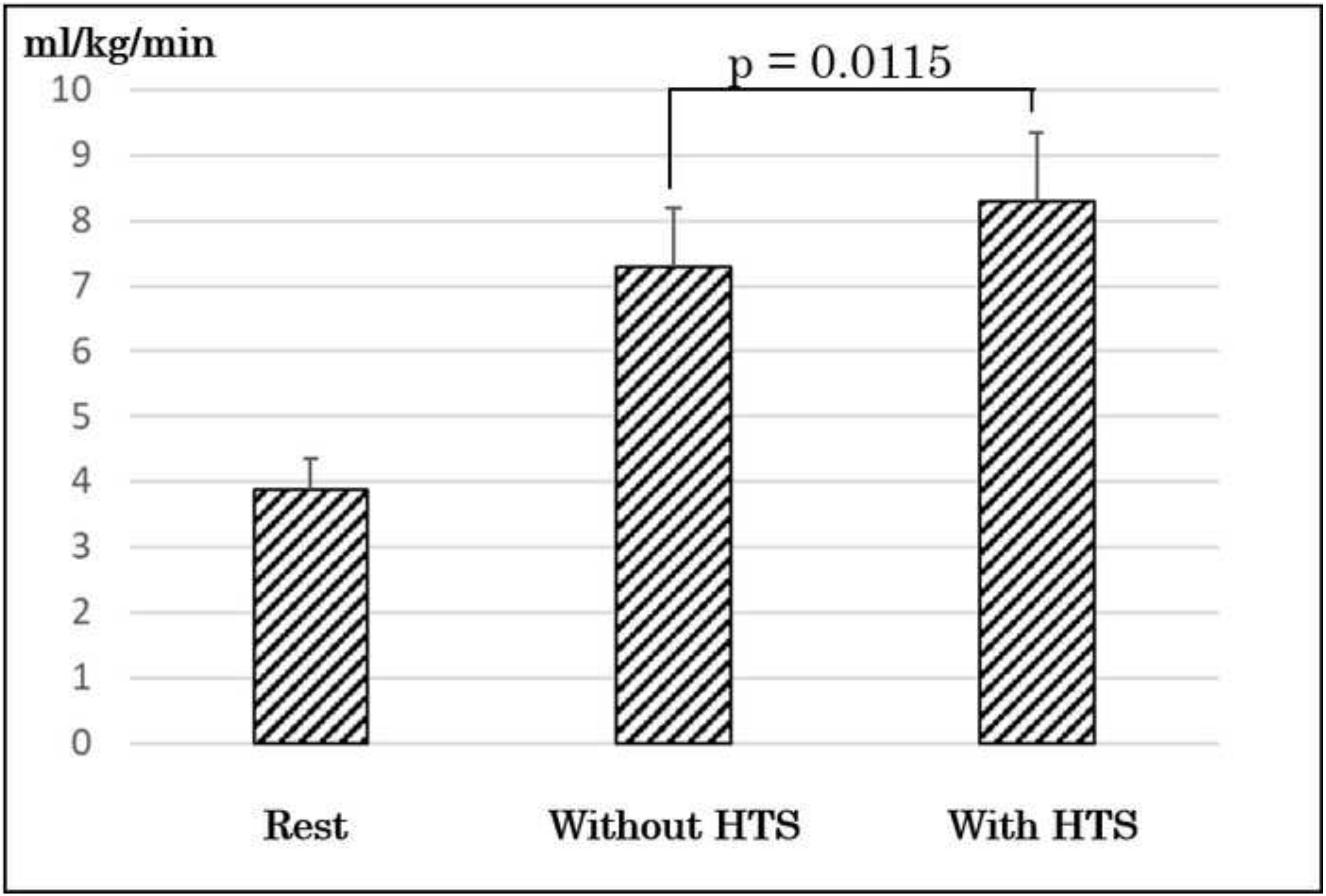

Figure4

Click here to access/download;Figure;S4_Fig4.tif

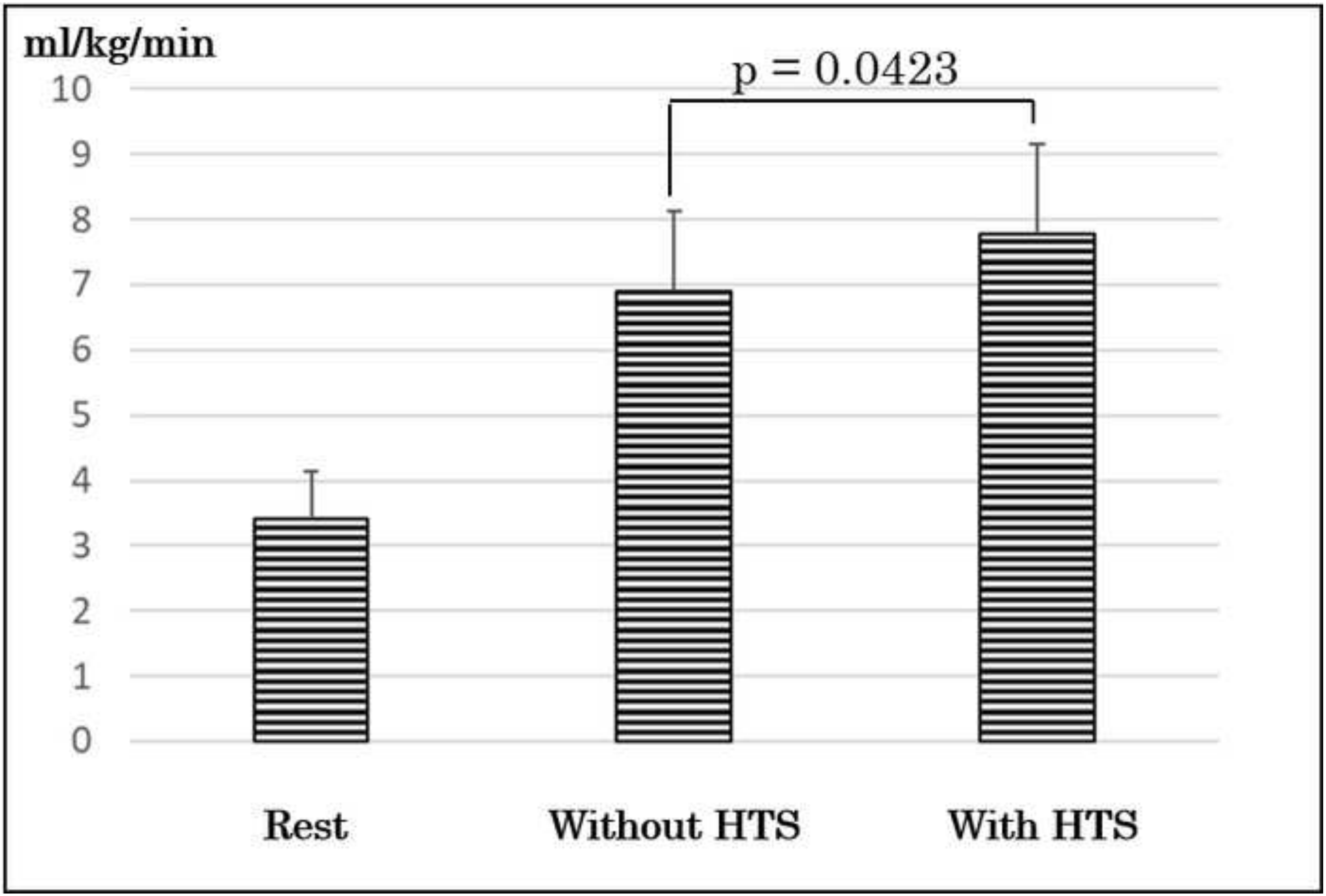

Figure5

Click here to access/download;Figure;S5_Fig5.tif

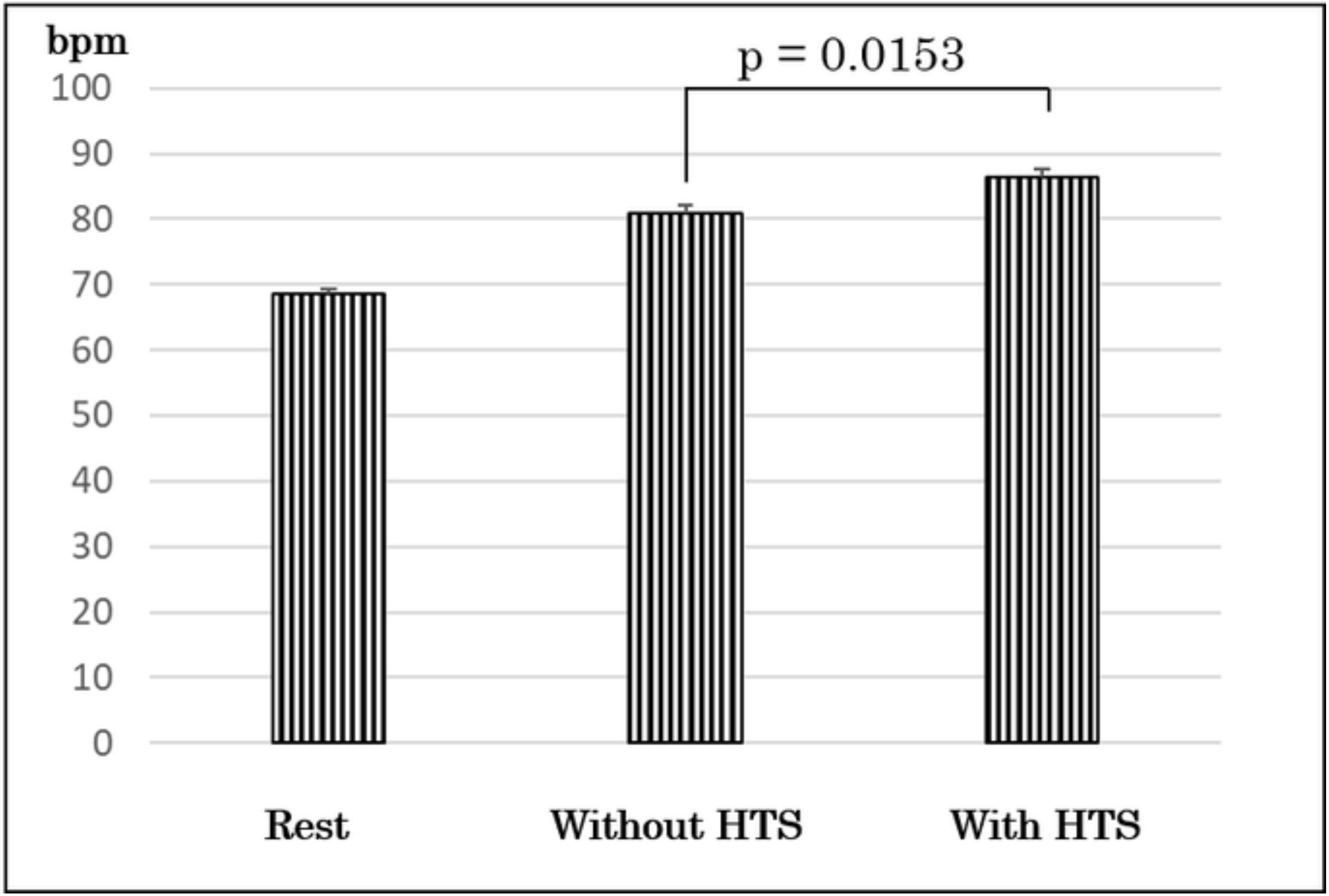

Click here to access/download
Supporting Information
CONSORT 2010 Checklist knee bending exercise.doc

Click here to access/download
Supporting Information
Knee bending exercise study protocol for IRB (Englsh).pdf

Study protocol (Original language)

Click here to access/download
Supporting Information
Knee bending exercise study protocol for IRB (
Japanese).pdf

Electrically stimulated eccentric contraction during non-weight bearing knee bending exercise in the ~~dorsal~~ supine position on a bed increases oxygen uptake: a randomized, controlled, exploratory crossover trial

[1]Hiroshi Tajima, M.D., [1]Hiroo Matsuse, M.D., Ph.D., [1]Ryuki Hashida, M.D., Ph.D., [1]Takeshi Nago, M.D., Ph.D., [1]Masafumi Bekki, M.D., Ph.D.,[1]Sohei Iwanaga, M.D., Ph.D., [1]Eriko Higashi, M.D., [1]Naoto Shiba, M.D., Ph.D.

1 Rehabilitation center, Kurume University, 67 Asahi-machi, Kurume, Fukuoka 830-0011, JAPAN

Address for corresponding with the author: Hiroo Matsuse, M.D., Ph.D. Rehabilitation center, Kurume University, 67 Asahi-machi, Kurume, Fukuoka 830-0011, Japan. Tel: +81-(0)942-31-7568, Fax: +81-(0)942-46-6002 E-mail: matsuse_hiroh@kurume-u.ac.jp

Running title: Oxygen uptake during knee bending on a bed

Author contributions:

1. Hiroyuki Tajima wrote the manuscript and mainly carried out all the experiments.

2. Naoto Shiba and Hiroo Matsuse managed the exercise method combined with hybrid training system.

3. Masafumi Bekki, Sohei Iwanaga, and Eriko Higashi measured and calculated the data.

4. Takeshi Nago and Ryuki Hashida managed this experiment.

Disclosures:

This research did not receive any specific grant from funding agencies in the public, commercial, or not-for-profit sectors. All authors declare that they have no competing interests in this study.

Abbreviation’s list:

ES, electrical stimulation; HTS, hybrid training system; HR; heart rate; NMES, neuromuscular electrical stimulation; METs, Metabolic equivalents; RER, respiratory exchange ratio; VC, volitional contraction; VCO2, carbon

dioxide output; VO2, oxygen uptake

Abstract

It is well known that prolonged bed rest induces muscle weakness, muscle atrophy, cardiovascular deconditioning, bone loss, a loss of functional capacity, and the development of insulin resistance. Neuromuscular electrical stimulation is anticipated to be an interventional strategy for disuse due to bed rest. A hybrid training system (HTS), synchronized neuromuscular electrical stimulation for voluntary exercise using an articular motion sensor, may increase the exercise load though bed rest. We assessed oxygen uptake or heart rate during knee bending exercise in the supine position on a bed both simultaneously combined with HTS and without HTS to evaluate exercise intensity on different days in ten healthy subjects (8 men and 2 women) by a randomized controlled crossover trial. The values of relative oxygen uptake during knee bending exercise with HTS were significantly greater than those during knee bending exercise without HTS (7.29 ± 0.91 ml/kg/min vs. 8.29 ± 1.06 ml/kg/min; p = 0.0115). That increment with HTS was a mean ~~an average~~ of 14.42 ± 13.99%. Metabolic equivalents during knee bending exercise with HTS and without HTS were 2.08 ± 0.26 and 2.39 ± 0.30, respectively. The values of heart rate

Formatted: Font color: Red

during knee bending exercise with HTS were significantly greater than those during knee bending exercise without HTS (80.82 ± 9.19 bpm vs. 86.36 ± 5.50 bpm; $p = 0.0153$). HTS could increase exercise load during knee bending exercise which is easy to implement on a bed. HTS might be a useful technique as a countermeasure against the disuse due to bed rest, for example during acute care or the quarantine for infection prophylaxis.

Key words: Analysis of expired gas, Bed rest, Disuse, Metabolic equivalents, Neuromuscular electrical stimulation

Introduction

It is well known that muscle atrophy, cardiovascular deconditioning, bone loss, a loss of functional capacity, and the development of insulin resistance due to disuse occurs in astronauts [1, 2]. Bed rest causes similar disuse[3] although bed rest has been prescribed in the past for several other clinical conditions such as the rest for the intensive care or the quarantine for prevention of infection[3]. Strength training or aerobic exercise programs are expected to prevent disuse atrophy and deconditioning [4]. However, the evidence regarding interventions involving either strength training alone or aerobic exercise alone remains uncertain [4]. Moreover, some patients in acute care hospitals have difficulty even standing. Some patients aren't able to do exercise at intensity 3 Metabolic equivalents (METs) –a such as walking. We need relatively efficient and simple exercise that can be performed on a bed to prevent disuse during bed rest.

Neuromuscular electrical stimulation (NMES) is widely used to lessen the complications of disuse during spaceflight or bed rest by increasing muscle strength and mass [5]-[6]-[7]. Moreover, it is well known

Field Code Changed

Field Code Changed

Field Code Changed

Field Code Changed

that NMES upregulates glucose metabolism and peak oxygen uptake [8]-[9]. In addition, the combined application of electrical stimulation (ES) and volitional contractions (VC) (performing ES during VC either non-simultaneously or simultaneously) is said to be more effective than ES or VC alone [10]. The hybrid training system (HTS) is one of the techniques that simultaneously ~~combin~~combines~~ing~~ VC and ES, and is able to be used during exercises such as ergometer walking. HTS utilizes electrically stimulated eccentric contractions. Eccentric contractions are important for muscle strengthening[11]. Furthermore, electrically stimulated eccentric contractions are recognized as a muscle strengthening technique, because we can obtain bigger muscle torque (exercise load) than with concentric contractions alone[12, 13]. It has been reported that HTS can increase both muscle strength and mass even when exercise intensity is relatively low[14-17]. Moreover, ~~W~~we have reported that ergometer exercise using HTS leads to a greater increase in metabolic cost [18, 19]. Knee bending exercise, one of the common exercises on a bed, is recommended for patients with disuse syndrome ~~who are not able to walk~~ in a clinical situation such as the case that cannot walk for the reason of the motor disfunction or the case to be

Formatted: Font color: Red

Field Code Changed

Field Code Changed

Field Code Changed

Field Code Changed

Field Code Changed

forced to lay for a remedial reason. Therefore, we hypothesize that by combining HTS with knee bending exercise may provide exercise intensity that can easily be performed on a bed. It may be a useful exercise technique for the prevention of disuse due to bed rest.

The purpose of the present study is to compare the metabolic cost between knee bending exercise on a bed with and without HTS by analyzing expired gas.

Methods

Subjects

This study protocol was approved by the Ethics Committee of Kurume University (No.19209) and registered at UMIN Clinical Trials Registry (UMIN000039336). Following approval, written informed consent was obtained from 10 healthy (8 men and 2 women, age 28.9 years (SD = 8.5~~)~~)) who had reviewed the goals of the study and agreed to participate. The subjects of this study were healthy participants as preliminary research before intending for the clinical patients. When participants were enrolled,

the staff who was not involved in intervention and evaluation randomized the intervention sequence using a random number generator and sealed opaque envelopes with a block size of 5 to allocate to group A (the knee bending exercise test with HTS was performed firstly) or group B (the knee bending exercise test without HTS was performed firstly) (Fig 1). ~~Inclusion criteria required that the subjects undergo an examination for normal physical fitness, strength, sensation, and range of motion according to the criteria of the Japanese Orthopedic Association.~~ Inclusion criteria were applied: age between 20-50 years, nonsmokers, body mass index less than 30, doing normal physical activity, and normal physical function. A specialist in expert orthopedics and rehabilitation judged the normal physical function from the physical findings such as muscle strength, sensation, and range of motion according to the criteria of the Japanese Orthopedic Association. Exclusion criteria were that the subjects had: a musculoskeletal problem, adverse medical history affecting cardiorespiratory function, or some kind of medical treatment. We set these criteria in order to minimize possible confounding effects of subject characteristics over the exercise tests in this study. They were also not to participate in any regular sports activities

Formatted: Font color: Red, Strikethrough

during the study period.

Design

We performed two cardiopulmonary exercise tests in the clinical laboratory of the rehabilitation center by a randomized, controlled, crossover trial (Fig. 1). It is very difficult for both subject and researcher to completely blind presence or absence of electrical stimulation. Moreover, we need to minimize the influence of the individual difference. Therefore, we chose a crossover trial in this study. Environmental conditions were similar for all exercise tests (21 to 24 degrees centigrade, 45 to 55 % relative humidity). We measured gas exchange while subjects did knee bending exercises with a RISTA board (NIPPON SIGMAX Co, Tokyo, Japan) atwith the lowest exercise resistance load out of three phases using the following protocol after a rest for 30 minutes. The RISTA board is a Japanese medical device to facilitate knee bending exercises in the supine position on a bed. We used the device to achieve a stable knee bending exercise on a flat surface. During the tests, gas exchange data was collected continuously with an automated breath by breath system (AE-100i, Minato Medical Science Co.

Ltd., Osaka, Japan) using the standard technique. The AE-100i consists of a microcomputer, a hot wire flowmeter, and a gas analyzer, which contains a sampling tube, filter, suction pump, oxygen (O_2) analyzer made by a paramagnetic O_2 transducer, and an infrared carbon dioxide (CO_2) analyzer. Ventilatory parameters were measured using a hot-wire flow meter, and the flow meter was calibrated with a syringe of known volume (2.0l). A zirconium sensor and an infrared absorption analyzer, respectively, measured O_2 and CO_2 concentrations. The gas analyzer was calibrated to known standard gas levels (O_2 15.16%, CO_2 5.023%) before each test. The intraclass correlation coefficients using Case 2 (2,1) for VO_2 of the primary outcome was 0.876 in our clinical laboratory. Also, heart rates (HR, beats/min) were continuously monitored by electrocardiogram during the tests. O_2 and ~~carbon dioxide output (~~CO_2~~)~~ were calculated and recorded during the following exercise tests.

Intervention

The knee bending exercise test was performed as two tests (with HTS or without HTS) for analysis of expired gas. Participants rested for 30 min in

the supine position to minimize the influence of physical activity before the exercise test. At first subjects rested for five minutes in a sitting position to evaluate of the basic inactivity quiet metabolic cost while sitting quietly. Then, participants performed the knee bending exercise tests with or without HTS according to a sequence assigned before the exercise test. The sequence of the knee bending exercise tests with or without HTS was performed at random, and Next, participants transitioned to the alternative exercise test on different days separated by an interval of one week as a wash out period. Therefore, the participant who first performed the exercise test with HTS performed the exercise test without HTS next. When participants were enrolled, the staff who was not involved in intervention and evaluation randomized the intervention sequence using a random number generator and sealed opaque envelopes with a block size of 5. At first subjects rested for five minutes in a sitting position for rest. NextIn the interventional exercise, subjects performed the knee bending exercise with reciprocal 2-second (45degree/sec) knee flexion and extension contractions at the speed of 1Hz using a metronome for 5 minutes with HTS or without HTS in the supine position on a flat surface after warming up at a slow

Formatted: Font color: Red

Formatted: Font color: Red

Formatted: Font color: Red

Formatted: Font: +Body (Century), Font color: Red

Formatted: Font color: Red

speed for 3 minutes (Fig 2). The sequence of the knee bending exercise tests with or without HTS was performed at random, and on different days separated by an interval of one week as a wash out period. When participants were enrolled, the staff who was not involved in intervention and evaluation randomized the intervention sequence using a random number generator and sealed opaque envelopes with a block size of 5. Successively, subjects performed 2 minutes cool down. Therefore, We setthey had the total exercise time offor 10 minutes including 3 minutes warm up and 2 minutes cool downin each of two tests in consideration of muscle fatigue from ES. The knee joint range of motion was set at a nearly 90° arc that extended from 20° to 110° (0° indicating full knee extension). At the onset of constant-load exercise, O2 increases in healthy individual's mono-exponentially with a time constant to achieve a steady state below the lactate threshold within about 3 minutes [20]. Therefore, we averaged the data of HR and expired gas during the last 2 minutes of the interventional exercise time of 5 minutes of each test and used them for data analysis.

HTS protocol

Field Code Changed

HTS was combined with knee bending exercise in the supine position on a flat surface with an electrical stimulator (HIZA TRAINER, EU-JLM50S, Panasonic Corporation, 1006 Ohaza-Kadoma, Kadoma City, Osaka, Japan). This provided a constant voltage stimulus to the skin electrodes (regulated voltage)". This stimulator is household medical equipment including a wrapping band to fix the device and acceleration sensors or electrodes during exercise. The acceleration sensors act as joint motion sensors (EWTS9PD, Home Appliances Development Center Corporate Engineering Division, Appliances Company Panasonic Corporation 2-3-1-2 Noji-higashi, Kusatsu City, Shiga, Japan) and are placed on the front of each leg 88 mm above the patellar edge. The sensors analyze the algorithm of each gait pattern, and stimulate the flexor or extensor in accordance with the motion of each bilateral knee joint during knee bending exercise (Figs 2 and 3). Electrodes (Sekisui Plastics Co., Tokyo, Japan) coated with an oxidation-resistant silver-carbon compound and were placed over the quadriceps on the anterior thigh, and over the hamstrings on the posterior thigh with low impedance gel. The size of the electrodes was 15cm × 5cm for the quadriceps and 10cm × 6cm for the hamstrings. ES stimulated the quadriceps during

knee flexion. Conversely, ES stimulated the hamstrings during knee extension. Therefore, the electrically stimulated muscles were eccentric contractions. ES parameters were based on a standard Russian waveform in which a 5,000 Hz carrier frequency is modulated at 40 Hz (2.4 ms on, 22.6 ms off) to deliver a rectangular voltage biphasic pulse. ES intensity was set to approximately 80% of the subject's maximum tolerance. This intensity has been reported to successfully improve muscle strength and mass without causing pain or numbness [16, 17, 21].

Fig 2. Exercise scene.

Fig 2. Schematic model of hybrid training system.

Subjects lay in a supine position on a flat surface .They bent and stretched their left and right lower limbs alternately.with their quadriceps electrically stimulated as they attempted to bend their knee and their hamstrings electrically stimulated as they attempted to extend their knee. The timing of the electrical stimulation was controlled by a joint motion sensor attached to the knee.

Field Code Changed

Formatted: Font color: Red

~~Fig 3. Exercise scene.~~

~~Subjects lay on their back on a bed.~~ ~~They bent and stretched their left and right lower limbs alternately.~~

Statistical Analysis

All variables are presented as means ± SD. This study was not set to the sample size for an exploratory study. The original study plan recruited 20 participants, but we analyzed this study because we were able to achieve this study purpose from ten participants. We compared the participants' characteristics and rest values (relative VO2, VCO2, and HR) between groups (two sequences) by Student's t-test, or Fisher's exact test. Values for relative VO2, VCO2, HR, and respiratory exchange ratio (RER) during exercise test were analyzed with linear mixed model that included fixed effects of treatment (with HTS or without HTS), period, and sequence. ~~and~~ Then, we set subjects(sequence) as the covariance parameter for the random effect~~random effect of subjects~~. Next, values for relative VO2, VCO2, and HR were assessed using a ~~student~~ paired Student's t-test as a post hoc test in order to compare the differences between the knee bending exercise tests

with and without HTS for the main purpose of this study. Moreover, we calculated the effect size (r) to know the strength of association between intervention and change on VO2 of the primary outcome. All the statistical analyses were performed using JMP Version 14.0 statistical software (SAS Institute Inc., Cary, NC, USA) and p values < 0.05 were considered to be statistically significant.

Results

There were not any problems and adverse events in the exercise tests for subjects such as not being able to continue from pain or fatigue. Each five subjects participated in two sequences to be shown in the flow diagram (Fig. 1), and all ten subjects finished all tests and were analyzed.

There were no significant differences at participants' characteristics and rest values (relative VO2, VCO2, and HR) between groups (Table1). There were no significant differences at period and sequence in values for relative VO2, VCO2, and HR during exercise test though there was

significant difference at treatment (with HTS or without HTS). As a primary outcome, the values of relative VO2 during knee bending exercise with HTS were significantly greater than those during knee bending exercise without HTS (7.29 ± 0.91 ml/kg/min vs 8.29 ± 1.06 ml/kg/min; $p = 0.0115$) (Fig 3~~4~~). Mean within subject difference (95% confidence interval) in the values of relative VO2 between treatments (with HTS and without HTS) was 0.998 (0.3170-1.6785). The ~~estimated~~ effect size~~ (95% confidence interval)~~ was 0.76~~0.4219 (0.0520-0.9839)~~. The increment using HTS was ~~an average~~ mean of 14.42 ± 13.99%. Metabolic equivalents ~~(METs)~~ during knee bending exercise with HTS and without HTS were 2.08 ± 0.26 and 2.39 ± 0.30, respectively. The values of relative VCO2 during knee bending exercise with HTS were significantly greater than those during knee bending exercise without HTS (7.29 ± 0.91 ml/kg/min vs 8.29 ± 1.06 ml/kg/min; $p = 0.0423$) (Fig ~~5~~4). There was not a significant difference between the values of RER during knee bending exercise with HTS and without HTS (0.94 ± 0.08 vs 0.94 ± 0.10; $p = 0.86$). The values of HR during knee bending exercise with HTS were significantly greater than without HTS (80.82 ± 9.19 bpm vs 86.36 ± 5.50 bpm; $p = 0.0153$) (Fig ~~6~~5).

Fig. 34. Comparing of changes of oxygen uptake.**Changes of oxygen uptake (VO2).**

Formatted: Font color: Red

The values of relative VO2 during knee bending exercise with HTS were significantly greater than those during knee bending exercise without HTS (7.29 ± 0.91 ml/kg/min vs 8.29 ± 1.06 ml/kg/min; p = 0.0115). The increment by HTS was an average of 14.42 ± 13.99%. O2, oxygen consumption; HTS, hybrid training system.

Fig.45. Comparing of changes of carbon dioxide production.**Changes of carbon dioxide production (VCO2).**

Formatted: Font color: Red

The values of relative VCO2 during knee bending exercise with HTS were significantly greater than those during knee bending exercise without HTS (7.29 ± 0.91 ml/kg/min vs 8.29 ± 1.06 ml/kg/min; p = 0.0423). CO2, carbon dioxide production; HTS, hybrid training system.

Fig. 56. Comparing of changes of heart rate.**Changes of heart rate (HR).**

The values of HR during knee bending exercise with HTS were significantly

~~greater than those during knee bending exercise without HTS (80.82 ± 9.19 bpm vs 86.36 ± 5.50 bpm; p = 0.0153). HR, heart rate; HTS, hybrid training system.~~

Discussion

This is the first study of the metabolic cost of knee bending exercise combined with NMES that is easily done in the~~a~~ ~~reclining~~ supine position. This study showed that HTS, utilizing electrically stimulated eccentric muscle contractions as a resistance to voluntary exercise, increased exercise intensity during knee bending exercise on a bed. HTS may be a useful exercise technique to prevent disuse during bed rest by providing augmentation of local mechanical stress and systemic metabolic stress.

The decrement in mechanical stress caused by bed rest or spaceflight causes muscle weakness, muscular atrophy, bone atrophy, and the loss of cardiovascular capacity. Resistance exercise is an effective countermeasure for the prevention of muscle atrophy [2] even if it is relatively low volume of resistance exercise [22]–[23]. However, Bryan. R.

Field Code Changed

Field Code Changed

Field Code Changed

Oates et al. [22] used an exercise intensity of 80% of maximal with resistance exercise. It is difficult for clinical patients to achieve adequate exercise intensity on a bed. The flywheel ergometer has been used for resistance training in space flight. The resistance was provided independent of gravity by using the inertial focus of a flywheel [24]. B. A. Alkner and P. A. Tesch [23] reported the utility of the flywheel ergometer on the bed. However, it is difficult to use a flywheel ergometer for clinical applications because it is a special device. NMES is widely used in clinic settings and can easily generate sufficient muscle contractions without relying on gravity. Therefore, NMES could be useful as a countermeasure against disuse due to bed rest. It could be especially useful when effective voluntary exercise is not possible due to disturbance of consciousness or strict medical management [25]. However, VC is undoubtedly the most basic effective countermeasure for disuse. In this study, the knee bending exercise which was easily performed in ~~a~~ the supine position on a flat surface was approximately 2 METs in exercise intensity. Furthermore, we can make up for the detriments and use them to advantage by combining VC with ES [18] [19, 26, 27]. Moreover, ES should ideally be applied synchronously with

Field Code Changed

Field Code Changed

Field Code Changed

Field Code Changed

voluntary exercise to obtain synergistic benefits [2]. In this study, we could increase exercise intensity by approximately 14.4% of the by combining ES with the knee bending exercise in the supine position on a flat surface. Therefore, we could strengthen the exercise intensity even though in ~~a~~ the supine position on a flat surface by using HTS more effectively.

In addition, aerobic exercise is an effective countermeasure against the loss of cardiovascular capacity associated with disuse [2]. Generally, the metabolic rate during complete bed rest is less than 1 MET. Physical activity is undoubtedly the most effective countermeasure against disuse. The knee bending exercise in this study resulted in approximately 2 METs of metabolic rate. NMES stimulates metabolic response [28] and energy consumption, carbohydrate oxidation, and whole-body glucose uptake [9]. Therefore, we may use NMES as aerobic exercise to improve physical capacity [29]. However, NMES did not change maximum capacity although aerobic exercise did in rats [30]. Additionally, NMES did not improve functional capacity in cardiac valve surgery patients in the immediate postoperative period [31]. Therefore, the aerobic exercise effect of NMES as a countermeasure against disuse due to bed rest may be limited. ES

Field Code Changed

Field Code Changed

Field Code Changed

Field Code Changed

Field Code Changed

Field Code Changed

Field Code Changed

activation is considered nonselective with regard to the type of motor unit and synchrony, and it preferentially activates Type II fibers as compared with VC [32]. Jean Theurel et al. showed that the O2 and RER were significantly higher during ES compared with VC to compare respiratory gas exchange variables between equal-intensity (i.e., same force output) ES and VC of the quadriceps [33]. They considered that these findings probably reflect differences in the patterns of motor unit recruitment between ES and VC. On the other hand, in this study, there was not a significant difference between the values of RER between HTS and VC although O2 during HTS was significantly greater than for VC (without HTS). Combining VC with ES simultaneously seems to have been responsible for those results. Similarly, in a past study, we showed that HTS causes an increase of approximately 4.4% of O2 during unloaded cycling without a significant difference in RER [18]. Moreover, HTS caused an increase of approximately 21.1% of O2 during aerobic cycle ergometer exercise at moderate-intensity [19]. In this study, HTS caused an increase of approximately 14.4% of O2 during knee bending exercise in the supine position on a flat surface. In addition, the METs for these studies were approximately 2.39, which is

equivalent to light effort such as housework or light exercise. Moreover, a utility of NMES was shown in disuse prevention or the complications prevention in the acute care [34]. Furthermore, NMES is expected as one of the effective exercise therapies during the quarantine for COVID-19 patients [35]. These findings showed the possibility that we ~~could~~ might strengthen the metabolic stress from increased physical activity by combining HTS with ~~the~~ a knee bending exercise that is easily performed by bed ridden patients (e.g., rest during the acute care or the quarantine for prevention of infection spread).

There are a few potential limitations of this study. Because this study is an exploratory study, the sample size was not set. At a significance level of 0.05, an effect size in the VO2 variable of 0.76, and a power of 0.80, to detect an inter-group difference in means of 0.998. Nm would require a minimum of 58 subjects for a two-treatment parallel-design study.~~There were few female subjects, therefore a future study including more women will be necessary.~~ The purpose of this study was to evaluate the basic gas exchange response to HTS combined with knee bending exercise as a technique to provide physical activity as a countermeasure against disuse due to bed rest. For

Field Code Changed

Field Code Changed

estimating exercise intensity, oxygen uptake reserve or heart rate reserve is preferable [36]. However, we did not evaluate peak oxygen consumption or peak heart rate in each subject. In addition, we did not evaluate lactic acid or glucose to evaluate the metabolic response after having done strict nutritional management. For the investigation of clinical response, it is necessary to include clinical patients. Furthermore, the group effect (sequence) was absent, but it is unclear whether we could wash out the influence of HTS in one week. Therefore, a study not implementing a crossover trial may be necessary. Additionally, muscle contraction quantity and time influence kinetic oxygen uptake. Therefore, various protocols of electrical stimulation (such as modification of frequency, pulse width, or duty cycle) will be necessary to evaluate the effect of HTS in a future study. Of course, we should perform a randomized, controlled trial design, long-term investigation study to investigate the efficacy of strength and cardiovascular capacity of HTS for patients with disuse.

Field Code Changed

Conclusion

These findings indicate that the combined application of VC and EC could lead to a greater increase in metabolic cost during knee bending exercise in the supine position on a flat surface. Knee bending exercise simultaneously combined with HTS might be useful as a countermeasure against disuse due to bed rest by enforcing metabolic stress as well as mechanical stress.

Table 1. Participants' characteristics.

Characteristics	Group A	Group B	p-value
**Age (years)**	27.6 ± 7.3	30.2 ± 10.3	0.6580
Sex (male/female)	4/1	4/1	1.00
Body mass (kg)	66.1 ± 8.6	59.8 ± 10.2	0.3244
Height (cm)	167.8 ± 5.9	168.4 ± 13.3	0.9335
BMI (kg/m^2)	23.4 ± 1.6	21.1 ± 3.1	0.1861
Rest VO_2 (kg/cm^2)	3.85 ± 0.24	3.91 ± 0.66	0.8606
Rest VCO_2 (kg/cm^2)	3.52 ± 0.70	3.33 ± 0.80	0.6947
Rest HR (bpm)	68.6 ± 4.7	68.5 ± 9.4	0.9906

Mean ± SD. P*-values* we*re for* comparing between groups *by* Student's t-test, or Fisher's exact test.

Abbreviations: BMI, body mass index; Rest HR, heart rate in a sitting position; Rest VCO2, carbon dioxide output in a sitting position; Rest VO2, oxygen uptake in a sitting position

Acknowledgments

We would like to thank Kurueme rehabilitation center for supporting the expired gas measurements.~~Financial Disclosures:~~

~~All authors have declared that no competing interest exist.~~

~~Funding/Support:~~

~~This research did not receive any specific grant from funding agencies in the public, commercial, or not-for-profit sectors.~~

~~Institutional Review~~

~~The study was designed in accordance with the ethical standards of the Helsinki Declaration of 1975 and received the approval of the Ethics Committee of Kurume University.~~

~~All procedures were fully explained to the participants who gave their written informed consent to participate.~~

Formatted: Font color: Red

Supporting information ~~Figure Legends~~

Formatted: Font: 12 pt, Font color: Red

S1 Fig~~.1.—~~Flow Diagram

Formatted: Font color: Red

Subjects were randomly allocated to group A (the knee bending exercise test with Hybrid training system was performed firstly) or group B (the knee bending exercise test without Hybrid training system was performed firstly) and then transitioned to the alternative exercise test on different days separated by an interval of one week as a wash out period.

~~Fig.2. Schematic model of hybrid training system.~~

~~Subjects lay in a supine position on a flat surface with their quadriceps electrically stimulated as they attempted to bend their knee and their hamstrings electrically stimulated as they attempted to extend their knee. The timing of the electrical stimulation was controlled by a joint motion sensor attached to the knee.~~

S2 Fig 2~~.3~~. Exercise scene.

Formatted: Font color: Red

Formatted: Font color: Red

Formatted: Font color: Red

Subjects lay in a supine position on a flat surface with their quadriceps electrically stimulated as they attempted to bend their knee and their

hamstrings electrically stimulated as they attempted to extend their knee. The timing of the electrical stimulation was controlled by a joint motion sensor attached to the knee.

Subjects lay on their back on a bed. They bent and stretched their left and right lower limbs alternately.

S3 Fig. 34. Comparing of changes of oxygen uptake.Changes of oxygen uptake (VO2).

The values of relative VO2 during knee bending exercise with HTS were significantly greater than those during knee bending exercise without HTS (7.29 ± 0.91 ml/kg/min vs 8.29 ± 1.06 ml/kg/min; $p = 0.0115$). The increment by HTS was an average of 14.42 ± 13.99%. O2, oxygen consumption; HTS, hybrid training system; #, $P < 0.05$; ##, $P < 0.01$.

S4 Fig.45. Comparing of changes of carbon dioxide production.Changes of carbon dioxide production (VCO2).

The values of relative VCO2 during knee bending exercise with HTS were significantly greater than those during knee bending exercise without HTS

Formatted: Font color: Red

Formatted: Font color: Red

Formatted: Font color: Red

Formatted: Font color: Red

Formatted: Font color: Red

Formatted: Font color: Red

(7.29 ± 0.91 ml/kg/min vs 8.29 ± 1.06 ml/kg/min; p = 0.0423). CO2, carbon dioxide production; HTS, hybrid training system; #, $P < 0.05$.

S5 Fig. 56. Comparing of changes of heart rate. Changes of heart rate (HR). The values of HR during knee bending exercise with HTS were significantly greater than those during knee bending exercise without HTS (80.82 ± 9.19 bpm vs 86.36 ± 5.50 bpm; p = 0.0153). HR, heart rate; HTS, hybrid training system.

Formatted: Font color: Red

Formatted: Font color: Red

Formatted: Font color: Red

References

Field Code Changed

1. Nagaraja MP, Jo H. The Role of Mechanical Stimulation in Recovery of Bone Loss-High versus Low Magnitude and Frequency of Force. Life (Basel). 2014;4(2):117-30. Epub 2014/11/06. doi: 10.3390/life4020117. PubMed PMID: 25370188; PubMed Central PMCID: PMCPMC4187165.

2. Valenzuela PL, Morales JS, Pareja-Galeano H, Izquierdo M, Emanuele E, de la Villa P, et al. Physical strategies to prevent disuse-induced functional decline in the elderly. Ageing Res Rev. 2018;47:80-8. Epub 2018/07/22. doi: 10.1016/j.arr.2018.07.003. PubMed PMID: 30031068.

3. Bloomfield SA. Changes in musculoskeletal structure and function with prolonged bed rest. Med Sci Sports Exerc. 1997;29(2):197-206. Epub 1997/02/01. doi: 10.1097/00005768-199702000-00006. PubMed PMID: 9044223.

4. Voet NB, van der Kooi EL, van Engelen BG, Geurts AC. Strength training and aerobic exercise training for muscle disease. Cochrane Database Syst Rev. 2019;12:CD003907. Epub 2019/12/07. doi: 10.1002/14651858.CD003907.pub5. PubMed PMID: 31808555; PubMed Central PMCID: PMCPMC6953420.

5. Maffiuletti NA, Green DA, Vaz MA, Dirks ML. Neuromuscular Electrical Stimulation as a Potential Countermeasure for Skeletal Muscle Atrophy and Weakness During Human Spaceflight. Front Physiol. 2019;10:1031. Epub 2019/08/29. doi: 10.3389/fphys.2019.01031. PubMed PMID: 31456697; PubMed Central PMCID: PMCPMC6700209.

6. Brower RG. Consequences of bed rest. Crit Care Med. 2009;37(10 Suppl):S422-8. Epub 2010/02/06. doi: 10.1097/CCM.0b013e3181b6e30a. PubMed PMID: 20046130.

7. Dirks ML, Wall BT, van Loon LJC. Interventional strategies to combat muscle disuse atrophy in humans: focus on neuromuscular electrical stimulation and dietary protein. J Appl Physiol (1985). 2018;125(3):850-61. Epub 2017/10/04. doi: 10.1152/japplphysiol.00985.2016. PubMed PMID: 28970205.

8. Gomes Neto M, Oliveira FA, Reis HF, de Sousa Rodrigues E, Jr., Bittencourt HS, Oliveira Carvalho V. Effects of Neuromuscular Electrical Stimulation on Physiologic and Functional Measurements in Patients With Heart Failure: A SYSTEMATIC REVIEW WITH META-ANALYSIS. J Cardiopulm Rehabil Prev. 2016;36(3):157-66. Epub 2016/01/20. doi: 10.1097/HCR.0000000000000151. PubMed PMID: 26784735.

9. Hamada T, Hayashi T, Kimura T, Nakao K, Moritani T. Electrical stimulation of

human lower extremities enhances energy consumption, carbohydrate oxidation, and whole body glucose uptake. J Appl Physiol (1985). 2004;96(3):911-6. Epub 2003/11/05. doi: 10.1152/japplphysiol.00664.2003. PubMed PMID: 14594864.

10. Paillard T. Combined application of neuromuscular electrical stimulation and voluntary muscular contractions. Sports Med. 2008;38(2):161-77. PubMed PMID: 18201117.

11. Dudley GA, Tesch PA, Miller BJ, Buchanan P. Importance of eccentric actions in performance adaptations to resistance training. Aviat Space Environ Med. 1991;62(6):543-50. PubMed PMID: 1859341.

12. Seger JY, Thorstensson A. Electrically evoked eccentric and concentric torque-velocity relationships in human knee extensor muscles. Acta Physiol Scand. 2000;169(1):63-9. Epub 2000/04/12. doi: 10.1046/j.1365-201x.2000.00694.x. PubMed PMID: 10759612.

13. Pain MT, Young F, Kim J, Forrester SE. The torque-velocity relationship in large human muscles: maximum voluntary versus electrically stimulated behaviour. J Biomech. 2013;46(4):645-50. Epub 2013/01/15. doi: 10.1016/j.jbiomech.2012.11.052. PubMed PMID: 23313275.

14. Matsuse H, Shiba N, Umezu Y, Nago T, Tagawa Y, Kakuma T, et al. Muscle training by means of combined electrical stimulation and volitional contraction. Aviat Space Environ Med. 2006;77(6):581-5. PubMed PMID: 16780234.

15. Iwasaki T, Shiba N, Matsuse H, Nago T, Umezu Y, Tagawa Y, et al. Improvement in knee extension strength through training by means of combined electrical stimulation and voluntary muscle contraction. Tohoku J Exp Med. 2006;209(1):33-40. PubMed PMID: 16636520.

16. Takano Y, Haneda Y, Maeda T, Sakai Y, Matsuse H, Kawaguchi T, et al. Increasing muscle strength and mass of thigh in elderly people with the hybrid-training method of electrical stimulation and volitional contraction. Tohoku J Exp Med. 2010;221(1):77-85. PubMed PMID: 20453461.

17. Rabe KG, Matsuse H, Jackson A, Segal NA. Evaluation of the Combined Application of Neuromuscular Electrical Stimulation and Volitional Contractions on Thigh Muscle Strength, Knee Pain, and Physical Performance in Women at Risk for Knee Osteoarthritis: A Randomized Controlled Trial. PM R. 2018;10(12):1301-10. Epub 2018/06/01. doi: 10.1016/j.pmrj.2018.05.014. PubMed PMID: 29852286; PubMed Central PMCID: PMCPMC6719317.

18. Matsuse H, Shiba N, Takano Y, Yamada S, Ohshima H, Tagawa Y. Cycling exercise to resist electrically stimulated antagonist increases oxygen uptake in males: pilot study. J Rehabil Res Dev. 2013;50(4):545-54. PubMed PMID: 23934874.

19. Ohmoto M MH, Takano Y, Yamada S, Ohshima H, Tagawa Y, Shiba N. Oxygen

Uptake during Aerobic Cycling Exercise Simultaneously Combined with Neuromuscular Electrical Stimulation of Antagonists. J Nov Physiother. 2013:3-6.

20. Gaesser GA, Poole DC. The slow component of oxygen uptake kinetics in humans. Exerc Sport Sci Rev. 1996;24:35-71. Epub 1996/01/01. PubMed PMID: 8744246.

21. Matsuse H, Segal NA, Rabe KG, Shiba N. The Effect of Neuromuscular Electrical Stimulation during Walking on Muscle Strength and Knee Pain in Obese Women with Knee Pain: A Randomized Controlled Trial. Am J Phys Med Rehabil. 2019. Epub 2019/10/09. doi: 10.1097/PHM.0000000000001319. PubMed PMID: 31592880.

22. Oates BR, Glover EI, West DW, Fry JL, Tarnopolsky MA, Phillips SM. Low-volume resistance exercise attenuates the decline in strength and muscle mass associated with immobilization. Muscle Nerve. 2010;42(4):539-46. Epub 2010/07/27. doi: 10.1002/mus.21721. PubMed PMID: 20658567.

23. Alkner BA, Tesch PA. Efficacy of a gravity-independent resistance exercise device as a countermeasure to muscle atrophy during 29-day bed rest. Acta Physiol Scand. 2004;181(3):345-57. Epub 2004/06/16. doi: 10.1111/j.1365-201X.2004.01293.x. PubMed PMID: 15196095.

24. Berg HE, Tesch A. A gravity-independent ergometer to be used for resistance training in space. Aviat Space Environ Med. 1994;65(8):752-6. Epub 1994/08/01. PubMed PMID: 7980338.

25. Burke D, Gorman E, Stokes D, Lennon O. An evaluation of neuromuscular electrical stimulation in critical care using the ICF framework: a systematic review and meta-analysis. Clin Respir J. 2016;10(4):407-20. Epub 2014/10/30. doi: 10.1111/crj.12234. PubMed PMID: 25353646.

26. Iwanaga S, Hashida R, Takano Y, Bekki M, Nakano D, Omoto M, et al. Hybrid Training System Improves Insulin Resistance in Patients with Nonalcoholic Fatty Liver Disease: A Randomized Controlled Pilot Study. Tohoku J Exp Med. 2020;252(1):23-32. Epub 2020/08/31. doi: 10.1620/tjem.252.23. PubMed PMID: 32863329.

27. Tsukada Y, Matsuse H, Shinozaki N, Takano Y, Nago T, Shiba N. Combined Application of Electrically Stimulated Antagonist Muscle Contraction and Volitional Muscle Contraction Prevents Muscle Strength Weakness and Promotes Physical Function Recovery After Total Knee Arthroplasty: A Randomized Controlled Trial. Kurume Med J. 2020;65(4):145-54. Epub 2019/11/15. doi: 10.2739/kurumemedj.MS654007. PubMed PMID: 31723080.

28. Numata H, Nakase J, Inaki A, Mochizuki T, Oshima T, Takata Y, et al. Effects of the belt electrode skeletal muscle electrical stimulation system on lower extremity skeletal muscle activity: Evaluation using positron emission tomography. J Orthop Sci.

2016;21(1):53-6. Epub 2016/01/13. doi: 10.1016/j.jos.2015.09.003. PubMed PMID: 26755387.

29. Forestieri P, Bolzan DW, Santos VB, Moreira RSL, de Almeida DR, Trimer R, et al. Neuromuscular electrical stimulation improves exercise tolerance in patients with advanced heart failure on continuous intravenous inotropic support use-randomized controlled trial. Clin Rehabil. 2018;32(1):66-74. Epub 2017/06/22. doi: 10.1177/0269215517715762. PubMed PMID: 28633534.

30. Dalise S, Cavalli L, Ghuman H, Wahlberg B, Gerwig M, Chisari C, et al. Biological effects of dosing aerobic exercise and neuromuscular electrical stimulation in rats. Sci Rep. 2017;7(1):10830. Epub 2017/09/09. doi: 10.1038/s41598-017-11260-7. PubMed PMID: 28883534; PubMed Central PMCID: PMCPMC5589775.

31. Fontes Cerqueira TC, Cerqueira Neto ML, Cacau LAP, Oliveira GU, Silva Junior WMD, Carvalho VO, et al. Ambulation capacity and functional outcome in patients undergoing neuromuscular electrical stimulation after cardiac valve surgery: A randomised clinical trial. Medicine (Baltimore). 2018;97(46):e13012. Epub 2018/11/16. doi: 10.1097/MD.0000000000013012. PubMed PMID: 30431575; PubMed Central PMCID: PMCPMC6257613.

32. Hainaut K, Duchateau J. Neuromuscular electrical stimulation and voluntary exercise. Sports Med. 1992;14(2):100-13. Epub 1992/08/01. doi: 10.2165/00007256-199214020-00003. PubMed PMID: 1509225.

33. Theurel J, Lepers R, Pardon L, Maffiuletti NA. Differences in cardiorespiratory and neuromuscular responses between voluntary and stimulated contractions of the quadriceps femoris muscle. Respir Physiol Neurobiol. 2007;157(2-3):341-7. Epub 2007/01/11. doi: 10.1016/j.resp.2006.12.002. PubMed PMID: 17210271.

34. Liu M, Luo J, Zhou J, Zhu X. Intervention effect of neuromuscular electrical stimulation on ICU acquired weakness: A meta-analysis. Int J Nurs Sci. 2020;7(2):228-37. Epub 2020/07/21. doi: 10.1016/j.ijnss.2020.03.002. PubMed PMID: 32685621; PubMed Central PMCID: PMCPMC7355203.

35. Nakamura K, Nakano H, Naraba H, Mochizuki M, Hashimoto H. Early rehabilitation with dedicated use of belt-type electrical muscle stimulation for severe COVID-19 patients. Crit Care. 2020;24(1):342. Epub 2020/06/17. doi: 10.1186/s13054-020-03080-5. PubMed PMID: 32539827; PubMed Central PMCID: PMCPMC7294763.

36. Swain DP, Leutholtz BC. Heart rate reserve is equivalent to %VO2 reserve, not to %VO2max. Med Sci Sports Exerc. 1997;29(3):410-4. Epub 1997/03/01. doi: 10.1097/00005768-199703000-00018. PubMed PMID: 9139182.
