# Supplementary material for: Electrically stimulated eccentric contraction during non-weight bearing knee bending exercise in the supine position increases oxygen uptake: A randomized, controlled, exploratory crossover trial
Source: PLoS One. 2021 Nov 18;16(11):e0259856. doi: 10.1371/journal.pone.0259856 (PMC8601547; doi:10.1371/journal.pone.0259856)
Supplement: Attachment — Submitted filename: comments.doc [file pone.0259856.s006.doc]

**Manuscript Number**: PONE-D-21-17794

Overall, the idea of research is very interesting. However, there are some comments and suggestions.

**Revision Suggestions**

**Title**

 I suggest some changes in the title as follow:

- Electrically stimulated eccentric contraction during non-weight bearing knee bending exercise increases oxygen uptake: a randomized controlled crossover trial

**Abstract**

The abstract is better designed as background (a brief one), purpose, methods (participants, study groups, outcome measures), results and conclusion.

**Introduction**

Line 82: please cite the references as (5-7)

Line 84: please cite the references as (8,9)

**Materials and methods**

- Ethical approval and trail registration are addressed.
- You need to clarify the study groups and the difference between them
- Line 105: correct (SD = 8.5)) to (SD = 8.5)
- Sample size was not calculated. Don’t you think the sample of 10 participants is relatively small and the results can't be generalized?
- The validity and reliability of assessment methods not stated
- For all figures, write the figure label under the corresponding figure. The label should not include statistical results. For example Fig.4. Comparing of changes of oxygen uptake.
- No need for fig 2 as figure 3 is sufficient

**Statistical analysis:** Satisfying

**Results:** Satisfying

**Discussion**: Satisfying

**References**

You need to update some references

**References**

Update some references is required
