# Supplementary material for: Electrically stimulated eccentric contraction during non-weight bearing knee bending exercise in the supine position increases oxygen uptake: A randomized, controlled, exploratory crossover trial
Source: PLoS One. 2021 Nov 18;16(11):e0259856. doi: 10.1371/journal.pone.0259856 (PMC8601547; doi:10.1371/journal.pone.0259856)
Supplement: Attachment — Submitted filename: renamed_16a0c.docx [file pone.0259856.s007.docx]

Electrically stimulated eccentric contraction during knee bending exercise in the dorsal position on a bed increases oxygen uptake: a randomized controlled crossover trial

1Hiroshi Tajima, M.D., 1Hiroo Matsuse, M.D., Ph.D., 1Ryuki Hashida, M.D., Ph.D., 1Takeshi Nago, M.D., Ph.D., 1Masafumi Bekki, M.D., Ph.D.,1Sohei Iwanaga, M.D., Ph.D., 1Eriko Higashi, M.D., 1Naoto Shiba, M.D., Ph.D.

1 Rehabilitation center, Kurume University, 67 Asahi-machi, Kurume, Fukuoka 830-0011, JAPAN

Address for corresponding with the author: Hiroo Matsuse, M.D., Ph.D.

Rehabilitation center, Kurume University, 67 Asahi-machi, Kurume, Fukuoka 830-0011, Japan. Tel: +81-(0)942-31-7568, Fax: +81-(0)942-46-6002

E-mail: matsuse_hiroh@kurume-u.ac.jp

Running title: Oxygen uptake during knee bending on a bed

Author contributions:

1.	Hiroyuki Tajima wrote the manuscript and mainly carried out all the experiments.

2.	Naoto Shiba and Hiroo Matsuse managed the exercise method combined with hybrid training system.

3.	Masafumi Bekki, Sohei Iwanaga, and Eriko Higashi measured and calculated the data.

4.	Takeshi Nago and Ryuki Hashida managed this experiment.

Disclosures:

This research did not receive any specific grant from funding agencies in the public, commercial, or not-for-profit sectors. All authors declare that they have no competing interests in this study.

Abbreviation’s list:

ES, electrical stimulation; HTS, hybrid training system; HR; heart rate; NMES, neuromuscular electrical stimulation; METs, Metabolic equivalents; RER, respiratory exchange ratio; VC, volitional contraction; VCO2, carbon dioxide output; VO2, oxygen uptake

**Abstract**

It is well known that prolonged bed rest induces muscle weakness, muscle atrophy, cardiovascular deconditioning, bone loss, a loss of functional capacity, and the development of insulin resistance. Neuromuscular electrical stimulation is anticipated to be an interventional strategy for disuse due to bed rest. A hybrid training system (HTS), synchronized neuromuscular electrical stimulation for voluntary exercise using an articular motion sensor, may increase the exercise load though bed rest. We assessed oxygen uptake or heart rate during knee bending exercise on a bed both simultaneously combined with HTS and without HTS to evaluate exercise intensity on different days in ten healthy subjects (8 men and 2 women) by a randomized controlled crossover trial. The values of relative oxygen uptake during knee bending exercise with HTS were significantly greater than those during knee bending exercise without HTS (7.29 ± 0.91 ml/kg/min vs. 8.29 ± 1.06 ml/kg/min; p = 0.0115). That increment with HTS was an average of 14.42 ± 13.99%. Metabolic equivalents during knee bending exercise with HTS and without HTS were 2.08 ± 0.26 and 2.39 ± 0.30, respectively. The values of heart rate during knee bending exercise with HTS were significantly greater than those during knee bending exercise without HTS (80.82 ± 9.19 bpm vs. 86.36 ± 5.50 bpm; p = 0.0153). HTS could increase exercise load during knee bending exercise which is easy to implement on a bed. HTS might be a useful technique as a countermeasure against the disuse due to bed rest, for example during acute care or the quarantine for infection prophylaxis.

Key words: Analysis of expired gas, Bed rest, Disuse, Metabolic equivalents, Neuromuscular electrical stimulation

**Introduction**

It is well known that muscle atrophy, cardiovascular deconditioning, bone loss, a loss of functional capacity, and the development of insulin resistance due to disuse occurs in astronauts (1, 2). Bed rest causes similar disuse although bed rest has been prescribed in the past for several other clinical conditions (3). Strength training or aerobic exercise programs are expected to prevent disuse atrophy and deconditioning (4). However, the evidence regarding interventions involving either strength training alone or aerobic exercise alone remains uncertain (4). Moreover, some patients in acute care hospitals have difficulty even standing. Some patients aren’t able to do exercise at intensity 3 MET a such as walking. We need relatively efficient and simple exercise that can be performed on a bed to prevent disuse during bed rest.

Neuromuscular electrical stimulation (NMES) is widely used to lessen the complications of disuse during spaceflight or bed rest by increasing muscle strength and mass (5) (6) (7). Moreover, it is well known that NMES upregulates glucose metabolism and peak oxygen uptake (8) (9). In addition, the combined application of electrical stimulation (ES) and volitional contractions (VC) is said to be more effective than ES or VC alone (10). The hybrid training system is one of the techniques combining VC and ES, and is able to be used during exercises such as ergometer walking. We have reported that ergometer exercise using HTS leads to a greater increase in metabolic cost (11, 12). Knee bending exercise, one of the common exercises on a bed, is recommended for patients with disuse syndrome who are not able to walk in a clinical situation. Therefore, we hypothesize that by combining HTS with knee bending exercise may provide exercise intensity that can easily be performed on a bed. It may be a useful exercise technique for the prevention of disuse due to bed rest.

The purpose of the present study is to compare the metabolic cost between knee bending exercise on a bed with and without HTS by analyzing expired gas.

**Methods**

**Subjects**

This study protocol was approved by the Ethics Committee of Kurume University (No.19209) and registered at **UMIN** Clinical Trials Registry (UMIN000039336). Following approval, informed consent was obtained from 10 healthy (8 men and 2 women, age 28.9 years (SD = 8.5)) who had reviewed the goals of the study and agreed to participate. Inclusion criteria required that the subjects undergo an examination for normal physical fitness, strength, sensation, and range of motion according to the criteria of the Japanese Orthopedic Association. Exclusion criteria were that the subjects had: a musculoskeletal problem, adverse medical history affecting cardiorespiratory function, or some kind of medical treatment. They were also not to participate in any regular sports activities during the study period.

**Design**

We performed two cardiopulmonary exercise tests in the clinical laboratory of the rehabilitation center by a randomized, controlled, crossover trial (Fig.1). Environmental conditions were similar for all exercise tests (21 to 24 degrees centigrade, 45 to 55 % relative humidity). We measured gas exchange while subjects did knee bending exercises with a RISTA board (NIPPON SIGMAX Co, Tokyo, Japan) with the lowest exercise resistance out of three phases using the following protocol after a rest for 30 minutes. We used the device to achieve a stable knee bending exercise on a flat surface. During the tests, gas exchange data was collected continuously with an automated breath by breath system (AE-100i, Minato Medical Science Co. Ltd., Osaka, Japan) using the standard technique. Also, heart rates (HR, beats/min) were continuously monitored by electrocardiogram during the tests. O2 and carbon dioxide output (CO2) were calculated and recorded during the following exercise tests.

**Intervention**

The knee bending exercise test was performed as two tests (with HTS or without HTS) for analysis of expired gas. At first subjects rested for five minutes in a sitting position for rest. Next, subjects performed the knee bending exercise at the speed of 1Hz using a metronome for 5 minutes with HTS or without HTS on a flat surface after warming up at a slow speed for 3 minutes. The sequence of the knee bending exercise tests with or without HTS was performed at random, and on different days separated by an interval of one week as a wash out period. When participants were enrolled, the staff who was not involved in intervention and evaluation randomized the intervention sequence using a random number generator and sealed opaque envelopes with a block size of 5. We set the total exercise time for 10 minutes including 3 minutes warm up and 2 minutes cool down in consideration of muscle fatigue from ES. The knee joint range of motion was set at a nearly 90° arc that extended from 20° to 110° (0° indicating full knee extension). At the onset of constant-load exercise, O2 increases in healthy individual’s mono-exponentially with a time constant to achieve a steady state below the lactate threshold within about 3 minutes (13). Therefore, we averaged the data of HR and expired gas during the last 2 minutes of each test and used them for data analysis.

**HTS protocol**

HTS was combined with knee bending exercise on a flat surface with an electrical stimulator (HIZA TRAINER, EU-JLM50S, Panasonic Corporation, 1006 Ohaza-Kadoma, Kadoma City, Osaka, Japan). This provided a constant voltage stimulus to the skin electrodes (regulated voltage)”. This stimulator is household medical equipment including a wrapping band to fix the device and acceleration sensors or electrodes during exercise. The acceleration sensors act as joint motion sensors (EWTS9PD, Home Appliances Development Center Corporate Engineering Division, Appliances Company Panasonic Corporation 2-3-1-2 Noji-higashi, Kusatsu City, Shiga, Japan) and are placed on the front of each leg 88 mm above the patellar edge. The sensors analyze the algorithm of each gait pattern, and stimulate the flexor or extensor in accordance with the motion of each bilateral knee joint during knee bending exercise (Figs 2 and 3). Electrodes (Sekisui Plastics Co., Tokyo, Japan) coated with an oxidation-resistant silver-carbon compound and were placed over the quadriceps on the anterior thigh, and over the hamstrings on the posterior thigh with low impedance gel. The size of the electrodes was 15cm × 5cm for the quadriceps and 10cm × 6cm for the hamstrings. ES stimulated the quadriceps during knee flexion. Conversely, ES stimulated the hamstrings during knee extension. Therefore, the electrically stimulated muscles were eccentric contractions. ES parameters were based on a standard Russian waveform in which a 5,000 Hz carrier frequency is modulated at 40 Hz (2.4 ms on, 22.6 ms off) to deliver a rectangular voltage biphasic pulse. ES intensity was set to approximately 80% of the subject’s maximum tolerance. This intensity has been reported to successfully improve muscle strength and mass without causing pain or numbness (14-16).

**Fig 2. Schematic model of hybrid training system.**

Subjects lay in a supine position on a flat surface with their quadriceps electrically stimulated as they attempted to bend their knee and their hamstrings electrically stimulated as they attempted to extend their knee. The timing of the electrical stimulation was controlled by a joint motion sensor attached to the knee.

**Fig 3. Exercise scene.**

Subjects lay on their back on a bed. They bent and stretched their left and right lower limbs alternately.

**Statistical Analysis**

All variables are presented as means ± SD. This study was not set to the sample size for an exploratory study. The original study plan recruited 20 participants, but we analyzed this study because we were able to achieve this study purpose from ten participants. We compared the participants’ characteristics and rest values (relative VO2, VCO2, and HR) between groups (two sequences) by Student’s t-test, or Fisher's exact test. Values for relative VO2, VCO2, HR, and respiratory exchange ratio (RER) during exercise test were analyzed with linear mixed model that included fixed effects of treatment (with HTS or without HTS), period, and sequence and random effect of subjects. Next, values for relative VO2, VCO2, and HR were assessed using a student t-test as a post hoc test in order to compare the differences between the knee bending exercise tests with and without HTS for the main purpose of this study. All the statistical analyses were performed using JMP Version 14.0 statistical software (SAS Institute Inc., Cary, NC, USA) and p values < 0.05 were considered to be statistically significant.

**Results**

There were not any problems and adverse events in the exercise tests for subjects such as not being able to continue from pain or fatigue. Each five subjects participated in two sequences to be shown in the flow diagram (Fig.1), and all ten subjects finished all tests and were analyzed.

There were no significant differences at participants’ characteristics and rest values (relative VO2, VCO2, and HR) between groups (Table1). There were no significant differences at period and sequence in values for relative VO2, VCO2, and HR during exercise test though there was significant difference at treatment (with HTS or without HTS). As a primary outcome, the values of relative VO2 during knee bending exercise with HTS were significantly greater than those during knee bending exercise without HTS (7.29 ± 0.91 ml/kg/min vs 8.29 ± 1.06 ml/kg/min; p = 0.0115) (Fig 4). The estimated effect size (95% confidence interval) was 0.4219 (0.0520-0.9839). The increment using HTS was an average of 14.42 ± 13.99%. Metabolic equivalents (METs) during knee bending exercise with HTS and without HTS were 2.08 ± 0.26 and 2.39 ± 0.30, respectively. The values of relative VCO2 during knee bending exercise with HTS were significantly greater than those during knee bending exercise without HTS (7.29 ± 0.91 ml/kg/min vs 8.29 ± 1.06 ml/kg/min; p = 0.0423) (Fig 5). There was not a significant difference between the values of RER during knee bending exercise with HTS and without HTS (0.94 ± 0.08 vs 0.94 ± 0.10; p = 0.86). The values of HR during knee bending exercise with HTS were significantly greater than without HTS (80.82 ± 9.19 bpm vs 86.36 ± 5.50 bpm; p = 0.0153) (Fig 6).

**Fig.4. Changes of oxygen uptake (VO2).**

The values of relative VO2 during knee bending exercise with HTS were significantly greater than those during knee bending exercise without HTS (7.29 ± 0.91 ml/kg/min vs 8.29 ± 1.06 ml/kg/min; p = 0.0115). The increment by HTS was an average of 14.42 ± 13.99%. O2, oxygen consumption; HTS, hybrid training system.

**Fig.5. Changes of carbon dioxide production (VCO2).**

The values of relative VCO2 during knee bending exercise with HTS were significantly greater than those during knee bending exercise without HTS (7.29 ± 0.91 ml/kg/min vs 8.29 ± 1.06 ml/kg/min; p = 0.0423). CO2, carbon dioxide production; HTS, hybrid training system.

**Fig.6. Changes of heart rate (HR).**

The values of HR during knee bending exercise with HTS were significantly greater than those during knee bending exercise without HTS (80.82 ± 9.19 bpm vs 86.36 ± 5.50 bpm; p = 0.0153). HR, heart rate; HTS, hybrid training system.

**Discussion**

This is the first study of the metabolic cost of knee bending exercise combined with NMES that is easily done in a reclining position. This study showed that HTS, utilizing electrically stimulated eccentric muscle contractions as a resistance to voluntary exercise, increased exercise intensity during knee bending exercise on a bed. HTS may be a useful exercise technique to prevent disuse during bed rest by providing augmentation of local mechanical stress and systemic metabolic stress.

The decrement in mechanical stress caused by bed rest or spaceflight causes muscle weakness, muscular atrophy, bone atrophy, and the loss of cardiovascular capacity. Resistance exercise is an effective countermeasure for the prevention of muscle atrophy (2) even if it is relatively low volume of resistance exercise (17) (18). However, Bryan. R. Oates et al. (17) used an exercise intensity of 80% of maximal with resistance exercise. It is difficult for clinical patients to achieve adequate exercise intensity on a bed. The flywheel ergometer has been used for resistance training in space flight. The resistance was provided independent of gravity by using the inertial focus of a flywheel (19). B. A. Alkner and P. A. Tesch (18) reported the utility of the flywheel ergometer on the bed. However, it is difficult to use a flywheel ergometer for clinical applications because it is a special device. NMES is widely used in clinic settings and can easily generate sufficient muscle contractions without relying on gravity. Therefore, NMES could be useful as a countermeasure against disuse due to bed rest. It could be especially useful when effective voluntary exercise is not possible due to disturbance of consciousness or strict medical management (20). However, VC is undoubtedly the most basic effective countermeasure for disuse. In this study, the knee bending exercise which was easily performed in a supine position on a flat surface was approximately 2 METs in exercise intensity. Furthermore, we can make up for the detriments and use them to advantage by combining VC with ES (11) (12, 21, 22). Moreover, ES should ideally be applied synchronously with voluntary exercise to obtain synergistic benefits (2). In this study, we could increase exercise intensity by approximately 14.4% of the by combining ES with the knee bending exercise on a flat surface. Therefore, we could strengthen the exercise intensity even though in a supine position on a flat surface by using HTS more effectively.

In addition, aerobic exercise is an effective countermeasure against the loss of cardiovascular capacity associated with disuse (2). Generally, the metabolic rate during complete bed rest is less than 1 MET. Physical activity is undoubtedly the most effective countermeasure against disuse. The knee bending exercise in this study resulted in approximately 2 METs of metabolic rate. NMES stimulates metabolic response (23) and energy consumption, carbohydrate oxidation, and whole-body glucose uptake (9). Therefore, we may use NMES as aerobic exercise to improve physical capacity (24). However, NMES did not change maximum capacity although aerobic exercise did in rats (25). Additionally, NMES did not improve functional capacity in cardiac valve surgery patients in the immediate postoperative period (26). Therefore, the aerobic exercise effect of NMES as a countermeasure against disuse due to bed rest may be limited. ES activation is considered nonselective with regard to the type of motor unit and synchrony, and it preferentially activates Type II fibers as compared with VC (27). Jean Theurel et al. showed that the O2 and RER were significantly higher during ES compared with VC to compare respiratory gas exchange variables between equal-intensity (i.e., same force output) ES and VC of the quadriceps (28). They considered that these findings probably reflect differences in the patterns of motor unit recruitment between ES and VC. On the other hand, in this study, there was not a significant difference between the values of RER between HTS and VC although O2 during HTS was significantly greater than for VC (without HTS). Combining VC with ES simultaneously seems to have been responsible for those results. Similarly, in a past study, we showed that HTS causes an increase of approximately 4.4% of O2 during unloaded cycling without a significant difference in RER (11). Moreover, HTS caused an increase of approximately 21.1% of O2 during aerobic cycle ergometer exercise at moderate-intensity (12). In this study, HTS caused an increase of approximately 14.4% of O2 during knee bending exercise on a flat surface. In addition, the METs for these studies were approximately 2.39, which is equivalent to light effort such as housework or light exercise. Moreover, a utility of NMES was shown in disuse prevention or the complications prevention in the acute care (29). Furthermore, NMES is expected as one of the effective exercise therapies during the quarantine for COVID-19 patients(30). These findings showed that we could strengthen the metabolic stress from increased physical activity by combining HTS with the knee bending exercise that is easily performed by bed ridden patients (e.g., rest during the acute care or the quarantine for prevention of infection spread).

There are a few potential limitations of this study. There were few female subjects, therefore a future study including more women will be necessary. The purpose of this study was to evaluate the basic gas exchange response to HTS combined with knee bending exercise as a technique to provide physical activity as a countermeasure against disuse due to bed rest. For estimating exercise intensity, oxygen uptake reserve or heart rate reserve is preferable (31). However, we did not evaluate peak oxygen consumption or peak heart rate in each subject. In addition, we did not evaluate lactic acid or glucose to evaluate the metabolic response after having done strict nutritional management. For the investigation of clinical response, it is necessary to include clinical patients. Additionally, muscle contraction quantity and time influence kinetic oxygen uptake. Therefore, various protocols of electrical stimulation (such as modification of frequency, pulse width, or duty cycle) will be necessary to evaluate the effect of HTS in a future study. Of course, we should perform a randomized, controlled trial design, long-term investigation study to investigate the efficacy of strength and cardiovascular capacity of HTS for patients with disuse.

**Conclusion**

These findings indicate that the combined application of VC and EC could lead to a greater increase in metabolic cost during knee bending exercise on a flat surface. Knee bending exercise simultaneously combined with HTS might be useful as a countermeasure against disuse due to bed rest by enforcing metabolic stress as well as mechanical stress.

**Table 1. Participants’ characteristics.**

| **Characteristics** | **Group A** | **Group B** | **p-value** |
| --- | --- | --- | --- |
| **Age (years)** | 27.6 ± 7.3 | 30.2 ± 10.3 | 0.6580 |
| Sex (male/female) | 4/1 | 4/1 | 1.00 |
| Body mass (kg) | 66.1 ± 8.6 | 59.8 ± 10.2 | 0.3244 |
| Height (cm) | 167.8 ± 5.9 | 168.4 ± 13.3 | 0.9335 |
| BMI (kg/m^2^) | 23.4 ± 1.6 | 21.1 ± 3.1 | 0.1861 |
| Rest VO_2_ (kg/cm^2^) | 3.85 ± 0.24 | 3.91 ± 0.66 | 0.8606 |
| Rest VCO_2_ (kg/cm^2^) | 3.52 ± 0.70 | 3.33 ± 0.80 | 0.6947 |
| Rest HR (bpm) | 68.6 ± 4.7 | 68.5 ± 9.4 | 0.9906 |

Mean ± SD*.* P-values were for comparing between groups by Student’s t-test, or Fisher's exact test.

Abbreviations: BMI, body mass index; Rest HR, heart rate in a sitting position; Rest VCO2, carbon dioxide output in a sitting position; Rest VO2, oxygen uptake in a sitting position

Acknowledgments

Financial Disclosures:

All authors have declared that no competing interest exist.

Funding/Support:

This research did not receive any specific grant from funding agencies in the public, commercial, or not-for-profit sectors.

Institutional Review

The study was designed in accordance with the ethical standards of the Helsinki Declaration of 1975 and received the approval of the Ethics Committee of Kurume University.

All procedures were fully explained to the participants who gave their written informed consent to participate.

Figure Legends

Fig.1．Flow Diagram

Fig.2. Schematic model of hybrid training system.

Subjects lay in a supine position on a flat surface with their quadriceps electrically stimulated as they attempted to bend their knee and their hamstrings electrically stimulated as they attempted to extend their knee. The timing of the electrical stimulation was controlled by a joint motion sensor attached to the knee.

Fig.3. Exercise scene.

Subjects lay on their back on a bed. They bent and stretched their left and right lower limbs alternately.

Fig.4. Changes of oxygen uptake (VO2).

The values of relative VO2 during knee bending exercise with HTS were significantly greater than those during knee bending exercise without HTS (7.29 ± 0.91 ml/kg/min vs 8.29 ± 1.06 ml/kg/min; p = 0.0115). The increment by HTS was an average of 14.42 ± 13.99%. O2, oxygen consumption; HTS, hybrid training system.

Fig.5. Changes of carbon dioxide production (VCO2).

The values of relative VCO2 during knee bending exercise with HTS were significantly greater than those during knee bending exercise without HTS (7.29 ± 0.91 ml/kg/min vs 8.29 ± 1.06 ml/kg/min; p = 0.0423). CO2, carbon dioxide production; HTS, hybrid training system.

Fig.6. Changes of heart rate (HR).

The values of HR during knee bending exercise with HTS were significantly greater than those during knee bending exercise without HTS (80.82 ± 9.19 bpm vs 86.36 ± 5.50 bpm; p = 0.0153). HR, heart rate; HTS, hybrid training system.

References

1.	Nagaraja MP, Jo H. The Role of Mechanical Stimulation in Recovery of Bone Loss-High versus Low Magnitude and Frequency of Force. Life (Basel). 2014;4(2):117-30.

2.	Valenzuela PL, Morales JS, Pareja-Galeano H, Izquierdo M, Emanuele E, de la Villa P, et al. Physical strategies to prevent disuse-induced functional decline in the elderly. Ageing Res Rev. 2018;47:80-8.

3.	Bloomfield SA. Changes in musculoskeletal structure and function with prolonged bed rest. Med Sci Sports Exerc. 1997;29(2):197-206.

4.	Voet NB, van der Kooi EL, van Engelen BG, Geurts AC. Strength training and aerobic exercise training for muscle disease. Cochrane Database Syst Rev. 2019;12:CD003907.

5.	Maffiuletti NA, Green DA, Vaz MA, Dirks ML. Neuromuscular Electrical Stimulation as a Potential Countermeasure for Skeletal Muscle Atrophy and Weakness During Human Spaceflight. Front Physiol. 2019;10:1031.

6.	Brower RG. Consequences of bed rest. Crit Care Med. 2009;37(10 Suppl):S422-8.

7.	Dirks ML, Wall BT, van Loon LJC. Interventional strategies to combat muscle disuse atrophy in humans: focus on neuromuscular electrical stimulation and dietary protein. J Appl Physiol (1985). 2018;125(3):850-61.

8.	Gomes Neto M, Oliveira FA, Reis HF, de Sousa Rodrigues E, Jr., Bittencourt HS, Oliveira Carvalho V. Effects of Neuromuscular Electrical Stimulation on Physiologic and Functional Measurements in Patients With Heart Failure: A SYSTEMATIC REVIEW WITH META-ANALYSIS. J Cardiopulm Rehabil Prev. 2016;36(3):157-66.

9.	Hamada T, Hayashi T, Kimura T, Nakao K, Moritani T. Electrical stimulation of human lower extremities enhances energy consumption, carbohydrate oxidation, and whole body glucose uptake. J Appl Physiol (1985). 2004;96(3):911-6.

10.	Paillard T. Combined application of neuromuscular electrical stimulation and voluntary muscular contractions. Sports Med. 2008;38(2):161-77.

11.	Matsuse H, Shiba N, Takano Y, Yamada S, Ohshima H, Tagawa Y. Cycling exercise to resist electrically stimulated antagonist increases oxygen uptake in males: pilot study. J Rehabil Res Dev. 2013;50(4):545-54.

12.	Ohmoto M MH, Takano Y, Yamada S, Ohshima H, Tagawa Y, Shiba N. Oxygen Uptake during Aerobic Cycling Exercise Simultaneously Combined with Neuromuscular Electrical Stimulation of Antagonists. J Nov Physiother. 2013:3-6.

13.	Gaesser GA, Poole DC. The slow component of oxygen uptake kinetics in humans. Exerc Sport Sci Rev. 1996;24:35-71.

14.	Matsuse H, Segal NA, Rabe KG, Shiba N. The Effect of Neuromuscular Electrical Stimulation during Walking on Muscle Strength and Knee Pain in Obese Women with Knee Pain: A Randomized Controlled Trial. Am J Phys Med Rehabil. 2019.

15.	Takano Y, Haneda Y, Maeda T, Sakai Y, Matsuse H, Kawaguchi T, et al. Increasing muscle strength and mass of thigh in elderly people with the hybrid-training method of electrical stimulation and volitional contraction. Tohoku J Exp Med. 2010;221(1):77-85.

16.	Rabe KG, Matsuse H, Jackson A, Segal NA. Evaluation of the Combined Application of Neuromuscular Electrical Stimulation and Volitional Contractions on Thigh Muscle Strength, Knee Pain, and Physical Performance in Women at Risk for Knee Osteoarthritis: A Randomized Controlled Trial. PM R. 2018;10(12):1301-10.

17.	Oates BR, Glover EI, West DW, Fry JL, Tarnopolsky MA, Phillips SM. Low-volume resistance exercise attenuates the decline in strength and muscle mass associated with immobilization. Muscle Nerve. 2010;42(4):539-46.

18.	Alkner BA, Tesch PA. Efficacy of a gravity-independent resistance exercise device as a countermeasure to muscle atrophy during 29-day bed rest. Acta Physiol Scand. 2004;181(3):345-57.

19.	Berg HE, Tesch A. A gravity-independent ergometer to be used for resistance training in space. Aviat Space Environ Med. 1994;65(8):752-6.

20.	Burke D, Gorman E, Stokes D, Lennon O. An evaluation of neuromuscular electrical stimulation in critical care using the ICF framework: a systematic review and meta-analysis. Clin Respir J. 2016;10(4):407-20.

21.	Iwanaga S, Hashida R, Takano Y, Bekki M, Nakano D, Omoto M, et al. Hybrid Training System Improves Insulin Resistance in Patients with Nonalcoholic Fatty Liver Disease: A Randomized Controlled Pilot Study. Tohoku J Exp Med. 2020;252(1):23-32.

22.	Tsukada Y, Matsuse H, Shinozaki N, Takano Y, Nago T, Shiba N. Combined Application of Electrically Stimulated Antagonist Muscle Contraction and Volitional Muscle Contraction Prevents Muscle Strength Weakness and Promotes Physical Function Recovery After Total Knee Arthroplasty: A Randomized Controlled Trial. Kurume Med J. 2020;65(4):145-54.

23.	Numata H, Nakase J, Inaki A, Mochizuki T, Oshima T, Takata Y, et al. Effects of the belt electrode skeletal muscle electrical stimulation system on lower extremity skeletal muscle activity: Evaluation using positron emission tomography. J Orthop Sci. 2016;21(1):53-6.

24.	Forestieri P, Bolzan DW, Santos VB, Moreira RSL, de Almeida DR, Trimer R, et al. Neuromuscular electrical stimulation improves exercise tolerance in patients with advanced heart failure on continuous intravenous inotropic support use-randomized controlled trial. Clin Rehabil. 2018;32(1):66-74.

25.	Dalise S, Cavalli L, Ghuman H, Wahlberg B, Gerwig M, Chisari C, et al. Biological effects of dosing aerobic exercise and neuromuscular electrical stimulation in rats. Sci Rep. 2017;7(1):10830.

26.	Fontes Cerqueira TC, Cerqueira Neto ML, Cacau LAP, Oliveira GU, Silva Junior WMD, Carvalho VO, et al. Ambulation capacity and functional outcome in patients undergoing neuromuscular electrical stimulation after cardiac valve surgery: A randomised clinical trial. Medicine (Baltimore). 2018;97(46):e13012.

27.	Hainaut K, Duchateau J. Neuromuscular electrical stimulation and voluntary exercise. Sports Med. 1992;14(2):100-13.

28.	Theurel J, Lepers R, Pardon L, Maffiuletti NA. Differences in cardiorespiratory and neuromuscular responses between voluntary and stimulated contractions of the quadriceps femoris muscle. Respir Physiol Neurobiol. 2007;157(2-3):341-7.

29.	Liu M, Luo J, Zhou J, Zhu X. Intervention effect of neuromuscular electrical stimulation on ICU acquired weakness: A meta-analysis. Int J Nurs Sci. 2020;7(2):228-37.

30.	Nakamura K, Nakano H, Naraba H, Mochizuki M, Hashimoto H. Early rehabilitation with dedicated use of belt-type electrical muscle stimulation for severe COVID-19 patients. Crit Care. 2020;24(1):342.

31.	Swain DP, Leutholtz BC. Heart rate reserve is equivalent to %VO2 reserve, not to %VO2max. Med Sci Sports Exerc. 1997;29(3):410-4.
