# Supplementary material for: Electrically stimulated eccentric contraction during non-weight bearing knee bending exercise in the supine position increases oxygen uptake: A randomized, controlled, exploratory crossover trial
Source: PLoS One. 2021 Nov 18;16(11):e0259856. doi: 10.1371/journal.pone.0259856 (PMC8601547; doi:10.1371/journal.pone.0259856)
Supplement: Attachment — Submitted filename: Response to Reviewers.docx [file pone.0259856.s009.docx]

PONE-D-21-17794R1

Electrically stimulated eccentric contraction during non-weight bearing knee bending exercise in the supine position on a bed increases oxygen uptake: a randomized, controlled, exploratory crossover trial

PLOS ONE

Dear Dr. Matsuse,

Thank you for submitting your manuscript to PLOS ONE. After careful consideration, we feel that it has merit but does not fully meet PLOS ONE’s publication criteria as it currently stands. Therefore, we invite you to submit a revised version of the manuscript that addresses the points raised during the review process.

Please submit your revised manuscript by Oct 28 2021 11:59PM. If you will need more time than this to complete your revisions, please reply to this message or contact the journal office at [plosone@plos.org](mailto:plosone@plos.org). When you're ready to submit your revision, log on to <https://www.editorialmanager.com/pone/> and select the 'Submissions Needing Revision' folder to locate your manuscript file.

Please include the following items when submitting your revised manuscript:

- A rebuttal letter that responds to each point raised by the academic editor and reviewer(s). You should upload this letter as a separate file labeled 'Response to Reviewers'.
- A marked-up copy of your manuscript that highlights changes made to the original version. You should upload this as a separate file labeled 'Revised Manuscript with Track Changes'.
- An unmarked version of your revised paper without tracked changes. You should upload this as a separate file labeled 'Manuscript'.

If you would like to make changes to your financial disclosure, please include your updated statement in your cover letter. Guidelines for resubmitting your figure files are available below the reviewer comments at the end of this letter.

If applicable, we recommend that you deposit your laboratory protocols in [protocols.io](http://protocols.io/) to enhance the reproducibility of your results. Protocols.io assigns your protocol its own identifier (DOI) so that it can be cited independently in the future. For instructions see: <https://journals.plos.org/plosone/s/submission-guidelines#loc-laboratory-protocols>. Additionally, PLOS ONE offers an option for publishing peer-reviewed Lab Protocol articles, which describe protocols hosted on [protocols.io](http://protocols.io/). Read more information on sharing protocols at <https://plos.org/protocols?utm_medium=editorial-email&utm_source=authorletters&utm_campaign=protocols>.

We look forward to receiving your revised manuscript.

Kind regards,

Walid Kamal Abdelbasset, Ph.D.
Academic Editor
PLOS ONE

Journal Requirements:

Please review your reference list to ensure that it is complete and correct. If you have cited papers that have been retracted, please include the rationale for doing so in the manuscript text, or remove these references and replace them with relevant current references. Any changes to the reference list should be mentioned in the rebuttal letter that accompanies your revised manuscript. If you need to cite a retracted article, indicate the article’s retracted status in the References list and also include a citation and full reference for the retraction notice.

A: We apologize that we were not able to confirm the reference list enough. We think the retracted paper was not included.

Because the paper of Omoto et al. (19) was not published in the pubmed, we have got a wrong reproduction. Also, the notation of his name was revised from the beginning of publication from Ohmoto to Omoto. We revised it in the latest information.

Because my paper (18) was a Web version, we updated it about the information (doi). Similarly, we confirmed the pubmed one by one and added a postscript to information of doi or updated the information. Also, we revised Epub date because the Epub date of the downloaded nbib file from pubmed was different from the PubMed site. We confirmed them one by one in the website of pubmed and revised them which have the information of Epub in the website of pubmed.

Because "PMC" was repeated in much PMCID, we deleted them. However, PMCID and doi were not published when we confirmed a latest PLOS ONE’s paper. Should we delete them? At least PMCID seemed to be unnecessary.

We download an endnote style (https://endnote.com/style_download/plos-public-library-of-science-all-journals/ ) from Submission Guidelines (https://journals.plos.org/plosone/s/submission-guidelines#loc-references) and used it, and, however, we think whether you are different from the “Vancouver” style a little. If there are unnecessary information and necessary information, please tell us.

Moreover, we added three references in this revised manuscript (20, 22, 33). Therefore, the reference numbers were changed.

We showed revised or changed points by a deficit as follows.

References

1.	Nagaraja MP, Jo H. The Role of Mechanical Stimulation in Recovery of Bone Loss-High versus Low Magnitude and Frequency of Force. Life (Basel). 2014;4(2):117-30. Epub 2014/11/06. doi: 10.3390/life4020117. PubMed PMID: 25370188; PubMed Central PMCID: PMC4187165.

2.	Valenzuela PL, Morales JS, Pareja-Galeano H, Izquierdo M, Emanuele E, de la Villa P, et al. Physical strategies to prevent disuse-induced functional decline in the elderly. Ageing Res Rev. 2018;47:80-8. Epub 2018/07/18. doi: 10.1016/j.arr.2018.07.003. PubMed PMID: 30031068.

3.	Bloomfield SA. Changes in musculoskeletal structure and function with prolonged bed rest. Med Sci Sports Exerc. 1997;29(2):197-206. Epub 1997/02/01. doi: 10.1097/00005768-199702000-00006. PubMed PMID: 9044223.

4.	Voet NB, van der Kooi EL, van Engelen BG, Geurts AC. Strength training and aerobic exercise training for muscle disease. Cochrane Database Syst Rev. 2019;12(12):CD003907. Epub 2019/12/07. doi: 10.1002/14651858.CD003907.pub5. PubMed PMID: 31808555; PubMed Central PMCID: PMC6953420.

5.	Maffiuletti NA, Green DA, Vaz MA, Dirks ML. Neuromuscular Electrical Stimulation as a Potential Countermeasure for Skeletal Muscle Atrophy and Weakness During Human Spaceflight. Front Physiol. 2019;10:1031. Epub 2019/08/29. doi: 10.3389/fphys.2019.01031. PubMed PMID: 31456697; PubMed Central PMCID: PMC6700209.

6.	Brower RG. Consequences of bed rest. Crit Care Med. 2009;37(10 Suppl):S422-8. Epub 2010/02/06. doi: 10.1097/CCM.0b013e3181b6e30a. PubMed PMID: 20046130.

7.	Dirks ML, Wall BT, van Loon LJC. Interventional strategies to combat muscle disuse atrophy in humans: focus on neuromuscular electrical stimulation and dietary protein. J Appl Physiol (1985). 2018;125(3):850-61. Epub 2017/9/28. doi: 10.1152/japplphysiol.00985.2016. PubMed PMID: 28970205.

8.	Gomes Neto M, Oliveira FA, Reis HF, de Sousa Rodrigues E, Jr., Bittencourt HS, Oliveira Carvalho V. Effects of Neuromuscular Electrical Stimulation on Physiologic and Functional Measurements in Patients With Heart Failure: A SYSTEMATIC REVIEW WITH META-ANALYSIS. J Cardiopulm Rehabil Prev. 2016;36(3):157-66. Epub 2016/01/20. doi: 10.1097/HCR.0000000000000151. PubMed PMID: 26784735.

9.	Hamada T, Hayashi T, Kimura T, Nakao K, Moritani T. Electrical stimulation of human lower extremities enhances energy consumption, carbohydrate oxidation, and whole body glucose uptake. J Appl Physiol (1985). 2004;96(3):911-6. Epub 2003/10/31. doi: 10.1152/japplphysiol.00664.2003. PubMed PMID: 14594864.

10.	Paillard T. Combined application of neuromuscular electrical stimulation and voluntary muscular contractions. Sports Med. 2008;38(2):161-77. doi: 10.2165/00007256-200838020-00005. PubMed PMID: 18201117.

11.	Dudley GA, Tesch PA, Miller BJ, Buchanan P. Importance of eccentric actions in performance adaptations to resistance training. Aviat Space Environ Med. 1991;62(6):543-50. PubMed PMID: 1859341.

12.	Seger JY, Thorstensson A. Electrically evoked eccentric and concentric torque-velocity relationships in human knee extensor muscles. Acta Physiol Scand. 2000;169(1):63-9. Epub 2000/04/12. doi: 10.1046/j.1365-201x.2000.00694.x. PubMed PMID: 10759612.

13.	Pain MT, Young F, Kim J, Forrester SE. The torque-velocity relationship in large human muscles: maximum voluntary versus electrically stimulated behaviour. J Biomech. 2013;46(4):645-50. Epub 2013/01/11. doi: 10.1016/j.jbiomech.2012.11.052. PubMed PMID: 23313275.

14.	Matsuse H, Shiba N, Umezu Y, Nago T, Tagawa Y, Kakuma T, et al. Muscle training by means of combined electrical stimulation and volitional contraction. Aviat Space Environ Med. 2006;77(6):581-5. PubMed PMID: 16780234.

15.	Iwasaki T, Shiba N, Matsuse H, Nago T, Umezu Y, Tagawa Y, et al. Improvement in knee extension strength through training by means of combined electrical stimulation and voluntary muscle contraction. Tohoku J Exp Med. 2006;209(1):33-40. doi: 10.1620/tjem.209.33. PubMed PMID: 16636520.

16.	Takano Y, Haneda Y, Maeda T, Sakai Y, Matsuse H, Kawaguchi T, et al. Increasing muscle strength and mass of thigh in elderly people with the hybrid-training method of electrical stimulation and volitional contraction. Tohoku J Exp Med. 2010;221(1):77-85. doi: 10.1620/tjem.221.77. PubMed PMID: 20453461.

17.	Rabe KG, Matsuse H, Jackson A, Segal NA. Evaluation of the Combined Application of Neuromuscular Electrical Stimulation and Volitional Contractions on Thigh Muscle Strength, Knee Pain, and Physical Performance in Women at Risk for Knee Osteoarthritis: A Randomized Controlled Trial. PM R. 2018;10(12):1301-10. Epub 2018/05/29. doi: 10.1016/j.pmrj.2018.05.014. PubMed PMID: 29852286; PubMed Central PMCID: PMC6719317.

18.	Matsuse H, Shiba N, Takano Y, Yamada S, Ohshima H, Tagawa Y. Cycling exercise to resist electrically stimulated antagonist increases oxygen uptake in males: pilot study. J Rehabil Res Dev. 2013;50(4):545-54. doi: 10.1682/jrrd.2012.04.0067. PubMed PMID: 23934874.

19.	Omoto M, Matsuse H, Takano Y, Yamada S, Ohshima H, Tagawa Y, et al. Oxygen Uptake during Aerobic Cycling Exercise Simultaneously Combined with Neuromuscular Electrical Stimulation of Antagonists. J Nov Physiother. 2013;3:185. Epub 2013/11/07. doi: 10.4172/2165-7025.1000185.

20.	Cameron S, Ball I, Cepinskas G, Choong K, Doherty TJ, Ellis CG, et al. Early mobilization in the critical care unit: A review of adult and pediatric literature. J Crit Care. 2015;30(4):664-72. Epub 2015/04/08. doi: 10.1016/j.jcrc.2015.03.032. PubMed PMID: 25987293.

21.	Gaesser GA, Poole DC. The slow component of oxygen uptake kinetics in humans. Exerc Sport Sci Rev. 1996;24:35-71. Epub 1996/01/01. PubMed PMID: 8744246.

22.	Ward AR, Shkuratova N. Russian electrical stimulation: the early experiments. Phys Ther. 2002;82(10):1019-30. Epub 2002/09/28. PubMed PMID: 12350217.

23.	Matsuse H, Segal NA, Rabe KG, Shiba N. The Effect of Neuromuscular Electrical Stimulation During Walking on Muscle Strength and Knee Pain in Obese Women With Knee Pain: A Randomized Controlled Trial. Am J Phys Med Rehabil. 2020;99(1):56-64. Epub 2019/10/09. doi: 10.1097/PHM.0000000000001319. PubMed PMID: 31592880.

24.	Tsukada Y, Matsuse H, Shinozaki N, Takano Y, Nago T, Shiba N. Combined Application of Electrically Stimulated Antagonist Muscle Contraction and Volitional Muscle Contraction Prevents Muscle Strength Weakness and Promotes Physical Function Recovery After Total Knee Arthroplasty: A Randomized Controlled Trial. Kurume Med J. 2020;65(4):145-54. Epub 2019/11/13. doi: 10.2739/kurumemedj.MS654007. PubMed PMID: 31723080.

25.	Oates BR, Glover EI, West DW, Fry JL, Tarnopolsky MA, Phillips SM. Low-volume resistance exercise attenuates the decline in strength and muscle mass associated with immobilization. Muscle Nerve. 2010;42(4):539-46. Epub 2010/07/27. doi: 10.1002/mus.21721. PubMed PMID: 20658567.

26.	Alkner BA, Tesch PA. Efficacy of a gravity-independent resistance exercise device as a countermeasure to muscle atrophy during 29-day bed rest. Acta Physiol Scand. 2004;181(3):345-57. Epub 2004/06/16. doi: 10.1111/j.1365-201X.2004.01293.x. PubMed PMID: 15196095.

27.	Berg HE, Tesch A. A gravity-independent ergometer to be used for resistance training in space. Aviat Space Environ Med. 1994;65(8):752-6. Epub 1994/08/01. PubMed PMID: 7980338.

28.	Burke D, Gorman E, Stokes D, Lennon O. An evaluation of neuromuscular electrical stimulation in critical care using the ICF framework: a systematic review and meta-analysis. Clin Respir J. 2016;10(4):407-20. Epub 2014/11/26. doi: 10.1111/crj.12234. PubMed PMID: 25353646.

29.	Iwanaga S, Hashida R, Takano Y, Bekki M, Nakano D, Omoto M, et al. Hybrid Training System Improves Insulin Resistance in Patients with Nonalcoholic Fatty Liver Disease: A Randomized Controlled Pilot Study. Tohoku J Exp Med. 2020;252(1):23-32. Epub 2020/08/31. doi: 10.1620/tjem.252.23. PubMed PMID: 32863329.

30.	Numata H, Nakase J, Inaki A, Mochizuki T, Oshima T, Takata Y, et al. Effects of the belt electrode skeletal muscle electrical stimulation system on lower extremity skeletal muscle activity: Evaluation using positron emission tomography. J Orthop Sci. 2016;21(1):53-6. Epub 2015/11/21. doi: 10.1016/j.jos.2015.09.003. PubMed PMID: 26755387.

31.	Forestieri P, Bolzan DW, Santos VB, Moreira RSL, de Almeida DR, Trimer R, et al. Neuromuscular electrical stimulation improves exercise tolerance in patients with advanced heart failure on continuous intravenous inotropic support use-randomized controlled trial. Clin Rehabil. 2018;32(1):66-74. Epub 2017/06/21. doi: 10.1177/0269215517715762. PubMed PMID: 28633534.

32.	Dalise S, Cavalli L, Ghuman H, Wahlberg B, Gerwig M, Chisari C, et al. Biological effects of dosing aerobic exercise and neuromuscular electrical stimulation in rats. Sci Rep. 2017;7(1):10830. Epub 2017/09/09. doi: 10.1038/s41598-017-11260-7. PubMed PMID: 28883534; PubMed Central PMCID: PMC5589775.

33.	Iwatsu K, Yamada S, Iida Y, Sampei H, Kobayashi K, Kainuma M, et al. Feasibility of neuromuscular electrical stimulation immediately after cardiovascular surgery. Arch Phys Med Rehabil. 2015;96(1):63-8. Epub 2014/09/09. doi: 10.1016/j.apmr.2014.08.012. PubMed PMID: 25218214.

34.	Fontes Cerqueira TC, Cerqueira Neto ML, Cacau LAP, Oliveira GU, Silva Junior WMD, Carvalho VO, et al. Ambulation capacity and functional outcome in patients undergoing neuromuscular electrical stimulation after cardiac valve surgery: A randomised clinical trial. Medicine (Baltimore). 2018;97(46):e13012. Epub 2018/11/16. doi: 10.1097/MD.0000000000013012. PubMed PMID: 30431575; PubMed Central PMCID: PMC6257613.

35.	Hainaut K, Duchateau J. Neuromuscular electrical stimulation and voluntary exercise. Sports Med. 1992;14(2):100-13. Epub 1992/08/01. doi: 10.2165/00007256-199214020-00003. PubMed PMID: 1509225.

36.	Theurel J, Lepers R, Pardon L, Maffiuletti NA. Differences in cardiorespiratory and neuromuscular responses between voluntary and stimulated contractions of the quadriceps femoris muscle. Respir Physiol Neurobiol. 2007;157(2-3):341-7. Epub 2006/12/15. doi: 10.1016/j.resp.2006.12.002. PubMed PMID: 17210271.

37.	Liu M, Luo J, Zhou J, Zhu X. Intervention effect of neuromuscular electrical stimulation on ICU acquired weakness: A meta-analysis. Int J Nurs Sci. 2020;7(2):228-37. Epub 2020/03/10. doi: 10.1016/j.ijnss.2020.03.002. PubMed PMID: 32685621; PubMed Central PMCID: PMC7355203.

38.	Nakamura K, Nakano H, Naraba H, Mochizuki M, Hashimoto H. Early rehabilitation with dedicated use of belt-type electrical muscle stimulation for severe COVID-19 patients. Crit Care. 2020;24(1):342. Epub 2020/06/15. doi: 10.1186/s13054-020-03080-5. PubMed PMID: 32539827; PubMed Central PMCID: PMC7294763.

39.	Swain DP, Leutholtz BC. Heart rate reserve is equivalent to %VO2 reserve, not to %VO2max. Med Sci Sports Exerc. 1997;29(3):410-4. Epub 1997/03/01. doi: 10.1097/00005768-199703000-00018. PubMed PMID: 9139182.

Additional Editor Comments (if provided):
Q: Is it safe to use fNMES for cardiac pateints?
A: It is safe if we use it appropriately. Of course, it is necessary to avoid the use to pacemaker wearers. However, we may not have any problem depending on how to use (Egger F, 2019). A useful of the electrical stimulation therapy in patients with chronic heart failure is shown in a systematic review (Gomes Neto M, 2016). Functional electrical stimulation of lower limb muscles is considered as an alternative mode of exercise training in chronic heart failure (Parissis J, 2015). Moreover, it has been shown that the use of NMES is safe in patients immediately after cardiovascular surgery (Iwatsu K, 2015).

We rewrote the sentence about the safety with a reference as follows.

“It has been shown that the use of NMES is safe in patients after cardiovascular surgery (Iwatsu K, 2015), but NMES did not improve functional capacity in cardiac valve surgery patients in the immediate postoperative period.”

References

Egger F, Hofer C, Hammerle FP, Lofler S, Nurnberg M, Fiedler L, et al. Influence of electrical stimulation therapy on permanent pacemaker function. Wien Klin Wochenschr. 2019;131(13-14):313-20. Epub 2019/04/27. doi: 10.1007/s00508-019-1494-5. PubMed PMID: 31025164.

Gomes Neto M, Oliveira FA, Reis HF, de Sousa Rodrigues E, Jr., Bittencourt HS, Oliveira Carvalho V. Effects of Neuromuscular Electrical Stimulation on Physiologic and Functional Measurements in Patients With Heart Failure: A SYSTEMATIC REVIEW WITH META-ANALYSIS. J Cardiopulm Rehabil Prev. 2016;36(3):157-66. Epub 2016/01/20. doi: 10.1097/HCR.0000000000000151. PubMed PMID: 26784735.

Parissis J, Farmakis D, Karavidas A, Arapi S, Filippatos G, Lekakis J. Functional electrical stimulation of lower limb muscles as an alternative mode of exercise training in chronic heart failure: practical considerations and proposed algorithm. Eur J Heart Fail. 2015;17(12):1228-30. Epub 2015/10/16. doi: 10.1002/ejhf.409. PubMed PMID: 26466970.

Iwatsu K, Yamada S, Iida Y, Sampei H, Kobayashi K, Kainuma M, et al. Feasibility of neuromuscular electrical stimulation immediately after cardiovascular surgery. Arch Phys Med Rehabil. 2015;96(1):63-8. Epub 2014/09/09. doi: 10.1016/j.apmr.2014.08.012. PubMed PMID: 25218214.

[Note: HTML markup is below. Please do not edit.]

Reviewers' comments:

Reviewer's Responses to Questions

**Comments to the Author**

1. If the authors have adequately addressed your comments raised in a previous round of review and you feel that this manuscript is now acceptable for publication, you may indicate that here to bypass the “Comments to the Author” section, enter your conflict of interest statement in the “Confidential to Editor” section, and submit your "Accept" recommendation.

Reviewer #1: All comments have been addressed

Reviewer #2: All comments have been addressed

Reviewer #3: (No Response)

2. Is the manuscript technically sound, and do the data support the conclusions?

The manuscript must describe a technically sound piece of scientific research with data that supports the conclusions. Experiments must have been conducted rigorously, with appropriate controls, replication, and sample sizes. The conclusions must be drawn appropriately based on the data presented.

Reviewer #1: Yes

Reviewer #2: Yes

Reviewer #3: Yes

3. Has the statistical analysis been performed appropriately and rigorously?

Reviewer #1: Yes

Reviewer #2: Yes

Reviewer #3: Yes

4. Have the authors made all data underlying the findings in their manuscript fully available?

The [PLOS Data policy](http://www.plosone.org/static/policies.action#sharing) requires authors to make all data underlying the findings described in their manuscript fully available without restriction, with rare exception (please refer to the Data Availability Statement in the manuscript PDF file). The data should be provided as part of the manuscript or its supporting information, or deposited to a public repository. For example, in addition to summary statistics, the data points behind means, medians and variance measures should be available. If there are restrictions on publicly sharing data—e.g. participant privacy or use of data from a third party—those must be specified.

Reviewer #1: Yes

Reviewer #2: Yes

Reviewer #3: Yes

5. Is the manuscript presented in an intelligible fashion and written in standard English?

PLOS ONE does not copyedit accepted manuscripts, so the language in submitted articles must be clear, correct, and unambiguous. Any typographical or grammatical errors should be corrected at revision, so please note any specific errors here.

Reviewer #1: Yes

Reviewer #2: Yes

Reviewer #3: Yes

6. Review Comments to the Author

Please use the space provided to explain your answers to the questions above. You may also include additional comments for the author, including concerns about dual publication, research ethics, or publication ethics. (Please upload your review as an attachment if it exceeds 20,000 characters)

Reviewer #1: Thanks to the authors for their detailed response and corrections. No further changes are suggested. the authors have done all required modifications.

A: Thank you for your polite review.

Reviewer #2:

1. Still the title is not in the PICO Format. Is it important to write "on a bed" try to shorten the title.

A: We deleted “on a bed".

　　“Electrically stimulated eccentric contraction during non-weight bearing knee bending exercise in the supine position increases oxygen uptake: a randomized, controlled, exploratory crossover trial”

2. short tile doesn't match the main title.
A: We changed the short title to “Oxygen uptake during knee bending exercise combined with electrical stimulation” to state the topic of the study.

(70 characters)

3. reference for the line 100-102

A: We think that the lower extremity bending exercise is not special, and it is the general physical therapy as an early mobilization which was recommended for critically ill patients. We revised the sentence as one of an early mobilization. We revised the sentence and added the reference (Cameron S, 2015) which shows the recommendation of the early mobilization for critically ill patients.

“Knee bending exercise, one of the common early mobilizations on a bed, is recommended for critically ill patients [20].”

Reference

Cameron S, Ball I, Cepinskas G, Choong K, Doherty TJ, Ellis CG, et al. Early mobilization in the critical care unit: A review of adult and pediatric literature. J Crit Care. 2015;30(4):664-72. Epub 2015/04/08. doi: 10.1016/j.jcrc.2015.03.032. PubMed PMID: 25987293.

4. line 2017: Add reference to support this point.

A: We added the reference about the standard Russian waveform (Ward AR, 2002). Moreover, we showed our past studies using this parameter.

“ES parameters were based on a standard Russian waveform (Ward AR, 2002) in which a 5,000 Hz carrier frequency is modulated at 40 Hz (2.4 ms on, 22.6 ms off) to deliver a rectangular voltage biphasic pulse [16][17-19, 23, 24].”

Reference

Ward AR, Shkuratova N. Russian electrical stimulation: the early experiments. Phys Ther. 2002;82(10):1019-30. Epub 2002/09/28. PubMed PMID: 12350217.

Reviewer #3: Minor revisions:
1. The following prior comment was not adequately addressed. Indicate the underlying covariance structure used in the linear mixed model and the criteria for selecting it.
The top 3 most common covariance structures are compound symmetry, unstructured, and autoregressive.

Ａ：It is the linear mixed model using covariance structure of compound symmetry as the result that we showed it in the manuscript. We talked with a statistician. As you pointed it out, we tried the linear mixed model in 3 covariance structures (compound symmetry, unstructured, and autoregressive) with the statistician. The compound symmetry was the best as a result that it was judged in BIC (Bayesian information criterion). Therefore, the results do not change. Thus, we added the following sentences to the part of the statistical analysis.

“Also, we tried the linear mixed model in 3 covariance structures (compound symmetry, unstructured, and autoregressive). The compound symmetry was the best covariance structure as a result that it was judged in BIC. Therefore, we selected the compound symmetry as the underlying covariance structure used in the linear mixed model.” （P15, L234-238）

2- Table 1: Include the corresponding percentage male.

Ａ：I'm sorry, we were not able to revise it. We showed it in a table as follows.

| Male sex, n (%) | 4 (80) | 4 (80) |
| --- | --- | --- |

Also, I'm sorry, there were a few mistakes in the table. We revised diagonal characters and bold-face.

7. PLOS authors have the option to publish the peer review history of their article ([what does this mean?](https://journals.plos.org/plosone/s/editorial-and-peer-review-process#loc-peer-review-history)). If published, this will include your full peer review and any attached files.
If you choose “no”, your identity will remain anonymous but your review may still be made public.

**Do you want your identity to be public for this peer review?** For information about this choice, including consent withdrawal, please see our [Privacy Policy](https://www.plos.org/privacy-policy).

Reviewer #1: No

Reviewer #2: **Yes:** Bishnu Dutta Acharya

Reviewer #3: No

[NOTE: If reviewer comments were submitted as an attachment file, they will be attached to this email and accessible via the submission site. Please log into your account, locate the manuscript record, and check for the action link "View Attachments". If this link does not appear, there are no attachment files.]

While revising your submission, please upload your figure files to the Preflight Analysis and Conversion Engine (PACE) digital diagnostic tool, <https://pacev2.apexcovantage.com/>. PACE helps ensure that figures meet PLOS requirements. To use PACE, you must first register as a user. Registration is free. Then, login and navigate to the UPLOAD tab, where you will find detailed instructions on how to use the tool. If you encounter any issues or have any questions when using PACE, please email PLOS at [figures@plos.org](mailto:figures@plos.org). Please note that Supporting Information files do not need this step.
